# Whole genome sequencing identifies structural variants contributing to hematologic traits in the NHLBI TOPMed program

**DOI:** 10.1038/s41467-022-35354-7

**Published:** 2022-12-08

**Authors:** Marsha M. Wheeler, Adrienne M. Stilp, Shuquan Rao, Bjarni V. Halldórsson, Doruk Beyter, Jia Wen, Anna V. Mihkaylova, Caitlin P. McHugh, John Lane, Min-Zhi Jiang, Laura M. Raffield, Goo Jun, Fritz J. Sedlazeck, Ginger Metcalf, Yao Yao, Joshua B. Bis, Nathalie Chami, Paul S. de Vries, Pinkal Desai, James S. Floyd, Yan Gao, Kai Kammers, Wonji Kim, Jee-Young Moon, Aakrosh Ratan, Lisa R. Yanek, Laura Almasy, Lewis C. Becker, John Blangero, Michael H. Cho, Joanne E. Curran, Myriam Fornage, Robert C. Kaplan, Joshua P. Lewis, Ruth J. F. Loos, Braxton D. Mitchell, Alanna C. Morrison, Michael Preuss, Bruce M. Psaty, Stephen S. Rich, Jerome I. Rotter, Hua Tang, Russell P. Tracy, Eric Boerwinkle, Goncalo R. Abecasis, Thomas W. Blackwell, Albert V. Smith, Andrew D. Johnson, Rasika A. Mathias, Deborah A. Nickerson, Matthew P. Conomos, Yun Li, Unnur Þorsteinsdóttir, Magnús K. Magnússon, Kari Stefansson, Nathan D. Pankratz, Daniel E. Bauer, Paul L. Auer, Alex P. Reiner

**Affiliations:** 1 Department of Genome Sciences, University of Washington, Seattle, WA 98105 USA; 2 Department of Biostatistics, University of Washington, Seattle, WA 98105 USA; 3 Division of Hematology/Oncology, Boston Children’s Hospital, Boston, MA 02115 USA; 4 Department of Pediatric Oncology, Dana-Farber Cancer Institute, Boston, MA 02115 USA; 5 Harvard Stem Cell Institute, Boston, MA 02138 USA; 6 Broad Institute, Cambridge, MA 02142 USA; 7 Department of Pediatrics, Harvard Medical School, Boston, MA 02115 USA; 8 State Key Laboratory of Experimental Hematology, National Clinical Research Center for Blood Diseases, Haihe Laboratory of Cell Ecosystem, Institute of Hematology & Blood Diseases Hospital, Chinese Academy of Medical Sciences & Peking Union Medical College, Tianjin, 300020 China; 9 deCODE genetics/Amgen Inc., Reykjavik, Iceland; 10 School of Technology, Reykjavik University, Reykjavík, Iceland; 11 Departments of Biostatistics, Genetics, Computer Science, Applied Physical Sciences, University of North Carolina at Chapel Hill, Chapel Hill, NC 27599 USA; 12 Department of Laboratory Medicine and Pathology, University of Minnesota Medical School, Minneapolis, MN 55455 USA; 13 Department of Genetics, University of North Carolina, Chapel Hill, NC 27599 USA; 14 Human Genetics Center, School of Public Health, The University of Texas Health Science Center at Houston, Houston, TX 77030 USA; 15 Human Genome Sequencing Center, Baylor College of Medicine, Houston, TX USA; 16 Cardiovascular Health Research Unit, Department of Medicine, University of Washington, Seattle, WA 98101 USA; 17 The Charles Bronfman Institute for Personalized Medicine, Icahn School of Medicine at Mount Sinai, New York, NY 10029 USA; 18 Department of Epidemiology, Human Genetics, and Environmental Sciences, School of Public Health, The University of Texas Health Science Center at Houston, Houston, TX 77030 USA; 19 Division of Hematology and Oncology, Weill Cornell Medical College, New York, NY 10065 USA; 20 Jackson Heart Study, Department of Medicine, University of Mississippi, Jackson, MS 39216 USA; 21 GeneSTAR Research Program, Department of Medicine, Johns Hopkins University School of Medicine, Baltimore, MD 21287 USA; 22 Channing Division of Network Medicine, Brigham and Women’s Hospital, Boston, MA 2115 USA; 23 Department of Epidemiology and Population Health, Albert Einstein College of Medicine, Bronx, NY 10461 USA; 24 Center for Public Health Genomics, University of Virginia, Charlottesville, VA 22908 USA; 25 Children’s Hospital of Philadelphia and University of Pennsylvania School of Medicine, Philadelphia, PA 19104 USA; 26 Department of Human Genetics and South Texas Diabetes and Obesity Institute, University of Texas Rio Grande Valley School of Medicine, Brownsville, TX 78520 USA; 27 Brown Foundation Institute of Molecular Medicine, McGovern Medical School, University of Texas Health Science Center at Houston, Houston, TX 77030 USA; 28 Department of Medicine, Division of Endocrinology, Diabetes, and Nutrition, University of Maryland School of Medicine, Baltimore, MD USA; 29 Department of Environmental Medicine and Public Health, Icahn School of Medicine at Mount Sinai, New York, NY USA; 30 The Mindich Child Health and Development Institute, Icahn School of Medicine at Mount Sinai, New York, NY USA; 31 Novo Nordisk Foundation Center for Basic Metabolic Research, Faculty of Health and Medical Sciences, University of Copenhagen, Copenhagen, Denmark; 32 The Institute for Translational Genomics and Population Sciences, Department of Pediatrics, The Lundquist Institute for Biomedical Innovation at Harbor-UCLA Medical Center, Torrance, CA 90502 USA; 33 Department of Genetics, Stanford University School of Medicine, Stanford, CA 94305 USA; 34 Departments of Pathology & Laboratory Medicine and Biochemistry, Larner College of Medicine at the University of Vermont, Colchester, VT 5446 USA; 35 TOPMed Informatics Research Center, University of Michigan, Department of Biostatistics, Ann Arbor, MI 48109 USA; 36 Population Sciences Branch, Division of Intramural Research, National Heart, Lung and Blood Institute, Framingham, MA 1702 USA; 37 Faculty of Medicine, University of Iceland, 101 Reykjavik, Iceland; 38 Division of Biostatistics, Institute for Health and Equity, and Cancer Center, Medical College of Wisconsin, Milwaukee, WI 53226 USA; 39 Department of Epidemiology, University of Washington, Seattle, WA 98105 USA

**Keywords:** Genome-wide association studies, DNA sequencing, Biomarkers

## Abstract

Genome-wide association studies have identified thousands of single nucleotide variants and small indels that contribute to variation in hematologic traits. While structural variants are known to cause rare blood or hematopoietic disorders, the genome-wide contribution of structural variants to quantitative blood cell trait variation is unknown. Here we utilized whole genome sequencing data in ancestrally diverse participants of the NHLBI Trans Omics for Precision Medicine program (*N* = 50,675) to detect structural variants associated with hematologic traits. Using single variant tests, we assessed the association of common and rare structural variants with red cell-, white cell-, and platelet-related quantitative traits and observed 21 independent signals (12 common and 9 rare) reaching genome-wide significance. The majority of these associations (*N* = 18) replicated in independent datasets. In genome-editing experiments, we provide evidence that a deletion associated with lower monocyte counts leads to disruption of an *S1PR3* monocyte enhancer and decreased *S1PR3* expression.

## Abstract (web summary)

Most genetic association studies have been done on single nucleotide polymorphisms and small indels, while other types of variants have been less studied. Here, the authors use whole genome sequencing in a diverse population to identify and provide experimental evidence for associations between structural variants and blood-cell traits.

## Introduction

Structural variants (SVs) are an important, yet under-studied type of human genetic variation. Numerous studies have implicated SVs (defined as > ~50 bp) with human diseases as well as normal phenotypic variation^1–5^. Common SVs (MAF > 1%) are enriched among loci identified in genome-wide association studies (GWAS)^6^. In non-coding regions, SVs have a greater impact on gene expression compared to single nucleotide variants (SNVs) and small insertions and deletions (indels)^7^. However, the discovery and genotyping of SVs is challenging and has lagged behind that of SNVs and indels. Many SVs are located within repetitive regions of the genome and often have complex structures including multiallelic copy number or repeat expansion, deletions with multiple breakpoints, or repeated rearrangement or complex inversions^6^. As a result, the contribution of SVs to the genetic architecture of complex traits remains poorly characterized.

The recent application of ensemble detection methods to whole genome sequencing (WGS) projects, particularly to large, multi-ancestry datasets, provides an opportunity to characterize the contribution of common and rare SVs to complex traits. Towards this end, we have utilized SVs detected from high-coverage WGS data from the NHLBI Trans-Omics for Precision Medicine (TOPMed) program and characterized their relationship to quantitative blood cell traits.

Red blood cell (RBC), white blood cell (WBC), and platelet laboratory parameters are routinely measured in clinical laboratories and used for monitoring general health status and diagnosis of acquired and inherited blood-related disorders. In the general population, hematologic quantitative traits are highly heritable and serve as a model system for studying the genetic architecture of complex traits^8^. Thus far, hundreds of genomic loci and thousands of genetic variants have been associated with hematologic traits; however, these variants are almost exclusively SNVs and indels^9,10^. For a few GWAS loci, there is evidence that common SVs are likely the causal variant responsible for the phenotypic effects in the population at large. For example, a common 3.7 kb alpha-globin gene deletion largely accounts for the strong association signal between RBC phenotypes and the 16p13.3 locus in African ancestry populations^11–13^. While private SVs have been identified in individuals with rare Mendelian blood disorders (for example, a rare *PLAU* 78 kb tandem duplication responsible for autosomal dominant Quebec platelet disorder^14^), the contribution of rare SVs (MAF < 1%) to quantitative hematologic traits among unselected individuals has not been assessed.

In up to 50,675 ancestrally diverse TOPMed participants, we assessed the association of common and rare SVs (deletions, duplications, and inversions) with variation in RBC, WBC, and platelet-related quantitative traits. We characterized linkage disequilibrium patterns and performed conditional regression analyses that included SNVs/indels previously associated with the same hematologic trait. Additionally, we used gene editing in monocytic and primary human hematopoietic stem and progenitor cells (HSPCs) followed by xenotransplantation to demonstrate the mechanism by which a newly detected deletion disrupts an *S1PR3* monocyte enhancer and leads to decreased *S1PR3* expression and lower monocyte count.

## Results

### Identification of common and rare SVs associated with blood cell traits

We performed single variant association tests, across 24 quantitative hematologic traits in up to 50,675 multi-ancestry TOPMed participants (Supplementary Data 1). Single variant association tests were performed for SVs with a minor allele count (MAC) ≥ 5 (number of SVs=96,049). SVs in TOPMed were detected and genotyped from WGS using the Parliament2 pipeline^15^ and muCNV genotyper^16^ (see Methods). The QQ plots and genomic inflation factors (ranging from 0.981 to 1.056) were well-calibrated indicating good control of population stratification and relatedness (Fig. S1). Further stratification of the QQ plots by allele frequency showed no evidence of inflation even for minor allele counts in the 5–10 range **(**Fig. S2). Across the 24 hematologic traits, a total of 21 independent SVs (deletions=14, duplications=6, and inversions=1) or 41 SV-trait associations were genome-wide significant (Table 1 and Fig. S3).

**Table 1:** Summary of genome-wide structural variants associated with hematological trait

					TOPMed Allele Frequencies					Significant single variant association tests			
Gene(s)	Locus	SV Breakpoints¹	SV type²	Length (bp)	Overall	African³	Asian³	Hispanic³	European³	Significant Trait	Effect Estimate	SE	*P*-value
-	2p11.2^4^	2:88832769-88860930	DEL	28162	1.98E-04	4.06E-05	0	0	2.98E-04	Lymphocytes	1.878	0.310	1.36E-09
										WBC	2.813	0.426	3.84E-11
RAB7A	3q21.3	3:128694260-128695207	DUP	948	0.085	0.022	0.031	0.065	0.119	Monocyte prop.	−0.002	0.0004	6.91E-09
SRPRB, TF, TOPBP1	3q22.1	3:133621201-133784900	DUP	163700	0.208	0.141	0.308	0.200	0.236	TIBC	20.809	2.209	4.56E-21
										UIBC	19.298	2.553	4.09E-14
C3orf36, LINC02000, RAB6B, SLCO2A1, SRPRB, TF	3q22.1-q22.2	3:133786201-134102300	DUP	316100	0.272	0.207	0.358	0.267	0.292	TIBC	17.804	2.324	1.84E-14
										UIBC	19.463	2.687	4.35E-13
PSORS1C1	6p21.33	6:31132409-31132465	DEL	57	0.085	0.049	0.075	0.103	0.094	Lymphocytes	0.075	0.012	2.01E-09
-	6p21.32	6:32591559-32591660	DEL	102	0.264	0.167	0.281	0.293	0.293	WBC	0.092	0.015	1.25E-09
-	6p21.1	6:41897089-41897626	DEL	538	0.183	0.079	0.209	0.151	0.235	MCH	−0.187	0.022	1.28E-17
										MCV	−0.595	0.055	3.04E-27
										RBC	0.033	0.005	1.57E-12
CCND3	6p21.1	6:41985574-41988887	DEL	3314	0.007	0.002	0.001	0.007	0.010	MCV	1.556	0.256	1.16E-09
-	6p23.3	6:134878573-134878641	DUP	69	0.044	0.072	0.030	0.037	0.036	MCH	−0.254	0.046	4.23E-08
-	9q22.1	9:88923551-88924152	DEL	602	0.034	0.117	7.08E-04	0.032	7.31E-04	Monocytes	−0.029	0.004	2.20E-11
										Monocyte prop.	−0.004	−0.004	1.07E-12
ATXN2	12q24.12	12:111538485-111542205	DEL	3721	0.068	0.016	0.021	0.058	0.092	Platelets	−6.063	0.820	1.40E-13
ATXN2	12q24.12	12:111542097-111542205	INV	109	0.062	0.015	0.021	0.054	0.085	Platelets	−6.371	0.911	2.65E-12
-	14q32.33^4^	14:105863184-105897962	DEL	34779	2.24E-04	4.16E-05	0	0	3.43E-04	Lymphocytes	2.037	0.256	1.87E-15
										Lymphocyte prop.	0.228	0.030	2.49E-14
										Neutrophil prop.	−0.222	0.034	1.08E-10
-	14q32.33^4^	14:105864247-105902650	DEL	38404	7.96E-05	0	0	0	1.41E-04	WBC	3.115	0.483	1.15E-10
HBA1, HBA2	16p13.3	16:165396-184701	DEL	19306	5.95E-05	0	0.004	6.32E-05	0	MCH	−6.570	0.865	3.12E-14
										MCV	−16.599	2.305	5.97E-13
										RBC	1.437	0.189	3.02E-14
HBA1, HBA2	16p13.3	16:172001-177200	DEL	5200	0.057	0.176	0.030	0.060	0.008	Hematocrit	−0.485	0.054	2.65E-19
										Hemoglobin	−0.437	0.018	5.06E-127
										MCH	−2.668	0.038	<5E-324
										MCHC	−0.701	0.015	<5E-324
										MCV	−6.276	0.099	<5E-324
										RBC	0.296	0.008	<5E-324
										RBW	0.545	0.030	1.19E-73
										MPV	0.118	0.021	1.60E-08
FAM234A	16p13.3	16:246437-249971	DEL	3535	0.006	0.019	7.39E-04	0.005	7.54E-05	MCH	−0.788	0.120	4.90E-11
										MCV	−1.917	0.310	6.07E-10
CAPN15	16p13.3	16:550075-550141	DEL	67	0.025	0.116	7.05E-04	0.020	3.39E-04	MCH	−0.374	0.063	2.92E-09
										MCV	−0.929	0.162	8.74E-09
KCNJ18	17p11.2	17:21659501-21795800	DUP	136300	0.511	0.519	0.509	0.508	0.508	Lymphocyte prop.	0.022	0.004	4.36E-10
										Neutrophils prop.	−0.025	0.004	3.90E-10
-	18p11.22	18:9621931-9622237	DEL	307	0.791	0.839	0.921	0.766	0.770	MPV	−0.079	0.014	9.32E-09
TMPRSS6	22q12.3	22:37067818-37067888	DUP	71	0.347	0.483	0.323	0.324	0.301	Hemoglobin	0.059	0.010	1.62E-08

¹Structural variant breakpoints predicted by the Parliament2 pipeline.

²Types of structural variant identified: deletions (DEL), duplications (DUP), inversions (INV).

³Discrete ancestry subgroups are based on genetically inferred ancestry and a machine learning algorithm that refines self-identified ancestry.

^4^Based on location and WGS read visualization SV likely represents a somatic deletion or a complex rearrangement due to V(D)J recombination events.

All *p*-values are derived from two-sided t-tests and are not adjusted for multiple comparisons. *WBC* White Blood Cells, *TIBC* Total iron binding capacity, *UIBC* Unsaturated iron binding capacity, *MCH* Mean corpuscular hemoglobin, *MCV* Mean corpuscular volume, *RBC* Red blood cells, *RBW* Red cell width, *MCHC* Mean corpuscular hemoglobin concentration, *MPV* Mean Platelet Volume.

The 21 trait-associated SVs ranged in size from ~60 bp to >160 kb (Table 1) and exhibited a range of allele frequencies: 12 are common (overall TOPMed MAF > 1%) and 9 are rare (ranging from 0.006% to 0.7% MAF in TOPMed) with a few significant SVs exhibiting allele frequencies differences across populations (Table 1). For instance, the monocyte-associated deletion on chromosome 9q22.1 and a subset of the 16p13.3 red cell trait-associated SVs are more common in individuals of African ancestry than in individuals of non-African ancestry.

### Replication of significant SV-blood cell trait associations

We attempted replication for each of the 21 trait-associated SVs using a combination of short-read and long-read WGS data and genotype imputation. We utilized independent datasets composed of Icelandic (deCODE genetics)^17–19^ and multi-ancestry (UK Biobank, UKBB)^20^ participants. Note that the SV calling and genotyping algorithms used in replication datasets (described under Methods) are different from the Parliament2 pipeline used for SV discovery in TOPMed. To account for these methodological differences, we determined a set of “representative SVs” in deCODE genetics and UKBB datasets. We defined an SV in a replication dataset as “representative” if the SV was located within 5 kb of the trait-associated TOPMed SV and if the two SV sizes overlapped by at least 25%. In addition, we considered SVs as representative if they were the same structural variant type (e.g. both SVs were deletions) and had similar minor allele frequencies in the relevant population.

Using these criteria, 3 of the 21 trait-associated SVs did not have a SV representative in deCODE or UKBB datasets, including SVs at 2q11.2 (2:88832769-88860930), 3q22.1 (3:133621201-133784900), and 17p11.2 (17:21659501-21795800) (Supplementary Data 2). A total of 18 trait-associated SVs did have a representative and all of these were robustly replicated for the same blood cell trait (i.e., with a *p*-value < 0.05/number of its representative SVs in deCODE Icelandic, UKBB British, UKBB African, or UKBB South Asian cohorts and consistent direction of the effect) (Supplementary Data 2).

### Trait-associated SVs in regions of LD with known GWAS loci

To determine if trait-associated SVs discovered in TOPMed are independent of previously reported GWAS SNVs/indels^9,10,21–23^, we calculated pairwise linkage disequilibrium (LD) between TOPMed SVs and TOPMed SNVs/indels (Table 2, Fig. S4). We also performed two sets of conditional analyses (see Methods). LD analysis shows 16 of 21 trait-associated SVs are in at least moderate LD (r2 ≥ 0.75) with one SNV/indel previously associated with the same hematologic trait (Table 2, Fig. S4). These include 7 SVs with at least one trait-associated SNV/indel in near perfect LD (r^2^ ≥ 0.99). Conditional regression analysis confirmed that 16 SV association signals were not significant following adjustment for known SNV/indels at the same trait loci (Table 3), supporting the non-independence between SV and SNV/indel associations at these loci.

**Table 2:** Single nucleotide variants (SNVs) and small insertions and deletions (indels) in linkage disequilibrium (r2 ≥ 0.75) with structural variants associated with hematological traits

Gene(s)	Locus	SV Breakpoints¹	SV type²	SV allele frequency	SNV/indel; ref>alt	SNV, indel allele frequency	r2	SNV/indel *P*-value³	SNV/indel Effect Estimate³	SNV/indel Effect Estimate SE³
*-*	2p11.2	2:88832769-88860930	DEL	1.98E-04	-	-	-	-	-	-
*RAB7A*	3q21.3	3:128694260-128695207	DUP	0.085	3:128694296; A > T	0.08	0.781	5.60E-08	−0.002	0.0004
*SRPRB, TF, TOPBP1*	3q22.1	3:133621201-133784900	DUP	0.208	3:133789620; A > T	0.295	0.819	3.70E-32	17.697	1.499
*C3orf36, LINC02000, RAB6B, SLCO2A1, SRPRB, TF*	3q22.1-q22.2	3:133786201-134102300	DUP	0.272	3:133789620; A > T	0.295	0.791	3.70E-32	17.697	1.499
*PSORS1C1*	6p21.33	6:31132409-31132465	DEL	0.085	6:31133806; G/ > A	0.084	0.99	3.15E-06	0.109	0.023
*-*	6p21.32	6:32591559-32591660	DEL	0.264	6:32593469; T > C	0.277	0.999	1.23E-08	0.084	0.015
*-*	6p21.1	6:41897089-41897626	DEL	0.183	6:41880062: G > A	0.186	1	3.74E-18	−0.18	0.021
*CCND3*	6p21.1	6:41985574-41988887	DEL	0.007	6:41984773; T > G	0.007	0.999	4.67E-10	0.613	0.098
*-*	6p23.3	6:134878573-134878641	DUP	0.044	6:134870213; A > G	0.107	0.79	7.16E-12	−0.189	0.028
*-*	9q22.1	9:88923551-88924152	DEL	0.034	9:88921159; C > A	0.04	0.996	1.03E-11	−0.029	0.004
*ATXN2*	12q24.12	12:111538485-111542205	DEL	0.068	12:111599646; G, > A	0.058	0.991	7.81E-14	−6.142	0.822
*ATXN2*	12q24.12	12:111542097-111542205	INV	0.062	12:111599646; G > A	0.058	0.936	7.81E-14	−6.142	0.822
*-*	14q32.33	14:105863184-105897962	DEL	2.24E-04	-	-	-	-	-	-
*-*	14q32.33	14:105864247-105902650	DEL	7.96E-05	-	-	-	-	-	-
*HBA1, HBQ1, HBA2*	16p13.3	16:165396-184701	DEL	5.95E-05	16:199621; AG > A	0.0004	0.848	7.16E-13	−5.894	0.821
*HBA1, HBA2, HBQ1*	16p13.3	16:172001-177200	DEL	0.057	-	-	-	-	-	-
*FAM234A*	16p13.3	16:246437-249971	DEL	0.006	16:244752; G, > A	0.007	0.983	2.39E-11	−0.764	0.114
*CAPN15*	16p13.3	16:550075-550141	DEL	0.025	16:550141; T > C	0.089	0.857	1.08E-20	−0.333	0.036
*KCNJ18*	17p11.2	17:21659501-21795800	DUP	0.511	-	-	-	-	-	-
*-*	18p11.22	18:9621931-9622237	DEL	0.791	18:9621307; T > G	0.781	0.998	1.29E-07	−0.068	0.013
*TMPRSS6*	22q12.3	22:37067818-37067888	DUP	0.347	22:37071230; C > T	0.438	0.774	1.47E-13	0.125	0.017

¹Structural variant breakpoints predicted by the Parliament2 pipeline.

²Types of structural variant identified: deletions (DEL), duplications (DUP), inversions (INV).

³Single variant association test statistics performed for SNV, indels, performed using the same sample set used for SV analyses and for the same hematological trait(s).

All *p*-values are derived from two-sided t-tests and are not adjusted for multiple comparisons.

**Table 3:** Structural variants conditioned on single nucleotide variants/indels from previous genome-wide association studies

						Marginal			Conditioned on TOPMed¹			Conditioned on BCX²		
Gene(s)	Locus	SV Breakpoints	SV type	TOPMed Allele Frequency	Significant Trait	Effect Estimate	Effect Estimate SE	*P*-value	Effect Estimate	Effect Estimate SE	*P*-value	Effect Estimate	Effect Estimate SE	*P*-value
-	2p11.2^3^	2:88832769-88860930	DEL	1.98E-04	Lymphocytes	1.878	0.31	1.36E-09	1.742	0.288	1.49E-09	2.087	0.309	1.42E-11
					WBC	2.813	0.426	3.84E-11	2.929	0.42	2.94E-12	3.061	0.419	2.67E-13
*RAB7A*	3q21.3	3:128694260-128695207	DUP	0.085	Monocyte prop.	−0.002	0.0004	6.91E-09	−0.001	3.93E-04	0.181	−7.31E-04	4.18E-04	0.08
*SRPRB, TF, TOPBP1*	3q22.1	3:133621201-133784900	DUP	0.208	TIBC	20.809	2.209	4.56E-21	−0.751	2.145	0.726	-	-	-
					UIBC	19.298	2.553	4.09E-14	−1.899	3.537	0.591	-	-	-
*C3orf36, LINC02000, RAB6B, SLCO2A1, SRPRB, TF*	3q22.1-q22.2	3:133786201-134102300	DUP	0.272	TIBC	17.804	2.324	1.84E-14	−1.179	1.972	0.55	-	-	-
					UIBC	19.463	2.687	4.35E-13	1.182	3.387	0.727	-	-	-
*PSORS1C1*	6p21.33	6:31132409-31132465	DEL	0.085	Lymphocytes	0.075	0.012	2.01E-09	0.023	0.029	0.426	0.047	0.014	9.48E-04
-	6p21.32	6:32591559-32591660	DEL	0.264	WBC	0.092	0.015	1.25E-09	0.146	0.061	0.016	0.068	0.016	1.20E-05
-	6p21.1	6:41897089-41897626	DEL	0.183	MCH	−0.187	0.022	1.28E-17	−0.075	0.047	0.108	−0.12	0.046	0.009
					MCV	−0.595	0.055	3.04E-27	−0.249	0.119	0.037	−0.267	0.087	0.002
					RBC	0.033	0.005	1.57E-12	0.012	0.012	0.313	0.024	0.01	0.018
*CCND3*	6p21.1	6:41985574-41988887	DEL	7.00E-03	MCV	1.556	0.256	1.16E-09	−0.994	0.845	0.24	−1.597	0.878	0.069
-	6p23.3	6:134878573-134878641	DUP	4.40E-02	MCH	−0.254	0.046	4.23E-08	−0.039	0.052	0.449	−0.093	0.045	0.042
-	9q22.1	9:88923551-88924152	DEL	0.034	Monocytes	−0.029	0.004	2.20E-11	0.014	0.029	0.642	−0.032	0.004	4.64E-13
					Monocyte prop.	−0.004	0.001	1.07E-12	0.005	0.004	0.222	−0.005	5.89E-04	2.61E-16
*ATXN2*	12q24.12	12:111538485-111542205	DEL	0.068	Platelets	−6.063	0.82	1.40E-13	8.886	7.878	0.259	−1.595	1.046	0.127
*ATXN2*	12q24.12	12:111542097-111542205	INV	0.062	Platelets	−6.371	0.911	2.65E-12	0.385	2.423	0.874	−1.656	1.108	0.135
-	14q32.33^3^	14:105863184-105897962	DEL	2.24E-04	Lymphocytes	2.037	0.256	1.87E-15	2.76	0.258	1.13E-26	2.248	0.254	9.36E-19
					Lymphocyte prop.	0.228	0.030	2.49E-14	0.203	0.028	2.31E-13	0.208	0.027	1.71E-14
					Neutrophils prop.	−0.222	0.034	1.08E-10	−0.207	0.033	6.33E-10	−0.212	0.033	1.52E-10
-	14q32.33^3^	14:105864247-105902650	DEL	7.96E-05	WBC	3.115	0.483	1.15E-10	3.144	0.471	2.51E-11	3.229	0.472	7.52E-12
*HBA1, HBQ1, HBA2*	16p13.3	16:165396-184701	DEL	5.95E-05	MCH	−6.57	0.865	3.12E-14	−0.782	2.588	0.762	−6.053	0.808	6.66E-14
					MCV	−16.599	2.305	5.97E-13	−2.362	6.497	0.716	−15.189	2.151	1.64E-12
					RBC	1.437	0.189	3.02E-14	0.622	0.581	0.284	1.359	0.185	1.99E-13
*HBA1, HBA2, HBQ1*	16p13.3	16:172001-177200	DEL	0.057	Hematocrit	−0.485	0.054	2.65E-19	−0.467	0.053	1.50E-18	−0.492	0.054	5.05E-20
					Hemoglobin	−0.437	0.018	5.06E-127	−0.44	0.025	4.87E-71	−0.449	0.021	8.19E-104
					MCH	−2.668	0.038	<5E-324	−2.621	0.06	<5E-324	−2.704	0.041	<5E-324
					MCHC	−0.701	0.015	<5E-324	−0.652	0.025	5.87E-156	−0.708	0.016	<5E-324
					MCV	−6.276	0.099	<5E-324	−6.22	0.153	<5E-324	−6.483	0.111	<5E-324
					RBC	0.296	0.008	<5E-324	0.267	0.012	9.77E-109	0.298	0.009	1.58E-245
					RBW	0.545	0.03	1.19E-73	0.509	0.036	1.79E-46	0.566	0.032	3.52E-70
					MPV	0.118	0.021	1.60E-08	0.119	0.019	6.23E-10	0.117	0.019	5.99E-10
*FAM234A*	16p13.3	16:246437-249971	DEL	0.006	MCH	−0.788	0.12	4.90E-11	−0.401	0.264	0.129	−0.86	0.116	1.48E-13
					MCV	−1.917	0.31	6.07E-10	−0.502	0.686	0.464	−1.958	0.298	5.41E-11
*CAPN15*	16p13.3	16:550075-550141	DEL	0.025	MCH	−0.374	0.063	2.92E-09	−0.036	0.072	0.611	−0.412	0.061	1.21E-11
					MCV	−0.929	0.162	8.74E-09	−0.27	0.185	0.144	−0.618	0.155	6.86E-05
*KCNJ18*	17p11.2	17:21659501-21795800	DUP	0.511	Lymphocyte prop.	0.022	0.004	4.36E-10	0.023	0.003	2.17E-11	0.023	0.003	4.07E-11
					Neutrophils prop.	−0.025	0.004	3.90E-10	−0.024	0.004	1.56E-09	−0.023	0.004	2.57E-09
-	18p11.22	18:9621931-9622237	DEL	0.791	MPV	−0.079	0.014	9.32E-09	−0.043	0.031	0.167	−0.065	0.03	0.031
*TMPRSS6*	22q12.3	22:37067818-37067888	DUP	0.347	Hemoglobin	0.059	0.01	1.62E-08	−0.007	0.016	0.669	−0.006	0.015	0.685

¹Analyses included previously-reported SNVs or small indels with *P* < 5e-8 in TOPMed single variant association tests (Mikhaylova et al. 2021; Hu et al. 2021).

²Analysis included TOPMed SNVs and small indels that matched variants reported in Chen et al. 2020 and Vuckovic et al. 2020 as part of the Blood Cell Consortium (BCX).

^3^Based on location and WGS read visualization SV likely represents a somatic deletion or a complex rearrangement due to V(D)J recombination events. Conditional analyses were performed for each blood trait. Matched variants on each chromosome were LD-pruned and included as fixed effects in a two-stage LMM association testing. All p-values are derived from two-sided t-tests and are not adjusted for multiple comparisons. *WBC* White Blood Cells, *TIBC* Total iron binding capacity, *UIBC* Unsaturated iron binding capacity, *MCH* Mean corpuscular hemoglobin, *MCV* Mean corpuscular volume, *RBC* Red blood cells, *RBW* Red cell width, *MCHC* Mean corpuscular hemoglobin concentration, *MPV* Mean Platelet Volume.

### Conditional analyses of trait-associated SVs adjusting for known GWAS SNVs/indels

A total of 5 trait-associated SVs remained genome-wide significant following conditional analyses (Table 3). This result suggests that these association SV signals may be causally distinct and that the previously identified association with an SNV/indel was reported due to LD with the unmeasured causal SV. These 5 SVs span 4 genomic loci. We discuss these genomic loci in greater detail below.

#### 16p13.3 (alpha-globin) locus

The strongest association signal in our analyses was located at the 16p13.3 locus where a deletion spanning *HBA1/HBA2* (16:172001-177200) was associated with all 7 red cell traits (Table 1, Fig. S5). LD and conditional analyses indicate this deletion (16:172001-177200) is independent of other known red cell trait-associated SNVs/indels (Tables 2, 3). Although Parliament2 predicted this deletion as being 5.2 kb in size (see Table 1), this deletion represents a previously characterized 3.7 kb alpha-globin deletion^11^. This was confirmed by WGS read visualization in samples predicted to exhibit the *HBA1/HBA2* (16:172001-177200) deletion. Visualization shows SV breakpoints predicted by Parliament2 for this event are inaccurate and span the previously characterized 3.7 kb deletion (see example in Fig. S5). The 3.7 kb alpha-globin deletion is known to be more common in African ancestry individuals^21^. In our study, the overall allele frequency of the *HBA1/HBA2* deletion (16:172001-177200) was 5.7% and 17.6% in the African ancestry sub-population.

SV analyses also found the *HBA1/HBA2* deletion (16:172001-177200) as significantly associated with higher mean platelet volume (MPV) (Table 1). This was unexpected as none of the alpha-globin genes are known to regulate megakaryocyte or platelet production. While transcripts of genes located within the alpha-globin cluster on 16p13.3 are detectable in iPSC-induced megakaryocytes^24^, we observed no evidence that this deletion is a *cis*-eQTL among African-ancestry individuals from the TOPMed GeneSTAR cohort (Bonferroni-corrected *P*-values > 0.15 for all genes within a 1 Mb window). These observations are based on evidence from analysis of RNA from platelets (*n* = 110) and iPSC-induced megakaryocytes (*n* = 84). Relatedly, we found no association between the alpha-globin deletion (16:172001-177200) and circulating platelet counts in TOPMed (*P* = 0.75). Based on these observations, along with the lack of any apparent association of the 16:172001-177200 deletion with circulating platelet count in TOPMed (*P* = 0.75), we hypothesize that the association with platelet size likely represents a laboratory artifact in which very small (microcytic) RBCs are being counted as “large platelets” thereby resulting in an apparent increase in MPV.

In addition to the *HBA1/HBA2* deletion (16:172001-177200), analyses identified 3 other 16p13.3 deletions located within 500 kb of the alpha-globin gene cluster. LD and conditional analyses suggest these 3 deletions are not independent from trait-associated SNVs/indels in this region (Tables 2, 3). However, all 3 deletions showed a similar “thalassemia-like” pattern of red cell phenotypic association (lower MCH and MCV and higher RBC count) (Table 1)^11^. These deletions range in size from ~70 bp to 19,000 bp. The ~19 kb deletion (allele frequency 0.006% in TOPMed overall and 0.4% in TOPMed Asian ancestry individuals) impacts both *HBA1* and *HBA2* and likely corresponds to the well-characterized alpha-thalassemia variant known as –(SEA)^11^. The two other red cell trait-associated SVs on 16p13.3 are located ~70 to ~400 kb downstream of the alpha-globin genes and are not predicted to alter regions involved in alpha-globin gene regulation or show evidence by promoter Hi-C capture of physical interaction with globin gene promoters in blood cells (Supplementary Data 3).

#### 17p11.2 (KCNJ18) locus

A complex, multiallelic SV near the centromere of chromosome 17 (17p11.2) was significantly associated with higher lymphocyte proportions and lower neutrophil proportions (Table 1, Fig. S5). This SV is predicted by Parliament2 to be a large duplication that includes the *KCNJ18* gene. Of note, the genomic region containing *KCNJ18* is not present in GRCh37; thus, this region was not interrogated in prior GRCh37 blood cell trait GWAS. In GRCh38, there is one copy of *KCNJ18*; however, based on Parliament2 SV calls, this region is likely duplicated (diploid copy number = 4) in most individuals (~87% of individuals in our TOPMed dataset). A subset of individuals (~2.7%) are estimated to have more than 4 diploid copies.

There is no LD between the *KCNJ18* SV and SNV/indels in the region (Table 2) and the SV-trait association is independent of known GWAS variants (Table 3). These results are consistent with a recent TOPMed WGS-based analysis, where no SNVs/indels in the 17p11.2 region were associated with WBC, neutrophil, or lymphocyte traits^22^. However, given the phenotypic pattern (opposing effects on neutrophil and lymphocyte proportions) associated with the *KCNJ18* duplication, the complexity of the locus, the absence of a known role of *KCNJ18* in leukocyte biology, and the lack of detection in our replication cohorts (see above) additional work is needed to substantiate these results.

#### 2p11.2 and 14q32.33 immunoglobulin gene regions

Complex SVs at two loci, 2p11.2 and 14q32.33, were significantly associated with lymphocyte, neutrophil and WBC traits (Table 1, Fig. S5). These SV associations remained significant following adjustment for known WBC trait-associated SNVs/indels (Table 3). SVs at both of these loci are rare and relatively large in size (Table 1). They are predicted to impact immunoglobulin kappa (2p11.2) and heavy chain (14q32.33) gene clusters. Based on their location and on visualization of WGS reads, these SVs likely represent somatic deletions and/or complex rearrangements due to V(D)J recombination events related to B cell maturation or immunoglobulin production^25^.

### Proportion of TOPMed SVs tagging known hematologic trait GWAS sentinel SNV/indels

To more broadly understand the extent to which SVs tag known, blood-cell trait SNVs/indels, we calculated LD for the genotypes of previously-reported SNVs/indels^10^ and the genotypes of SVs TOPMed participants. These analyses were performed in European ancestry samples (see Methods). Approximately 3% of previously-reported blood cell trait-associated SNVs/indels^10^ (171 of the 6652) were well-tagged (r^2^ > 0.8) by a TOPMed SV. For these 171 correlated pairs, we compared the trait-association *p*-values in TOPMed in an equivalent sample set of individuals with European ancestry. For most of the SNV/indel-SV pairs, the *p*-values were within an order of magnitude of each other (Fig. S6), indicating additional functional analyses are needed to identify the causal variant.

### Functional annotation of blood cell trait-associated SVs

Functional annotation can provide additional information to prioritize causal variants at trait-associated loci. Of the 21 trait-associated SVs, 7 SVs (4 duplications and 3 deletions) are predicted to overlap coding regions and thus potentially impact protein structure/function (Supplementary Data 3). In addition to the *KCNJ18* SV described above, two duplications spanning the transferrin gene (*TF*) coding and regulatory regions were associated with higher TIBC or transferrin levels (Table 1). Two red cell phenotype-associated deletions are predicted to impact coding regions namely the deletion encompassing the known 3.7 kb alpha-globin deletion which impacts the 3’ end of *HBA2* and 5’ end of *HBA1 and* the 19 kb deletion which comprises the SEA alpha-globin deletion and impacts both alpha-globin genes as well as *HBM* and *HBQ1*.

Based on functional annotation, most trait-associated SVs (*N* = 14) are predicted to only impact non-coding/regulatory genomic regions (intronic=6, intergenic=8) (Supplementary Data 3). We cross-referenced SVs with candidate *cis* regulatory elements (cCREs) from ENCODE and several annotations relevant to 3D chromosome structure (frequently interacting regions or FIREs, topologically associating domains or TADs, super-interactive promoters or SIPs, and chromatin interactions^26–31^). Annotation results show 11 trait-associated SVs overlapped cCREs, 11 overlapped with TAD boundaries, and 3 overlapped with FIREs in relevant tissues/cell-types (i.e., GM12878 and spleen) (Supplementary Data 3). Two SVs overlapped SIPs in relevant cell-types. Chromatin interaction annotations from promoter capture Hi-C (pcHi-C) data^28^ show that across 17 blood-cell-lineage cell types, 5 SVs overlap with the promoter regions of 11 genes. This includes deletions which overlap the promoter regions for the gene *HLA-DRB1* and a duplication which overlaps the promoter region of the *TF* gene. pcHi-C data also show 10 SVs overlap regions that interact with the promoters of 83 genes (Supplementary Data 3). Similarly, monocyte Hi-C data^27^ show 17 SVs overlap potential regulatory regions interacting with promoters of 126 genes. Altogether, non-coding, functional annotations suggest most blood cell trait associated SVs may have an impact on transcriptional regulation.

### Fine-mapping and experimental validation of the 9q22.1 (*S1PR3*) monocyte locus

**Fig. 1:**
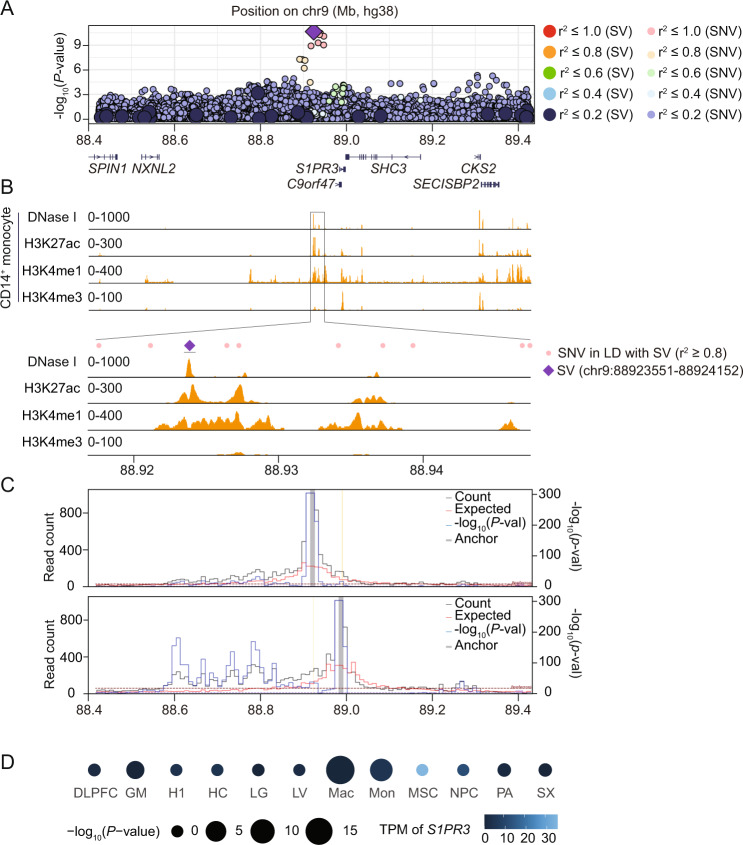
A structural variant at human 9q22.1 associated with decreased peripheral monocyte count. All *p*-values are derived from two-sided t-tests and are not adjusted for multiple comparisons. **A** Genome-wide association -log10(*p*-values) for 9q22.1 variants associated with peripheral monocyte counts. The purple diamond represents the trait-associated deletion (9:88923551-88924152); large circles represent other SVs; and small circles represent single nucleotide variants (SNVs) or indels. Color indicates the linkage disequilibrium (LD) calculated in the analysis sample set between the trait-associated deletion and individual SVs and SNVs. **B** Distribution of accessible chromatin (by DNase I sequencing) and histone modifications (H3K27ac, H3K4me1 and H3K4me3) in primary CD14 + monocytes across indicated genomic regions from ENCODE^26^. **C** Virtual 4C plot of long-range chromatin interactions anchored at the trait-associated, 9q22.1 deletion (9:88923551-88924152, upper panel) and the *S1PR3* promoter region (9:91605763-91606263, lower panel), shown as a grey bar, in macrophages. Yellow line highlights the *S1PR3* promoter region (upper panel) trait-associated, 9q22.1 deletion (lower panel). The observed and expected chromatin contact frequencies (or counts) are represented by the black and red lines, respectively. The left Y axis displays the range of chromatin contact frequency. The statistical significance (–log10(*P*-value)) of each long-range chromatin interaction is represented by the blue line, with its range listed in the right Y axis. The cell line or tissue specific FDR threshold (5%) is shown as a purple horizontal dashed line, and the more stringent Bonferroni threshold (*P* = 0.05) is shown as a maroon horizontal dashed line. **D** Long-range chromatin interaction between the trait-associated, 9q22.1 deletion and *S1PR3* promoter calculated in 12 different cell types. MSC (mesendoderm), NPC (neural progenitor cell), HC (hippocampus), H1 (human embryonic stem cells), LV (left ventricle), PA (pancreas), SX (spleen), DLPFC (dorsolateral prefrontal cortex), LG (lung)^31^, GM (lymphoblast)^76^, Mac (macrophages), Mon (monocytes)^27^. The circle size represents the magnitude of the -log10 *p*-value while the color indicates *S1PR3* mRNA level. TPM: transcripts per million.

In cases where fine-mapping and functional evidence is similar between trait-associated SVs and correlated SNVs/indels, further experimental follow-up may disentangle the causal variant. To illustrate this point, we performed experimental follow-up on a moderately sized deletion (602 bp) at the 9q22.1 locus. This deletion is near the *S1PR3* gene and was significantly associated with lower monocyte count and lower monocyte percentage (Table 1). This 9q22.1 deletion is also in near perfect LD with a recently reported monocyte-associated SNV (rs28450540) (Fig. 1A, Table 2) and several other SNVs, all of which are relatively specific to individuals of African ancestry (MAF = 0.117).

To characterize the 9q22.1 locus, we compared the overlap between monocyte count-associated variants with deoxyribonuclease I (DNase I) sensitivity, an indicator of accessible chromatin. In several cell types, such as CD34^+^ common myeloid progenitor (CMP) cells and mesenchymal stem cells (MSCs), there was a relative absence of DNase I sensitivity adjacent to or overlying the 9q22.1 locus (Fig. S7A). However, in human primary CD14 + monocytes, several peaks of DNase I hypersensitivity overlap the monocyte-associated variants (Fig. 1B). Strikingly, the trait-associated 9q22.1 deletion (9:88923551-88924152) strictly overlapped a DNase I hypersensitivity peak, suggestive of regulatory potential (Fig. 1B). None of the SNVs with r^2^ > 0.8 with the 9q22.1 deletion directly overlapped DNase I peaks (Fig. 1B). In addition, sequences at the DNase I peak overlapping the 9q22.1 deletion showed histone modifications consistent with an enhancer signature in CD14^+^ monocytes, including the presence of H3K27ac and H3K4me1 and absence of H3K4me3 marks (Fig. 1B).

A common feature of distal regulatory elements is long-range interaction with cognate promoters. We investigated these interactions from the viewpoint of the 9q22.1 SV using Hi-C data from monocytes and macrophages^27^. We observed frequent interactions between the SV-deleted sequences and the *S1PR3* promoter, which is located in the same topologically associating domain (TAD) 67.3 kb downstream (Fig. 1C). Reciprocally, we investigated interactions from the viewpoint of the *S1PR3* promoter. The interactions between the *S1PR3* promoter and the 9q22.1 SV reached genome-wide significance in macrophage Hi-C data and were just below genome-wide significance in monocyte Hi-C data (Fig. 1C and Fig. S7B). In 10 other Hi-C datasets, including from cell types that express higher levels of *S1PR3* compared to monocytes or macrophages, such as MSCs, we did not observe significant interactions between the *S1PR3* promoter and the 9q22.1 deletion (Fig. 1D). These results suggest the trait-associated 9q22.1 SV overlaps a monocyte/macrophage-specific enhancer element that interacts with *S1PR3*.

**Fig. 2:**
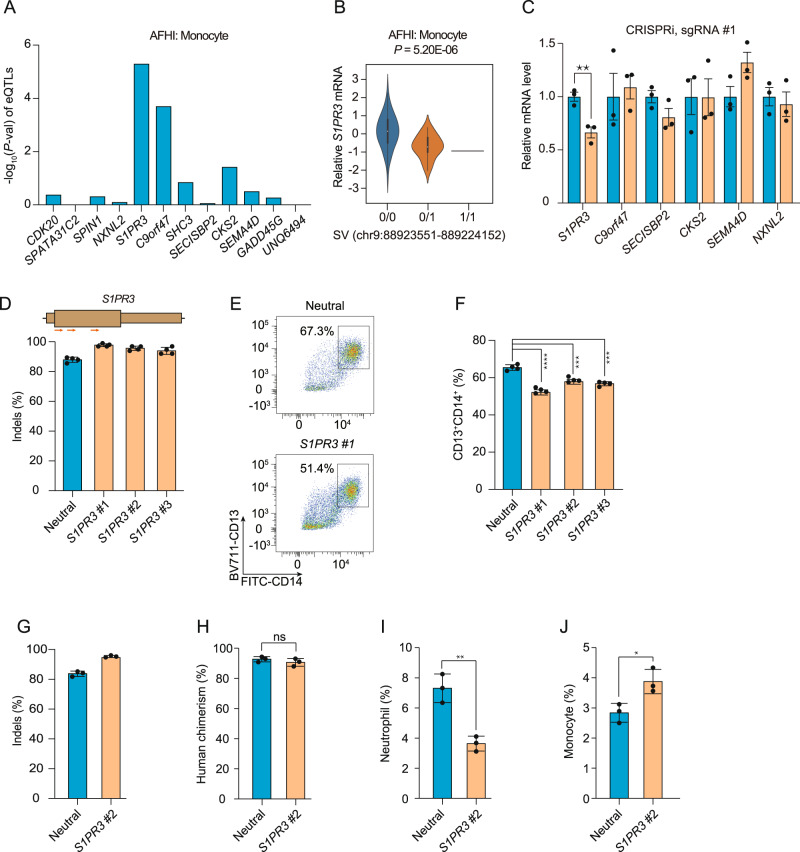
Genome and epigenome editing implicates *S1PR3* in the 9q22.1 monocyte association. All *p*-values are derived from two-sided t-tests (unless otherwise indicated) and are not adjusted for multiple comparisons. **A** eQTL results between the 9q22.1 SV and genes within 1-Mb window in monocytes using data from from MESA, including *n* = 77 AA and *n* = 92 Hispanic/Latino participants. AFHI: African American and Hispanic/Latino. **B** Violin plot (with minima, maxima, median, and inter-quartile range) demonstrating the correlation between the 9q22.1 SV genotype and expression of *S1PR3*, in *n* = 169 from MESA. **C** Expression of genes within a 2 Mb window in THP-1 cells expressing dCas9-KRAB after transduction with an sgRNAs targeting the SV (orange) as compared to a neutral locus control sgRNA (blue). Relative mRNA level of each gene was represented by mean ± standard deviation (SD). *N* = 3 biological replicates, where each replicate is a unique cellular transduction by sgRNA cassette. **P = 0.009. Location of sgRNAs designed for CRISPRi are indicated in Fig. S8C. **D**–**F** *S1PR3* gene editing impaired monocyte differentiation in vitro. **D** Editing efficiency in HSPCs following 3xNLS-SpCas9:sgRNA electroporation with the indicated sgRNA. Gene edits were measured after 4 days of electroporation (*N* = 4 biological replicates). Location of *S1PR3* coding sequence targeting sgRNAs are indicated above. **E** Representative flow cytometry indicating CD13 + CD14 + cell populations from the neutral locus and *S1PR3* targeting group after 12-day differentiation. **F** CD13 + CD14 + percentage in the *S1PR3* targeting group and the neutral locus targeting group. *N* = 4 replicates where each replicate is a unique Cas9:sgRNA electroporation experiment. Mean ± SD, with Student’s two-sided *t*-test.***P < 0.001, ****P < 0.0001. **G**–**J** Human CD34 + HSPCs from three healthy donors were edited by Cas9 RNP electroporation (EP) targeting a neutral locus and *S1PR3* coding sequence infused into NBSGW mice 24 h after electroporation. After 12 weeks, engrafted bone marrow was characterized by immunophenotyping. **G** Indels determined by Sanger sequencing before transplantation. (H––J) Quantification of different human cell types between the neutral locus and *S1PR3* targeting group. Human chimerism, hCD45 + ; Monocytes, hCD45 + CD33 + SSClowCD14 + ; neutrophil, hCD45 + CD33 + SSChighCD16 + . *N* = 3 independent biological replicates, each replicate indicates one mouse. Mean ± SD, 2-sided Mann-Whitney test. *P = 0.025, **P = 0.004.

Given this regulatory potential, we investigated whether the 9q22.1 SV was associated with expression changes of nearby genes. We performed expression quantitative trait loci (eQTL) analysis on the 9q22.1 SV in the TOPMed Multi-Ethnic Study of Atherosclerosis (MESA, using *n* = 169, including both African American and Hispanic/Latino individuals). *Cis*-eQTL analysis in CD14 + monocyte samples, revealed the strongest association to be between the 9q22.1 SV and *S1PR3* compared to all other genes in a 2 Mb window (Fig. 2A). Deletion of this region is significantly associated with decreased abundance of *S1PR3* (P = 5.20E-06) (Fig. 2B). Similar results were observed in peripheral blood mononuclear cells (PBMC), but not in T cells, consistent with a cell type-specific cis-regulatory effect on *S1PR3* expression (Figs. S8A, B).

To experimentally test the regulatory potential of the deleted sequences, we performed CRISPRi with dCas9-KRAB in monocytic THP-1 cells. Three sgRNAs were designed targeting different sequences within the 9q22.1 SV deleted segment (Fig. S8C). CRISPRi with each of these three sgRNAs significantly reduced the expression of *S1PR3* but not other nearby genes (Fig. 2C and Fig. S8D–F). Taken together, the results provide strong evidence that the trait-associated SV deletes a monocyte-specific enhancer that controls the expression of *S1PR3* in monocytes.

To test the functional role of *S1PR3* in monocyte maturation and homeostasis, we edited human CD34 + hematopoietic stem and progenitor cells (HSPCs) with three sgRNAs targeting *S1PR3* or a sgRNA targeting a neutral locus and performed in vitro monocyte differentiation. Each of the *S1PR3*-targeting sgRNAs yielded highly efficient gene edits (95.7% ± 1.9% indels) (Fig. 2D). Compared with the neutral locus targeting control, each of the three *S1PR3-*edited cell populations showed a significant decrease in CD14 + monocyte differentiation efficiency in vitro (*P* < 0.001) (Fig. 2E, F), suggesting monocyte differentiation depends on *S1PR3* expression.

Lastly, to further validate the role of *S1PR3* in human hematopoiesis, we edited human hematopoietic stem and progenitor cells (HSPCs) with sgRNAs targeting a neutral locus or *S1PR3*, and infused the edited HSPCs into immunodeficient NBSGW mice. Human engraftment and multiple-lineage hematopoiesis were analyzed in the mouse bone marrow after 12 weeks. Gene edits were 95.4% in the input HSPC cell product for *S1PR3* and remained consistent (93.7%) in engrafting human cells (Fig. 2G). Overall human hematopoietic chimerism, and the fraction of lymphoid and erythroid lineage cells in the bone marrow was similar between the neutral locus and *S1PR3*-targeting group (Fig. 2H and Fig. S9). We observed a decrease of CD16 + neutrophil percentage (*P* = 0.004) and increase of CD14 + monocyte percentage (P = 0.02) in the bone marrow of *S1PR3*-edited groups (Fig. 2I, J). These results suggest that *S1PR3* loss of function leads to altered human myeloid homeostasis in vivo, consistent with a functional role of *S1PR3* in determining monocyte count.

## Discussion

GWAS have identified thousands of SNVs and small indels that contribute to quantitative hematologic traits but the contribution of SVs to blood cell trait variation has mainly been limited to individuals with rare genetic blood disorders^32–34^. Here we investigated the contribution of SVs to hematologic variation in ancestrally diverse TOPMed participants. Using single variant tests, we show 21 independent SVs were significantly associated with quantitative hematologic traits. These trait-associated SVs ranged in size (~60 bp to >160 kb) and allele frequency. Remarkably, most of these association signals were replicated in independent datasets, suggesting that despite the known challenges associated with SV discovery/genotyping in short-read data^6^, WGS-based SV call-sets can be successfully used to study complex trait variation.

Most trait-associated SVs are located in genomic regions previously associated with blood cell traits and most are not conditionally-independent of SNV/indels at the same loci previously identified through GWAS. One exception was a novel association between the 17p11.2 locus (*KCNJ18* SV) and white blood cell-related phenotypes. *KCNJ18* has no known role in leukocyte biology. It encodes a potassium channel and variants in this gene are associated with the Mendelian disorder, thyrotoxic hypokalemic periodic paralysis [MIM:613239]. Moreover, the trait-associated *KCNJ18* SV is located in a complex region of the genome which includes a segmental duplication near the centromere of chromosome 17. Likely due to this complexity, we did not identify a *KCNJ18* SV representative in replication datasets. Based on these results as well as the associated phenotypic pattern (opposing effects on neutrophil and lymphocyte proportions) additional analyses are required to ensure this finding reflects inherited genetic variation.

Functional annotation indicates most trait-associated SVs are located in non-coding regions of the genome. The majority of trait-associated SVs were predicted to impact regulatory elements or chromatin loop structure and chromatin domain boundaries. Together with LD and conditional analyses, this is consistent with the notion that SVs may provide mechanistic insights for a subset of known GWAS loci. For instance, a chr7 deletion which includes the EPO promoter was recently shown to alter iPSC expression levels of 5 nearby genes^35^, including the genes, *TFR2* and *EPHB4* which are involved in iron metabolism and erythropoiesis^36,37^. In our analyses, this chr7 deletion was present in TOPMed (allele frequency = 0.189) but just missed our significance threshold for association with red cell phenotypes. Notably, in our analyses this chr7 deletion was in near perfect LD with a previously-reported SNV chr7:100729121 (rs4729607)^9^.

In this study, we also identified a monocyte trait-associated SV (602 bp, 9q22.1 deletion) that directly overlaps a monocyte cell type-specific enhancer with accessible chromatin and enhancer signature histone modifications. The enhancer forms a physical interaction with the *S1PR3* gene in monocytes, and the 9q22.1 SV is an eQTL for *S1PR3* expression in monocytes. By using CRISPRi targeting the enhancer, we showed the enhancer positively regulates *S1PR3* in monocyte lineage cells. Prior GWAS have identified SNVs at this locus associated with blood cell traits including monocyte count, but without identification of the causal variant. This trait association represents an experimentally validated example where an ancestry-specific SV appears to underlie an SNV-tagged trait association through effects on cell-type specific gene regulation.

Gene editing of *S1PR3* significantly impacted both in vitro monocyte maturation and monocyte homeostasis in xenograft experiments. *S1PR3*, a receptor for the bioactive lipid sphingosine-1-phosphate (S1P), is a central regulator which drives myeloid differentiation^38^. Complementary to our results, previous studies have shown that *S1PR3* overexpression alone is sufficient to induce myeloid differentiation in human HSC^38^. In addition, *S1PR3* has been implicated in the mobilization from the bone marrow to the peripheral blood of hematopoietic and mesenchymal progenitors^39,40^. The decreased efficiency of in vitro monocyte maturation, and relative increase in the fraction of bone marrow CD14^+^ monocytes with reciprocal decrease of bone marrow CD16^+^ neutrophils of engrafted mice, may suggest that *S1PR3* both plays cell autonomous roles in monocytes during maturation as well as impacts the trafficking of myeloid cells from bone marrow stores to circulating cells in the peripheral blood. Supporting this hypothesis, S1P receptors, which are chemotactically sensitive to S1P gradients, regulate multiple processes, including migration, matrix adhesion, and cell-cell contact. Therefore, the steep gradient of S1P concentration existing between bone marrow and blood might promote monocytes to navigate from the bone marrow to circulation^40^. The biological contributions of *S1PR3* to monocyte maturation and trafficking may be complex and could merit future dedicated study.

In summary, our results from a large ancestrally-diverse population-based data set add further evidence that complex trait association signals may be explained by the presence of structural variation. These findings complement recent WGS-based studies performed in European population isolates demonstrating the contribution of structural variation to complex trait variation (quantitative cardiometabolic and anthropometric traits)^5,17^. Several limitations of our study should be noted: 1) our analyses were restricted to deletions, inversions, and duplication and 2) were restricted to autosomal structural variation. Both of these limitations can be overcome with additional SV association studies that more broadly survey structural variation. In particular, the incorporation of long-read data into SV-based association analyses will greatly improve our understanding of how SVs contribute to hematological and complex trait variation.

## Methods

### TOPMed study population

We included 50,675 participants from 12 TOPMed studies: Genetics of Cardiometabolic Health in the Amish (Amish, *n* = 1090)^41^, Atherosclerosis Risk in Communities Study (ARIC, *n* = 3717)^42^, Mount Sinai BioMe Biobank (BioMe, *n* = 9102)^43^, Coronary Artery Risk Development in Young Adults (CARDIA, *n* = 2966)^44^, Cardiovascular Health Study (CHS, *n* = 3478)^45^ Genetic Epidemiology of COPD Study (COPDGene, *n* = 5595)^46^, Framingham Heart Study (FHS, *n* = 2760)^47^, Genetic Studies of Atherosclerosis Risk (GeneSTAR, *n* = 1494)^48^, Hispanic Community Health Study - Study of Latinos (HCHS_SOL, *n* = 3824)^49^, Jackson Heart Study (JHS, *n* = 3329)^50,51^, Multi-Ethnic Study of Atherosclerosis (MESA, *n* = 2516)^52^, and Women’s Health Initiative (WHI, *n* = 10,804)^53^. The 50,675 TOPMed participants were categorized into discrete ancestry subgroups using a machine learning algorithm, which uses genetically inferred ancestry to refine self-identified ancestry and impute missing values^54^ (see Supplemental Methods). The ancestry composition in this study was 59% European, 24% African, 16% Hispanic/Latino, and 1% Asian (Supplementary Data 1). Only samples with a missingness rate <10% in the structural variant dataset were included in analysis. Further descriptions of the design of the participating TOPMed cohorts and the sampling of individuals within each cohort for TOPMed WGS are provided in the section “Participating TOPMed studies” under Supplemental Methods. All studies were approved by the appropriate institutional review boards (IRBs) and informed consent was obtained from all participants.

### Blood cell trait measurements

Red blood cell, white blood cell and platelet quantitative traits were measured from freshly collected whole blood samples using automated hematology analyzers according to clinical laboratory standards. In studies where multiple blood cell measurements per participant were available, we selected a single measurement for each trait and each participant. Each trait was defined as follows: Hematocrit (HCT) is the percentage of volume of blood that is composed of red blood cells. Hemoglobin (HGB) is the mass per volume (grams per deciliter) of hemoglobin in the blood. Mean corpuscular hemoglobin (MCH) is the average mass in picograms of hemoglobin per red blood cell. Mean corpuscular hemoglobin concentration (MCHC) is the average mass concentration (grams per deciliter) of hemoglobin per red blood cell. Mean corpuscular volume (MCV) is the average volume of red blood cells, measured in femtoliters (fL). RBC count is the count of red blood cells in the blood, by number concentration in millions per microliter. Red cell distribution width (RDW) is the measurement of the ratio of variation in width to the mean width of the red blood cell volume distribution curve taken at + /− one CV. Total white blood cell count (WBC), neutrophil, monocyte, lymphocyte, eosinophil, basophil and platelet count are defined with respect to cell concentration in blood, measured in thousands/microliter. The proportion of neutrophils, monocytes, lymphocytes, or eosinophils were calculated by dividing the respective WBC sub-type count by the total measured WBC. Mean platelet volume (MPV) was measured in fL. For each trait, we identified extreme values that may represent measurement or recording errors or hematologic malignancies and removed them from the analysis.

In a subset of samples from the JHS and HCHS/SOL studies, we evaluated four iron-related phenotypes: serum iron, total iron binding capacity (TIBC), transferrin saturation, and ferritin. Serum iron (μg/dl) was measured by colorimetric assay using a ferrozine reagent (Roche Diagnostics, Indianapolis, IN). Unsaturated iron binding capacity (UIBC) was assayed by colorimetric assay on the same sample, TIBC (μg/dl) was calculated by the formula: TIBC = serum iron + UIBC. Serum ferritin (ng/ml) was measured with Roche reagents using a particle enhanced immunoturbidimetric assay. Transferrin saturation (%) was calculated by the formula: SAT = serum iron/TIBC x 100.

### WGS data and quality control in TOPMed

WGS was performed through the NHLBI TOPMed program on genomic DNA isolated from peripheral blood. WGS was generated to an average depth of 38X by six sequencing centers (Broad Genomics, Northwest Genomics Center, Illumina, New York Genome Center, Baylor, and McDonnell Genome Institute)^55^. Most WGS was performed using PCR-free library preparation, Illumina HiSeq X Ten or NovaSeq instruments and 150 bp paired end reads. Sequencing reads were aligned to the human reference genome (GRCh38) by the TOPMed Informatics Research Center (IRC) using the read mapping pipeline described in Regier, A. et al.^56^.

### Single nucleotide variant and small indel discovery and genotyping in TOPMed

We utilized the TOPMed freeze 8 genotype call set produced by the IRC as previously described^56^. Briefly, SNVs and indels were discovered on a per sample basis, then merged and genotyped across samples. SNV and indel quality control (QC), was performed by calculating Mendelian consistency scores and by applying a support vector machine (SVM) classifier trained on known variant sites and Mendelian inconsistencies. SNV- and indel-based, sample QC measures included: concordance between annotated and inferred genetic sex, concordance between prior array genotype data and TOPMed WGS data, and pedigree checks. Further details regarding data processing, and quality control are described on the TOPMed website (https://www.nhlbiwgs.org/topmed-whole-genome-sequencing-methods-freeze-8) and in a common document accompanying each TOPMed study’s dbGaP accession.

### Structural variant discovery and genotyping in TOPMed

We utilized the TOPMed SV freeze 1.0 call set, which contains 138,134 TOPMed samples. Briefly, SV calls were assessed from each sample separately using Parliament2 pipeline^15^. The Parliament2 pipeline provides the union of SV calls from six different programs: BreakDancer, BreakSeq, CNVnator, Delly, Lumpy and Manta. SV calls were merged across samples using survivor^57^ and filtered using SVTyper^58^. Sample genotypes for each variant were assessed using muCNV^16^. After final filtering, the TOPMed SV freeze 1.0 call set consists of a total of 466,455 autosomal SV sites: 231,817 deletions, 197,412 duplications and 37,226 inversions. Of these, 96,049 had MAC > = 5 in at least one trait and were included in association analyses. For association analysis, the genotypes of each SV were represented in a bi-allelic genotype format (GT = 0/0, 0/1, 1/1), similar to SNVs and small indels generated from the same WGS data.

### Single variant association analysis of SVs and blood cell traits using linear mixed models

Single variant SV association tests for all variants with a minor allele count (MAC) ≥ 5 were performed for each blood cell trait using a two-stage linear mixed model (LMM) approach implemented in the GENESIS software^59,60^. In the first stage, a null model assuming no association between the outcome and any SV was fit, adjusting for the fixed effect covariates of: age at trait measurement; sex; a variable indicating TOPMed study and study phase (study_phase); indicators for stroke, COPD, and VTE; the first 11 PC-AiR^61^ principal components (PCs) of genetic ancestry as estimated from the WGS SNV/indel genotypes. We additionally included as fixed effect covariates, the first 10 principal components estimated from read depth (“batch PCs”). To calculate batch PCs, we first computed the average sequencing depth for every 1 kb genomic region (“bin”) across the 22 autosomes^62^. We removed bins containing repetitive sequences with poor mappability (<1.0 using 50 bp k-mers in GEMTools v1.759) or sequences overlapping known CNVs in the Database of Genomic Variants. Following normalization of the approximately 150,000 remaining bins, we performed Randomized Singular Value Decomposition (rSVD)^63^, to generate batch PCs, which were used to correct for batch and technical artifacts arising from the sequencing process.

In the first-stage null model, a 4th degree sparse empirical kinship matrix (KM) computed with PC-Relate^64^ was included to account for genetic relatedness among participants. To control genomic inflation, we additionally allowed for heterogeneous residual variances by study and ancestry group. Details on how ancestry groups were estimated for this adjustment are in the supplemental methods. Following fitting of the first-stage null model, we performed a rank-based inverse-normal transformation of the marginal residuals, and subsequently rescaled the residuals by their variance prior to transformation. This rescaling allows for clearer interpretation of estimated SV genotypic effect sizes from the subsequent association tests. In the second stage, we fit another LMM using the rank-normalized and rescaled residuals as the outcome, with the same fixed effect covariates, sparse KM, and heterogeneous residual variance model as in Stage 1. The output of the Stage 2 null model was then used to perform genome-wide score tests of genetic association for all SVs that passed the TOPMed SV quality filters and with a minor allele count (MAC) ≥ 5. Missing SV genotype calls were imputed to the mean before performing the association tests. Investigation of QQ plots stratified by MAC showed similar test behavior at all MAC bins (Fig. S2). The total number of unique SVs tested across all traits was 96,049.

Basophils was tested as a binary outcome (basophil count > 0), so the null model was fit as a logistic mixed model using the GMMAT method as implemented in GENESIS, rather than a two-stage LMM. The same fixed effect covariates and sparse KM were used in the null model, and score tests were used for association. Genome-wide significance was defined as 5.0 × 10^−8^ for common variants (MAF > 1%) and 8.0 × 10^−9^ for rare variants (MAF < = 1%)^65^.

### Visualization of SVs associated with blood cell traits

For SVs significantly associated with blood cell traits, we performed additional quality control by visualizing aligned WGS reads in variant samples. Visualization was performed using samplot on the NHLBI Biodata Catalyst cloud computing platform (10.5281/zenodo.3822858). For each SV event, we visualized aligned reads for multiple samples and excluded any SV events that were not clearly supported by the aligned WGS data. Additionally, we selected SV events in instances where Parliament2 identified multiple overlapping SVs by different SV calling algorithms. This was concluded following data visualization and we selected SVs based on the resolution of predicted Parliament2 breakpoints.

### Replication of trait-associated SVs using deCODE genetics and UK Biobank datasets

We performed replication analyses for each TOPMed SV-blood cell trait association signal using deCODE genetics and UKBB datasets. Briefly, SVs were called in Icelanders (deCODE genetics) using 49,962 short-read^18,19^ and 3622 long-read sequenced^17^ individuals. These data were phased and all genotyped variants were imputed into 166,281 individuals using a previously described methodology^66,67^. SVs were called from 150,119 short-read sequenced individuals in UKBB. Three cohorts were used in UKBB with 132,169, 2963, and 3047 sequenced and 431,805, 9633, and 9252 imputed individuals, in British/Irish (XBI), African (XAF) and South Asian (XSA) populations, respectively^20^. For replication analyses, we defined an SV in replication datasets to represent a TOPMed SV, if located within 5 kb of the TOPMed SV and if the two SV sizes overlapped by at least 25%. We considered SVs as representative if they were the same structural variant type (e.g. both SVs were deletions) and had similar minor allele frequencies in the relevant population. We tested for association for all representative SVs and their corresponding phenotypes based on the linear mixed model implemented in BOLT-LMM^68^ and described in^17,20^. We considered an association to be replicated if at least one of the *p*-values from the deCODE, UKBB British, UKBB African, or UKBB South Asian cohorts was < 0.05/(number of its representative SVs in given dataset).

### Functional annotation of SVs

We annotated genome sequence information for SVs significantly associated with blood cell traits using AnnoSV^69^. From AnnoSV, we ascertained gene annotations (based on RefSeq, ENSEMBL), the presence of similar SVs in genomic databases (e.g., DGV) and breakpoint information including overlap with repetitive elements. To understand the potential impact of trait-associated SVs on non-coding/regulatory regions, we cross-referenced SVs with five different genomic annotations including, frequently interacting regions (FIREs) from Hi-C data^30,31^, topologically associating domain (TAD) boundaries, cell-type specific regulatory networks from super interactive promoters (SIPs)^29^, candidate *cis* regulatory elements (cCREs) from ENCODE, and chromatin interaction information from chromatin conformation data including Hi-C^27^ and promoter capture Hi-C (pcHi-C)^28^.

### Linkage disequilibrium and conditional analyses for trait-associated SVs

For each blood cell trait, we performed conditional association analyses to determine which genome-wide significant SVs remained significant following adjustment for (1) previously reported GWAS variants and (2) SNV and small indels previously detected in TOPMed^21–23^. To address the first question, we used variants detected in multi-ancestry and European populations reported in Chen et al. 2020 and Vuckovic et al. 2020^9,10^. The genome-wide significant variants from Chen et al. 2020 and Vuckovic et al. 2020 were matched to TOPMed SNVs and small indels that passed the IRC quality filters based on chromosome, position, and alleles. For each trait, the set of matched variants on each chromosome was then LD-pruned at r^2^ = 0.8 in the sample set of the non-conditional analysis for that trait, preferentially keeping variants with lower p-values in the TOPMed analysis sample set. Switching the LD threshold to r^2^ = 0.999 for pruning produced very similar *p*-values and did not add or remove any significant loci. This pruned set of variants were combined across chromosomes and included as fixed effect covariates in the null model using the same fully-adjusted two-stage LMM association testing procedure described above^59,60^.To identify SVs independent of GWAS variants detected in the TOPMed data, we used a similar procedure, starting with any SNV or small indels with *P* < 5.0 × 10^−8^ in single variant association tests using the same sample set for the trait. This set of variants was then LD-pruned at *r*^2^ = 0.8, again preferentially keeping variants with lower p-values, and included as fixed effects in a two-stage LMM association testing^59,60^.

To investigate the proportion of SVs in LD with known, blood-cell trait SNVs/indels, we additionally calculated LD (r^2^) for genotypes of 6652 previously-reported SNVs/indels from European ancestry samples^10^ and SVs with MAC ≥ 5 on the same chromosome. Only TOPMed participants with inferred European ancestry (*N* = 29,244) were used for the LD calculation. Each SNV/indel was then matched to the SV with the highest r^2^ value.

### eQTL analysis

RNA-sequencing (RNA-seq) data for eQTL analysis were derived from a subsample of African American and Hispanic/Latino TOPMed MESA cohort participants using blood samples derived from either MESA exam 1 (2000-2002) or MESA exam 5 (2010-2012) as previously described^70^. RNA sequencing was performed on peripheral blood mononuclear cells (PBMC) from 297 African American and 246 Hispanic/Latino MESA participants at exam 1 and from isolated monocytes and T lymphocytes from 77 African American and 92 Hispanic/Latino MESA participants at exam 5 RNA-seq data was processed following the TOPMed harmonized RNA-seq pipeline. Specifically, gene-level expression was quantified by RSEM v1.3.0. We performed cis-eQTL analysis (± 1 Mb) to identify genes whose expression is associated with 9q22.1 SV using Matrix eQTL^71^.

### Hematopoietic cell lines

THP-1 cells (Cat# TIB-202) were obtained from the American Type Culture Collection and cultured in RPMI 1640 (Thermo Fisher Scientific, USA). To make the complete growth medium, the following components were added: 2-mercaptoethanol (Cat# 21985-023, Thermo Fisher Scientific) to a final concentration of 0.05 mM; fetal bovine serum (Cytiva) to a final concentration of 10%.

### Primary hematopoietic cells and monocyte-macrophage differentiation

Human CD34^+^ HSPCs from mobilized peripheral blood of deidentified healthy donors were purchased from Fred Hutchinson Cancer Research Center, Seattle, Washington. CD34^+^ HSPCs were cultured in StemSpan SFEM medium (Cat# 09650, STEMCELL Technologies) supplemented with 1x StemSpan CD34^+^ expansion supplement (Cat# 02691, STEMCELL Technology). To induce monocyte-macrophage differentiation from CD34^+^ HSPCs, the cytokine cocktail of M-CSF 30 ng/mL, FLT3-Ligand 100 ng/mL, SCF 50 ng/ml, IL-3 5 ng/mL, IL-6 3 ng/ul and L-Glutamine 2 mM was supplemented to the culture media for 11 days before analysis. GM-CSF 5 ng/ml was supplemented in the culture media for the first 4 days. All cytokines of human origin and from PeproTech.

### CRISPR/Cas9 guide design, cloning, lentiviral vector production and transduction and 3xNLS-SpCas9 preparation

Streptococcus pyogenes Cas9 (SpCas9) guide RNAs that either target *S1PR3* coding sequence or bind near the structural deletion (9:88923551-8892452) were identified using computational algorithms with prioritization for on-target efficiency and reduced off-target effects (CRISPOR: http://crispor.tefor.net/). For RNP experiments, the chemically modified sgRNAs were synthesized by Integrated DNA Technologies. SpCas9 proteins were expressed and purified as previously described^72^.

For CRISPRi experiments, oligos (from GENEWIZ company) were annealed and ligated into LentiGuide-Puro (Addgene, Cat#52963). Following lentiviral production and transduction into THP-1 cell lines with stable dCas9-KRAB expression (Addgene, Cat#89567), 10 μg/ml blasticidin and 1 μg/ml puromycin were added to select for sgRNA expression in cells with stable dCas9-KRAB expression. The sequence of sgRNAs are summarized in Supplementary Data 4.

### CRISPR-Cas9 genome editing in CD34^+^ HSPCs and THP-1 cells

CD34^+^ HSPCs and THP-1 cells were maintained in their favorable medium (see before) 24 h before electroporation. Approximately 100,000 cells per condition were electroporated using the Lonza 4D nucleofector with 100 pmol 3xNLS-SpCas9 protein and 300 pmol modified sgRNA targeting the locus of interest. In addition to mock treated cells, “safe-targeting” RNPs were used as experimental controls as indicated in each figure legend^73^. After electroporation, cells were induced for monocyte-macrophage differentiation as described previously. Genomic DNA was isolated from an aliquot of cells, the sgRNA targeted locus was amplified by PCR which was subject to Sanger sequencing and then TIDE analysis to quantify indel spectrum at day 4 after electroporation.

### Determination of target gene expression

Total RNA was extracted from cell cultures 4 days after electroporation using the RNeasy Plus Mini Kit (QIAGEN) and reverse transcribed using the iScript cDNA synthesis kit (Biorad) according to the manufacturer’s instructions. Expression of target genes was quantified using real-time RT-qPCR with GAPDH as an internal control. All gene expression data represent the mean of at least three biological replicates. Primers for PCR are summarized in Supplementary Data 5. Since PCR primers could not be designed for RP5-1050E16.1, this gene was excluded from further analysis.

### Flow cytometry analysis

For analysis of surface markers, cells were stained in PBS containing 2% (w/v) BSA, with the following antibodies (from Biolegend): anti-human CD34 (clone 581, Cat# 343504, 1:200), anti-human CD33 (clone P67.6, Cat# 366608, 1:200), anti-human CD117 (clone 104D2, Cat# 313205, 1:200), anti-human CD16 (clone 3G8, Cat# 302046, 1:100), anti-human CD14 (clone 63D3, Cat# 367117, 1:200). Monocyte and macrophage were indicated by anti-human CD14^+^. Flow cytometry data were acquired on a LSRII or LSR Fortessa (BD Biosciences) and analyzed using FlowJo software (Tree Star).

### Immunophenotyping of human CD34^+^ HSPCs xenograft from NBSGW mice

NOD.Cg-KitW-41J Tyr þ Prkdcscid Il2rgtm1Wjl (NBSGW) mice were obtained from Jackson Laboratory (Stock 026622) and kept in 12 h of light at room temperature. CD34^+^ HSPCs were maintained and edited as described above. Cells were allowed to recover for 24 h after electroporation, and then infused by retro-orbital injection into non-irradiated NBSGW female mice. Bone marrow was isolated for human xenograft analysis 16 weeks after human CD34^+^ HSPCs engraftment. For flow cytometry analysis, bone marrow cells were first incubated with Human TruStain FcX (BioLegend, Cat# 422302) and TruStainfcX (anti-mouse CD16/32, 101320, BioLegend) blocking antibodies for 10 min and then stained with marker panels designed for multi-lineage analysis. The following antibodies were used: From BD: anti-mouse CD45 (clone 30-F11, Cat# 561487, 1:100), anti-human CD45 (clone HI30, Cat# 560367, 1:200) From BioLegend: anti-human CD34 (clone 581, Cat# 343504, 1:200), anti-human CD33 (clone P67.6, Cat# 366608, 1:200), anti-human CD117 (clone 104D2, Cat# 313205, 1:200), anti-human CD16 (clone 3G8, Cat# 302046, 1:100), anti-human CD14 (clone 63D3, Cat# 367117, 1:200), anti-human CD235a (clone HI264, Cat# 349104, 1:100), anti-human CD3 (clone UCHT1, Cat# 300412, 1:200), anti-human CD19 (clone HIB19, Cat# 302212, 1:200), and Fixable Viability Dye eFluor 780 for live/dead staining (65-0865-14, Thermo Fisher, 1:10000). Percentage human engraftment was calculated as hCD45^+^ cells/(hCD45^+^ + mCD45^+^ cells). The mouse work was performed, following Boston Children’s Hospital institutional review board (IRB) approval.

### DNase I-sequencing, chromatin, and Hi-C datasets

DNase I-sequencing and histone modification datasets, including H3K27ac, H3K4me1 and H3K4me3, were downloaded from the ENCODE project^26^, and were then analyzed using WashU Epigenome Browser online software^74^. In situ Hi-C maps of DNA interactions in human monocytes and macrophages were previously described^27^, and visualized by HUGIn2 (Hi-C Unifying Genomic Interrogator, http://hugin2.genetics.unc.edu/Project/hugin/)^75^.

### Reporting summary

Further information on research design is available in the Nature Portfolio Reporting Summary linked to this article.

## Supplementary information

Supplementary Information

Description of Additional Supplementary Files

Supplementary Data 1

Supplementary Data 2

Supplementary Data 3

Supplementary Data 4

Supplementary Data 5

Supplementary Data 6

Reporting Summary

## Data Availability

Data for each participating study can be accessed through dbGaP with the corresponding accession numbers (Amish, phs000956; ARIC, phs001211; BioMe, phs001644; CARDIA, phs001612; CHS, phs001368; COPDGene, phs000951; FHS, phs000974; GeneSTAR, phs001218; HCHS/SOL, phs001395; JHS, phs000964; MESA, phs001416; WHI, phs001237).

## Code availability

Code to implement the associations analyses is available on GitHub at https://github.com/UW-GAC/analysis_pipeline.
