# Supplementary material for: Whole genome sequencing identifies structural variants contributing to hematologic traits in the NHLBI TOPMed program
Source: Nat Commun. 2022 Dec 8;13:7592. doi: 10.1038/s41467-022-35354-7 (PMC9732337; doi:10.1038/s41467-022-35354-7)
Supplement: Supplementary file 1 — Supplementary Information [file 41467_2022_35354_MOESM1_ESM.pdf]

SUPPLEMENTARY INFORMATION

Participating studies

Amish

The Amish Complex Disease Research Program includes a set of large community-based studies focused largely on cardiometabolic health carried out in the Old Order Amish (OOA) community of Lancaster, Pennsylvania (http://medschool.umaryland.edu/endocrinology/amish/research-program.asp). The OOA population of Lancaster County, PA immigrated to the Colonies from Western Europe in the early 1700's. There are now over 30,000 OOA individuals in the Lancaster area, nearly all of whom can trace their ancestry back 12-14 generations to approximately 700 founders. Investigators at the University of Maryland School of Medicine have been studying the genetic determinants of cardiometabolic health in this population since 1993. To date, over 7,000 Amish adults have participated in one or more of our studies.

Due to their ancestral history, the OOA are enriched for rare exonic variants that arose in the population from a single founder (or small number of founders) and propagated through genetic drift. Many of these variants have large effect sizes and identifying them can lead to new biological insights about health and disease. The parent study for this WGS project provides one (of multiple) examples. In our parent study, we identified through a genome-wide association analysis a haplotype that was highly enriched in the OOA that is associated with very high LDL-cholesterol levels. At the present time, the identity of the causative SNP – and even the implicated gene – is not known because the associated haplotype contains numerous genes, none of which are obvious lipid candidate genes. A major goal of the WGS that will be obtained through the NHLBI TOPMed Consortium will be to identify functional variants that underlie some of the large effect associations observed in this unique population. All study protocols were approved by the institutional review board at the University of Maryland Baltimore. Informed consent was obtained from each study participant.

ARIC

The ARIC study is a population-based prospective cohort study of cardiovascular disease sponsored by the National Heart, Lung, and Blood Institute (NHLBI). ARIC included 15,792 individuals, predominantly European American and African American, aged 45-64 years at baseline (1987-89), chosen by probability sampling from four US communities. Cohort members completed three additional triennial follow-up examinations, a fifth exam in 2011-2013, a sixth exam in 2016-2017, and a seventh exam in 2018-2019. The ARIC study has been described in detail previously (The ARIC Investigators. The Atherosclerosis Risk in Communities (ARIC) study: Design and objectives. American Journal of Epidemiology 1989;129:687-702). All ARIC participants provided written informed consent, and the study was approved by the Institutional Review Boards of the participating centers.

BioMe

The Charles Bronfman Institute for Personalized Medicine at Mount Sinai Medical Center (MSMC), BioMe Biobank, founded in September 2007, is an ongoing, broadly-consented electronic health record-linked clinical care biobank that enrolls participants non-selectively from the Mount Sinai Medical Center patient population. The MSMC serves diverse local communities of upper Manhattan, including Central Harlem (86% African American), East Harlem (88% Hispanic/Latino), and Upper East Side (88% Caucasian/White) with broad health disparities. The BioMe cohort was approved by the Institutional Review Board at the Icahn School of Medicine at Mount Sinai. All BioMe participants provided written, informed consent for genomic data sharing.

CARDIA

The Coronary Artery Risk Development in Young Adults (CARDIA) Study is a study examining the development and determinants of clinical and subclinical cardiovascular disease and their risk factors. It began in 1985-1986

with a group of 5,115 black and white men and women aged 18-30 years. The participants were selected so that there would be approximately the same number of people in subgroups of race, gender, education (high school or less and more than high school) and age (18-24 and 25-30) in each of 4 centers: Birmingham, AL; Chicago, IL; Minneapolis, MN; and Oakland, CA. All CARDIA participants provided informed consent, and the study was approved by the Institutional Review Boards of the University of Alabama at Birmingham and the University of Texas Health Science Center at Houston.

CHS

The Cardiovascular Health Study (CHS) is a population-based cohort study of risk factors for coronary heart disease and stroke in adults 65 years and older conducted across four field centers 2. The original predominantly European ancestry cohort of 5,201 persons was recruited in 1989-1990 from random samples of people on Medicare eligibility lists from four US communities. Subsequently, an additional predominantly African-American cohort of 687 persons was enrolled for a total sample of 5,888. Institutional review committees at each field center approved the CHS, and participants gave informed consent. Blood samples were drawn from all participants at their baseline examination, and DNA was subsequently extracted from available samples. These analyses were limited to participants with available DNA who also consented to genetic studies. Participants were examined annually from enrollment to 1999 and continued to be under surveillance for stroke following 1999. All CHS participants provided informed consent, and the study was approved by the Institutional Review Board [or ethics review committee] of University Washington.

COPDGene

COPDGene (also known as the Genetic Epidemiology of COPD Study) is an NIH-funded, multicenter study. A study population of more than 10,000 smokers (1/3 African American and 2/3 non-Hispanic White) has been characterized with a study protocol including pulmonary function tests, chest CT scans, six minute walk testing, and multiple questionnaires. Five years after this initial visit, all available study participants are being brought back for a follow-up visit with a similar study protocol. This study has been used for epidemiologic and genetic studies. Previous genetic analysis in this study has been based on genome-wide SNP genotyping data. Approximately 1,900 subjects underwent whole genome sequencing in this NHLBI WGS project, including severe COPD subjects and resistant smoking controls. The COPDGene Study web site is: http://www.copdgene.org/. All COPDGene participants provided written informed consent, and the study was approved by the Institutional Review Boards of the participating clinical centers.

FHS

FHS is a three-generation, single-site, community-based, ongoing cohort study that was initiated in 1948 to investigate prospectively the risk factors for CVD including stroke. It now comprises 3 generations of participants: the Original cohort followed since 1948; their Offspring and spouses of the Offspring, followed since 1971 4; and children from the largest Offspring families enrolled in 2002 (Gen 3). The Original cohort enrolled 5,209 men and women who comprised two-thirds of the adult population then residing in Framingham, MA. Survivors continue to receive biennial examinations. The Offspring cohort comprises 5,124 persons (including 3,514 biological offspring) who have been examined approximately once every 4 years. The Gen 3 cohort contains 4,095 participants. The Framingham Heart Study was approved by the Institutional Review Board of the Boston University Medical Center. All study participants provided written informed consent.

GeneSTAR

In 1982 The Johns Hopkins Sibling and Family Heart Study was created to study patterns of coronary heart disease and related risk factors in families with early-onset coronary disease, identified from10 Baltimore area hospitals. Renamed in 2003, the Genetic Study of Atherosclerosis Risk (GeneSTAR) continues to study mechanisms of coronary heart disease and stroke in families using novel models and exciting new methods. GeneSTAR is a family-based study including initially healthy brothers and sisters identified from probands with

early-onset coronary disease, along with the healthy offspring of the siblings and the probands. The goal is to discover and amplify mechanisms of stroke and coronary heart disease. Our African American and European American family cohort has undergone extensive screening, genetic testing, and follow-up for new cardiovascular disease, stroke, and other clinical events for 5 to 38 years. All participants provided written informed consent and the study was approved by the Johns Hopkins Medicine Institutional Review Board.

HCHS/SOL

The Hispanic Community Health Study/Study of Latinos (HCHS/SOL) is a multi-center study of Hispanic/Latino populations with the goal of determining the role of acculturation in the prevalence and development of diseases, and to identify other traits that impact Hispanic/Latino health. The study is sponsored by the National Heart, Lung, and Blood Institute (NHLBI) and other institutes, centers, and offices of the National Institutes of Health (NIH). Recruitment began in 2006 with a target population of 16,000 persons of Cuban, Puerto Rican, Dominican, Mexican or Central/South American origin. Participants were recruited through four sites affiliated with San Diego State University, Northwestern University in Chicago, Albert Einstein College of Medicine in Bronx, New York, and the University of Miami. Recruitment was implemented through a two-stage area household probability design. The study enrolled 16,415 participants who were self-identified Hispanic/Latino and aged 18-74 years, and the extensive psycho-social and clinical assessments were conducted during 2008-2011. Annual telephone follow-up interviews are ongoing since study inception. During the 2014-2017 second visit, the participants were re-examined again for various health outcomes of interest. This study was approved by the institutional review boards (IRBs) at each field center, where all participants gave written informed consent, and by the Non-Biomedical IRB at the University of North Carolina at Chapel Hill, to the HCHS/SOL Data Coordinating Center. All IRBs approving the study are: Non-Biomedical IRB at the University of North Carolina at Chapel Hill. Chapel Hill, NC; Einstein IRB at the Albert Einstein College of Medicine of Yeshiva University. Bronx, NY; IRB at Office for the Protection of Research Subjects (OPRS), University of Illinois at Chicago. Chicago, IL; Human Subject Research Office, University of Miami. Miami, FL; Institutional Review Board of San Diego State University. San Diego, CA.

JHS

The Jackson Heart Study (JHS, https://www.jacksonheartstudy.org/jhsinfo/) is a large, community-based, observational study whose participants were recruited from urban and rural areas of the three counties (Hinds, Madison and Rankin) that make up the Jackson, MS metropolitan statistical area (MSA). Participants were enrolled from each of 4 recruitment pools: random, 17%; volunteer, 30%; currently enrolled in the Atherosclerosis Risk in Communities (ARIC) Study, 31% and secondary family members, 22%. Recruitment was limited to non-institutionalized adult African Americans 35-84 years old, except in a nested family cohort where those 21 to 34 years of age were also eligible. The final cohort of 5,306 participants included 6.59% of all African American Jackson MSA residents aged 35-84 during the baseline exam (N-76,426, US Census 2000). Among these, approximately 3,700 gave consent that allows genetic research and deposition of data into dbGaP, with 3406 participants with post-QC TOPMed whole genome sequencing data. Major components of three clinic examinations (Exam 1 – 2000-2004; Exam 2 – 2005-2008; Exam 3 – 2009-2013) include medical history, physical examination, blood/urine analytes and interview questions on areas such as: physical activity; stress, coping and spirituality; racism and discrimination; socioeconomic position; and access to health care. Extensive clinical phenotyping includes anthropometrics, electrocardiography, carotid ultrasound, ankle-brachial blood pressure index, echocardiography, CT chest and abdomen for coronary and aortic calcification, liver fat, and subcutaneous and visceral fat measurement, and cardiac MRI. At 12-month intervals after the baseline clinic visit (Exam 1), participants have been contacted by telephone to: update information; confirm vital statistics; document interim medical events, hospitalizations, and functional status; and obtain additional sociocultural information. Questions about medical events, symptoms of cardiovascular disease and functional status are repeated annually. Ongoing cohort surveillance includes abstraction of medical records and death certificates for relevant International Classification of Diseases (ICD) codes and adjudication of nonfatal events and deaths.

CMS data are currently being incorporated into the dataset. The JHS study was approved by Jackson State University, Tougaloo College, and the University of Mississippi Medical Center IRBs, and all participants provided written informed consent.

MESA

The MESA study is a study of the characteristics of subclinical cardiovascular disease (disease detected non-invasively before it has produced clinical signs and symptoms) and the risk factors that predict progression to clinically overt cardiovascular disease or progression of the subclinical disease 7. MESA researchers study a diverse, population-based sample of 6,814 asymptomatic men and women aged 45-84. Thirty-eight percent of the recruited participants are white, 28 percent African-American, 22 percent Hispanic, and 12 percent Asian, predominantly of Chinese descent. Participants were recruited from six field centers across the United States: Wake Forest University, Columbia University, Johns Hopkins University, University of Minnesota, Northwestern University and the University of California - Los Angeles. All MESA participants provided written informed consent, and the study was approved by the Institutional Review Boards at The Lundquist Institute (formerly Los Angeles BioMedical Research Institute) at Harbor-UCLA Medical Center, University of Washington, Wake Forest School of Medicine, Northwestern University, University of Minnesota, Columbia University, and Johns Hopkins University.

WHI

The Women's Health Initiative (WHI) is a long-term, prospective, multi-center cohort study that investigates post-menopausal women's health. WHI was funded by the National Institutes of Health and the National Heart, Lung, and Blood Institute to study strategies to prevent heart disease, breast cancer, colon cancer, and osteoporotic fractures in women 50-79 years of age. WHI involves 161,808 women recruited between 1993 and 1998 at 40 centers across the US. The study consists of two parts: the WHI Clinical Trial which was a randomized clinical trial of hormone therapy, dietary modification, and calcium/Vitamin D supplementation, and the WHI Observational Study, which focused on many of the inequities in women's health research and provided practical information about the incidence, risk factors, and interventions related to heart disease, cancer, and osteoporotic fractures. All WHI participants provided informed consent and the study was approved by the Institutional Review Board (IRB) of the Fred Hutchinson Cancer Research Center.

deCODE

All participants who donated samples gave informed consent and the National Bioethics Committee of Iceland approved the study which was in agreement with conditions issued by the Data Protection Authority of Iceland (VSN_140-015). Personal identities of the participant's data and biological samples were encrypted by a third-party system (Identify Protection System), approved and monitored by the Data Protection Authority.

Supplementary Methods

Genetic Ancestry and Relatedness

Principal components (PCs) of genetic ancestry and pairwise relatedness measures were estimated for all 140,062 samples included in the TOPMed 'Freeze 8' data release. Autosomal genetic variants passing the quality filter with a MAF > 0.01 and missing call rate < 0.01 were LD-pruned with an r^2 threshold of 0.1 to obtain a set of 638,486 effectively independent variants for genetic ancestry and relatedness estimation. PC-AiR was used to obtain ancestry informative PCs robust to familial relatedness; the first 11 PCs showed evidence of population structure. PC-Relate was then used to estimate pairwise kinship coefficients (KCs) for all pairs of samples, conditional on the genetic ancestry captured by PC-AiR PCs 1-11; these KC estimates reflect only recent genetic relatedness, e.g. due to pedigree structure. The PC-Relate KC estimates were used to construct a 4th degree sparse, block-diagonal, empirical kinship matrix (KM) for association testing, any pair of samples with estimated

KC > 2^(-11/2) ~ 0.022 were clustered in the same block; all KC estimates within a block of samples were kept, regardless of value; and all KC estimates between blocks were set to 0. By using a sparse block-diagonal KM, the association tests are more computationally efficient yet recent genetic relatedness is still accounted for. We subset the freeze-wide PCs and sparse KM to the appropriate set of participants for each analysis.

Classification of race/ethnicity groups using HARE

Ancestry groups were based on a combination of participants reported race/ethnicity and genetic ancestry represented by PCs from PC-AiR. To infer race/population group membership for participants with missing values, we used the HARE method, a machine learning algorithm that uses a support vector machine (SVM) to determine stratum assignment, taking as input genetically estimated PC values and reported race/ethnicity for each participant. Strata are defined by the unique reported race/ethnicity values provided, then the HARE SVM uses the input (training) data to learn the probability of stratum membership across the entire PC space. The output of HARE consists of multinomial probability vectors of stratum membership for each participant. HARE was run on a subset of samples included in the TOPMed Freeze 8 data release; specifically, samples for participants from non-US-based studies and the Amish participants (because they were very distinct in PC space) were excluded from the HARE analysis. HARE was run using the first 9 PC-AiR PCs generated on this subset of samples to represent genetic ancestry with the following reported race/population groups: Asian, Black, Central American, Cuban, Dominican, Mexican, Puerto Rican, South American, and White. The genetic data from the 31,918 participants with either unreported or non-specific (e.g. 'Multiple' or 'Other') race and population membership was included in the HARE analysis, but they were not used to train the SVM. These participants were assigned to a population stratum based on their highest HARE output probability of membership. All other participants remained in the population stratum corresponding to their reported race/population group. Amish participants were assigned to their own stratum.

Supplementary Figures

Figure S1. QQ plots of the marginal structural variant analyses in TOPMed. All p-values are derived from two-sided t-tests and are not adjusted for multiple comparisons. (A) HCT; (B) HGB; (C) MCH; (D) MCHC; (E) MCV; (F) RBC; (G) RDW; (H) BASO_binary; (I) EOSIN; (J) LYMPHS; (K) MONOS; (L) NEUTRO; (M) WBC; (N) EOSIN%; (O) LYMPHS%; (P) MONOS%; (Q) NEUTRO%; (R) MPV; (S) PLT; (T) FERRITIN; (U) IRON; (V) SAT; (W) UIBC; (X) TIBC.

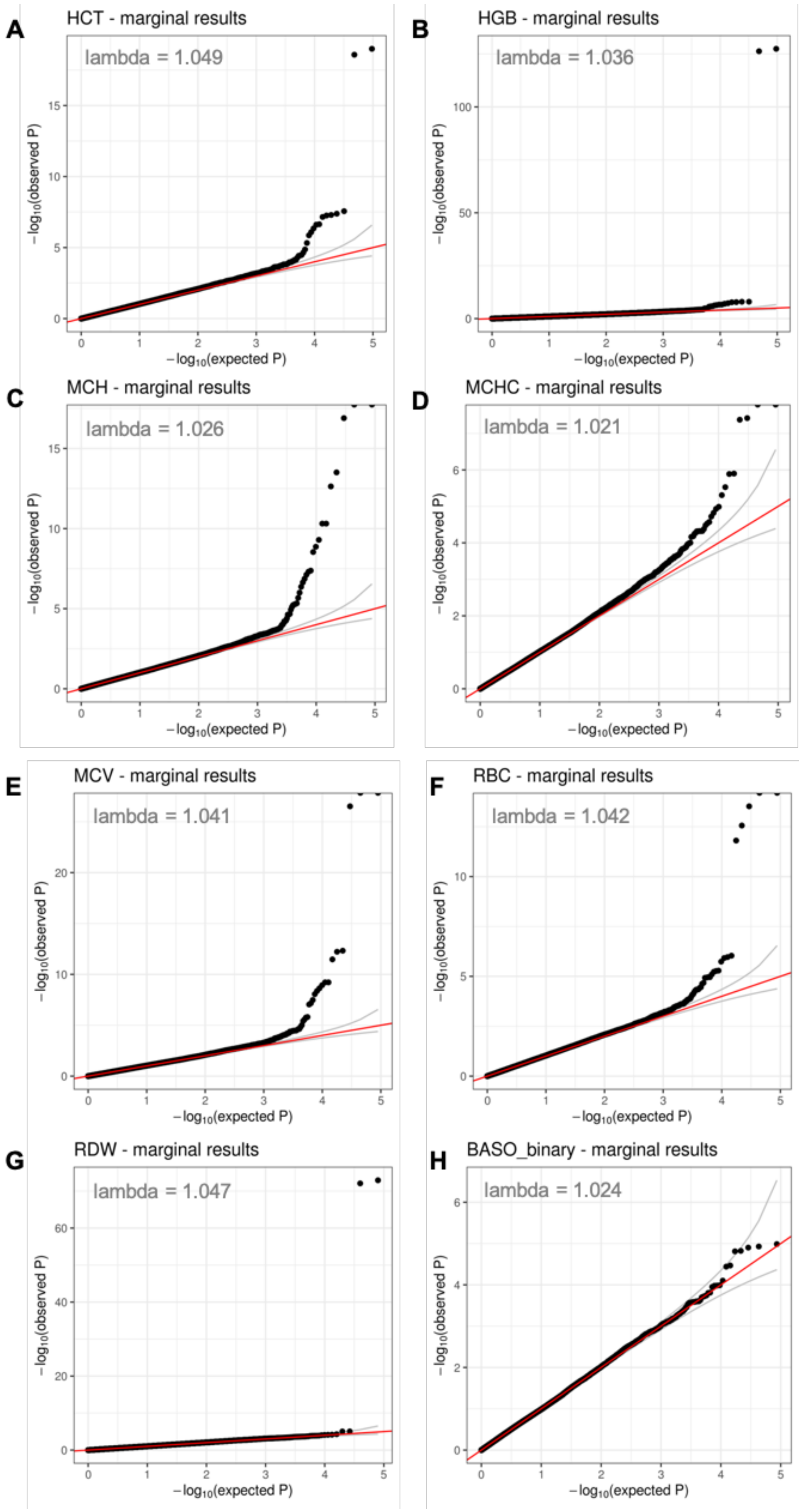
A
HCT - marginal results
lambda = 1.049
B
HGB - marginal results
lambda = 1.036
C
MCH - marginal results
lambda = 1.026
D
MCHC - marginal results
lambda = 1.021
E
MCV - marginal results
lambda = 1.041
F
RBC - marginal results
lambda = 1.042
G
RDW - marginal results
lambda = 1.047
H
BASO_binary - marginal results
lambda = 1.024
−log10(observed P)
−log10(expected P)

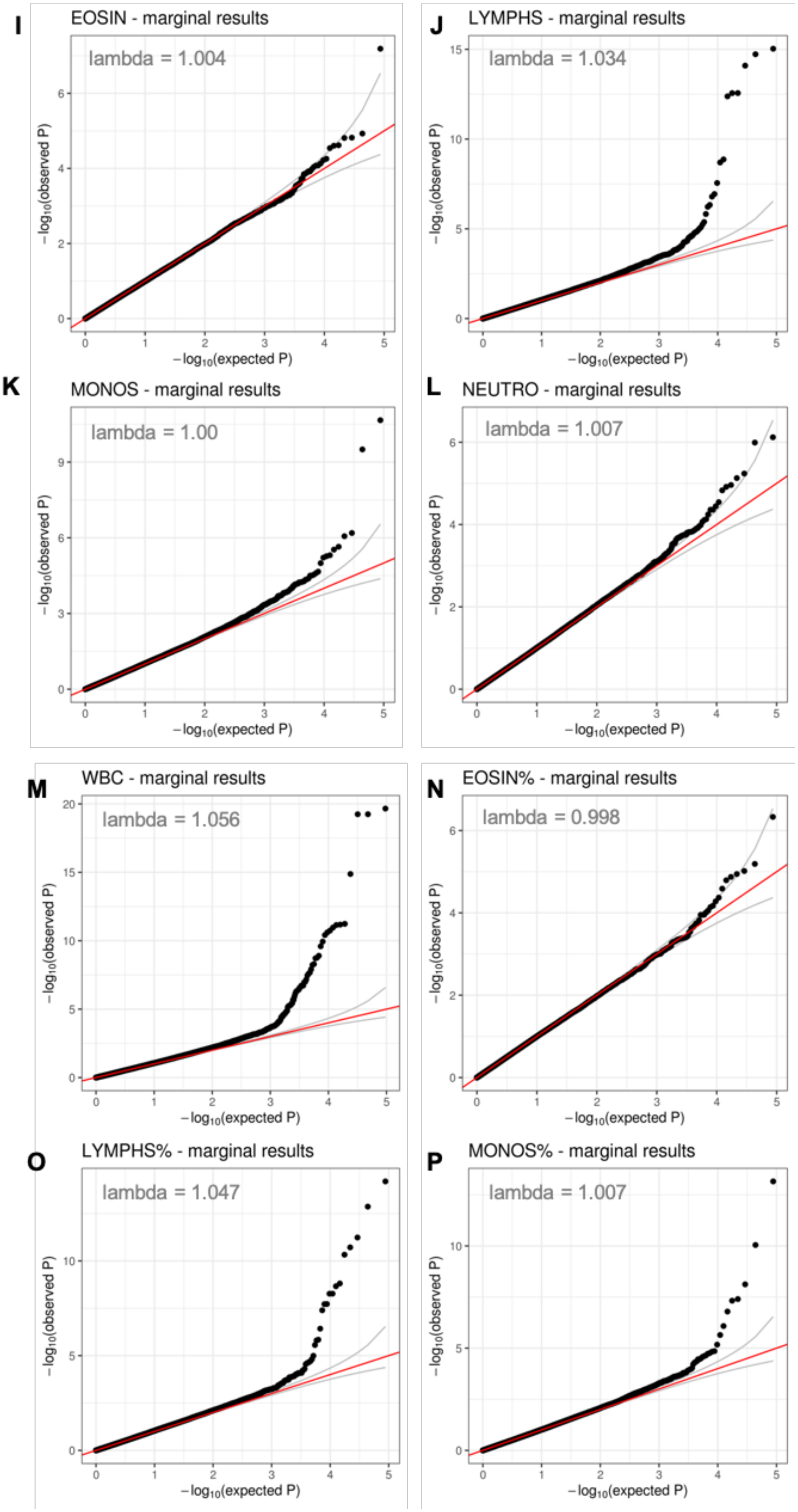

I
EOSIN - marginal results
lambda = 1.004
J
LYMPHS - marginal results
lambda = 1.034
K
MONOS - marginal results
lambda = 1.00
L
NEUTRO - marginal results
lambda = 1.007
M
WBC - marginal results
lambda = 1.056
N
EOSIN% - marginal results
lambda = 0.998
O
LYMPHS% - marginal results
lambda = 1.047
P
MONOS% - marginal results
lambda = 1.007
$-\log_{10}$(observed P)
$-\log_{10}$(expected P)

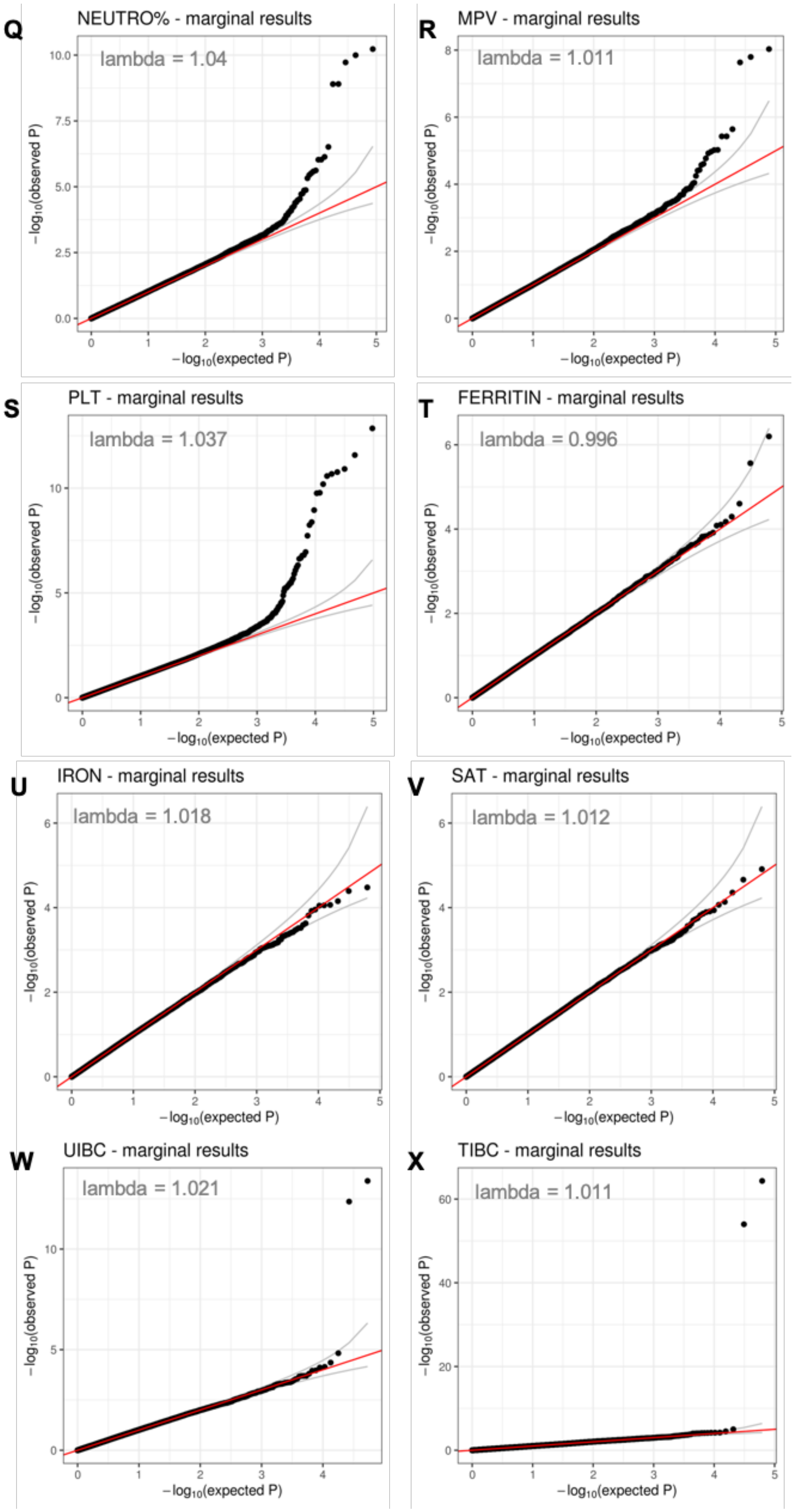
Q
NEUTRO% - marginal results
lambda = 1.04
R
MPV - marginal results
lambda = 1.011
S
PLT - marginal results
lambda = 1.037
T
FERRITIN - marginal results
lambda = 0.996
U
IRON - marginal results
lambda = 1.018
V
SAT - marginal results
lambda = 1.012
W
UIBC - marginal results
lambda = 1.021
X
TIBC - marginal results
lambda = 1.011
−log10(observed P)
−log10(expected P)

Figure S2.QQ plots of the marginal structural variant analyses in TOPMed stratified by minor allele count. The ranges for minor allele count are labeled on the top of each QQ plot. All p-values are derived from two-sided t-tests and are not adjusted for multiple comparisons. (A) HCT; (B) HGB; (C) MCH; (D) MCHC; (E) MCV; (F) RBC; (G) RDW; (H) BASO_binary; (I) EOSIN; (J) LYMPHS; (K) MONOS; (L) NEUTRO; (M) WBC; (N) EOSIN%; (O) LYMPHS%; (P) MONOS%; (Q) NEUTRO%; (R) MPV; (S) PLT; (T) FERRITIN; (U) IRON; (V) SAT; (W) UIBC; (X) TIBC.

A

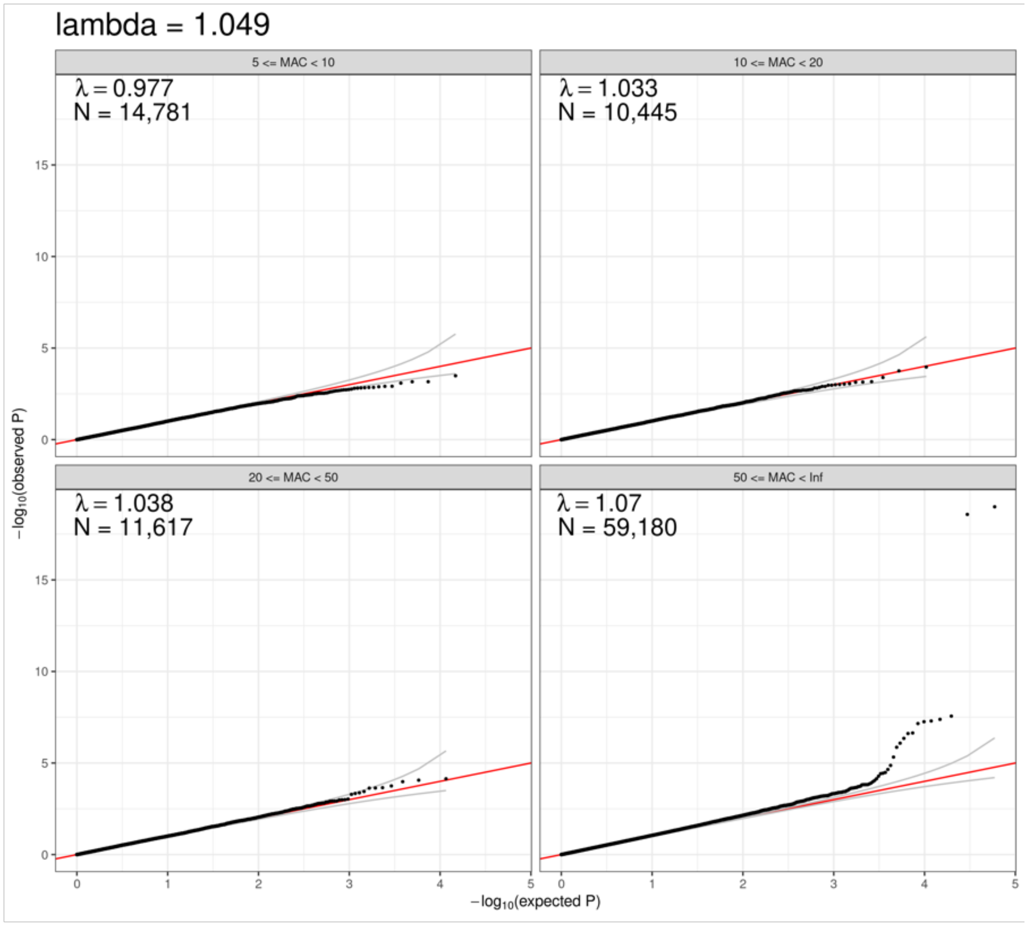
lambda = 1.049
5 <= MAC < 10
λ = 0.977
N = 14,781
10 <= MAC < 20
λ = 1.033
N = 10,445
20 <= MAC < 50
λ = 1.038
N = 11,617
50 <= MAC < Inf
λ = 1.07
N = 59,180
−log10(observed P)
−log10(expected P)

B

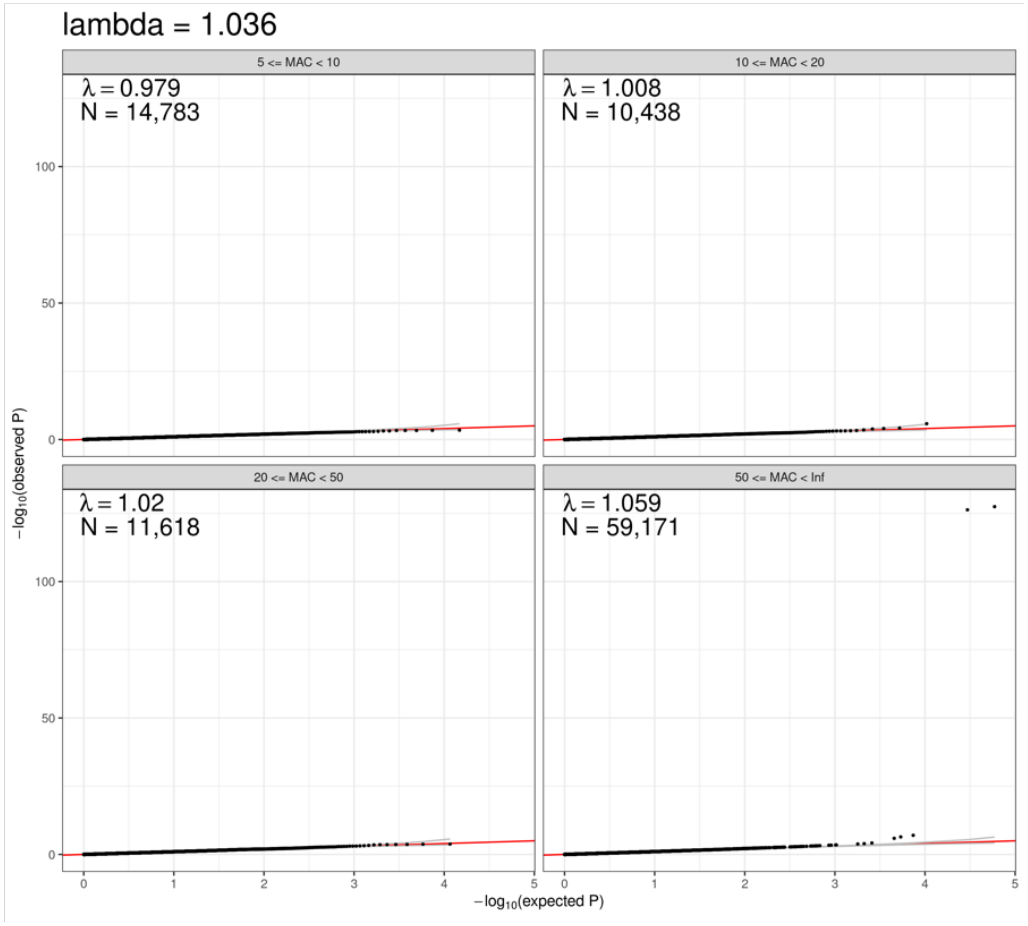
lambda = 1.036
5 <= MAC < 10
λ = 0.979
N = 14,783
10 <= MAC < 20
λ = 1.008
N = 10,438
20 <= MAC < 50
λ = 1.02
N = 11,618
50 <= MAC < Inf
λ = 1.059
N = 59,171
−log10(observed P)
−log10(expected P)

C

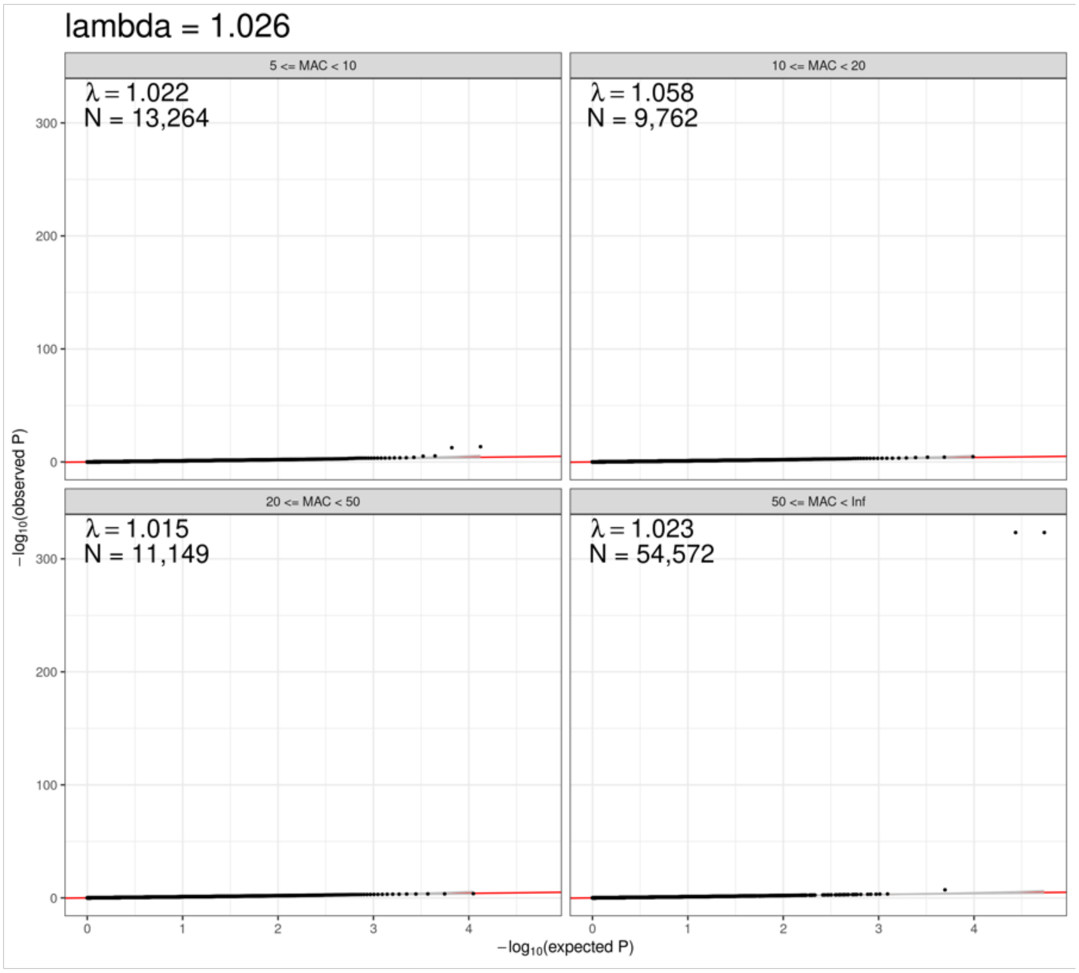
lambda = 1.026
5 <= MAC < 10
λ = 1.022
N = 13,264
10 <= MAC < 20
λ = 1.058
N = 9,762
20 <= MAC < 50
λ = 1.015
N = 11,149
50 <= MAC < Inf
λ = 1.023
N = 54,572
−log10(observed P)
−log10(expected P)

D

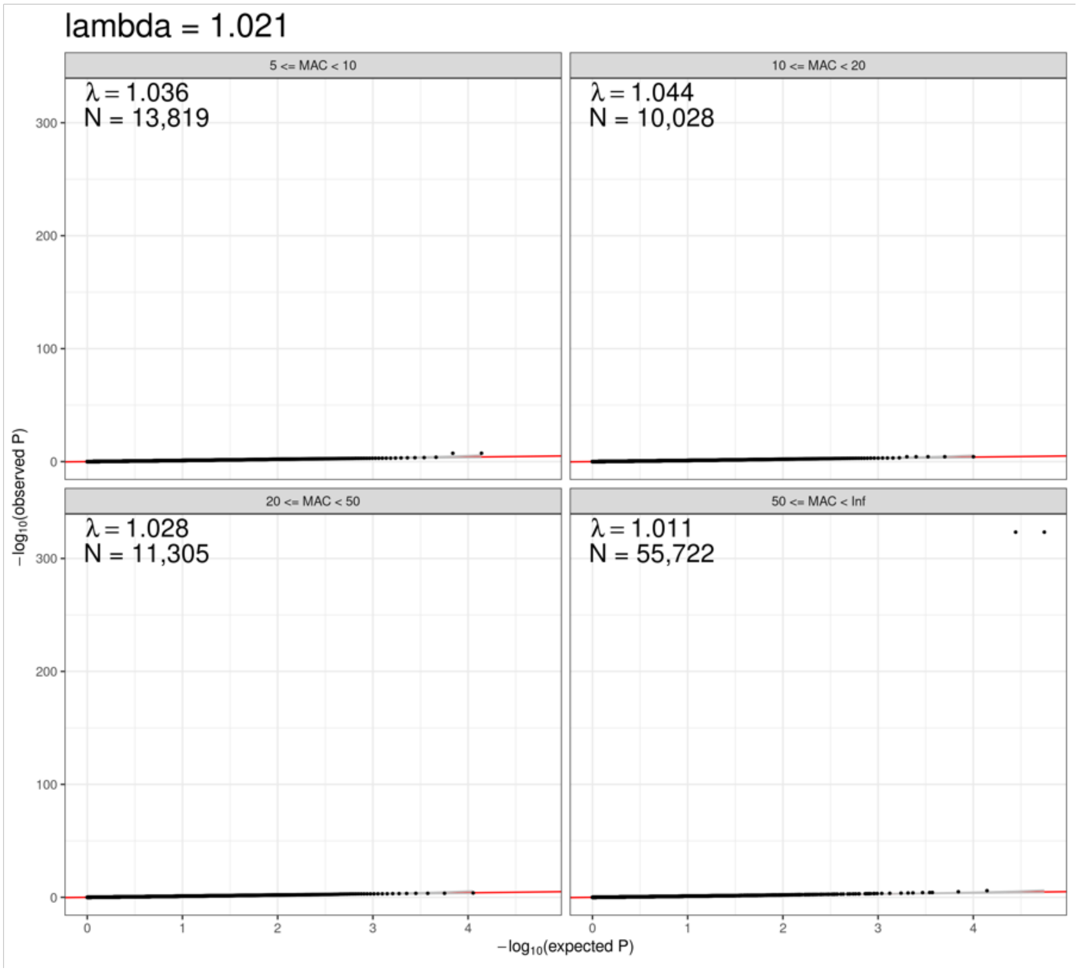
lambda = 1.021
5 <= MAC < 10
λ = 1.036
N = 13,819
10 <= MAC < 20
λ = 1.044
N = 10,028
20 <= MAC < 50
λ = 1.028
N = 11,305
50 <= MAC < Inf
λ = 1.011
N = 55,722
−log10(observed P)
−log10(expected P)

E

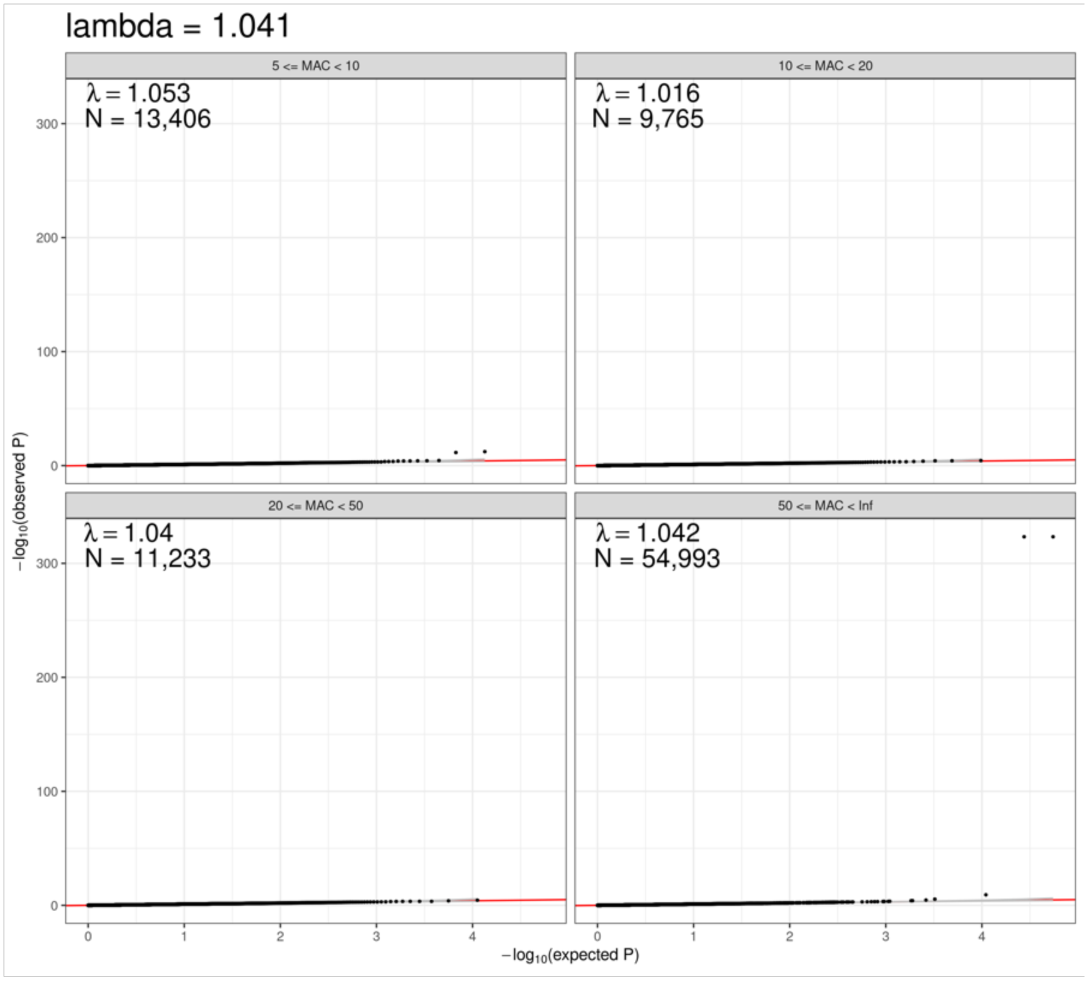

lambda = 1.041
5 <= MAC < 10
λ = 1.053
N = 13,406
10 <= MAC < 20
λ = 1.016
N = 9,765
20 <= MAC < 50
λ = 1.04
N = 11,233
50 <= MAC < Inf
λ = 1.042
N = 54,993
−log10(observed P)
−log10(expected P)

F

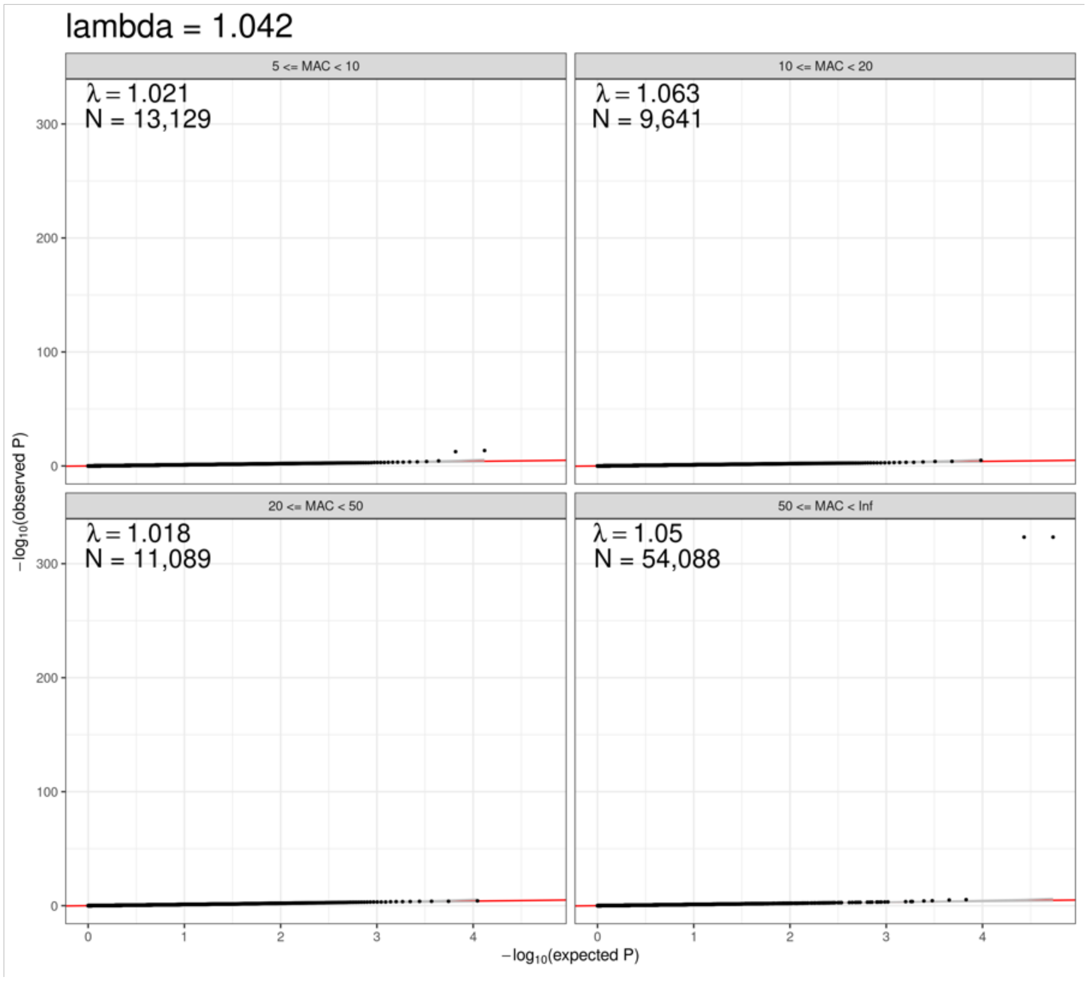

lambda = 1.042
5 <= MAC < 10
λ = 1.021
N = 13,129
10 <= MAC < 20
λ = 1.063
N = 9,641
20 <= MAC < 50
λ = 1.018
N = 11,089
50 <= MAC < Inf
λ = 1.05
N = 54,088
−log10(observed P)
−log10(expected P)

G

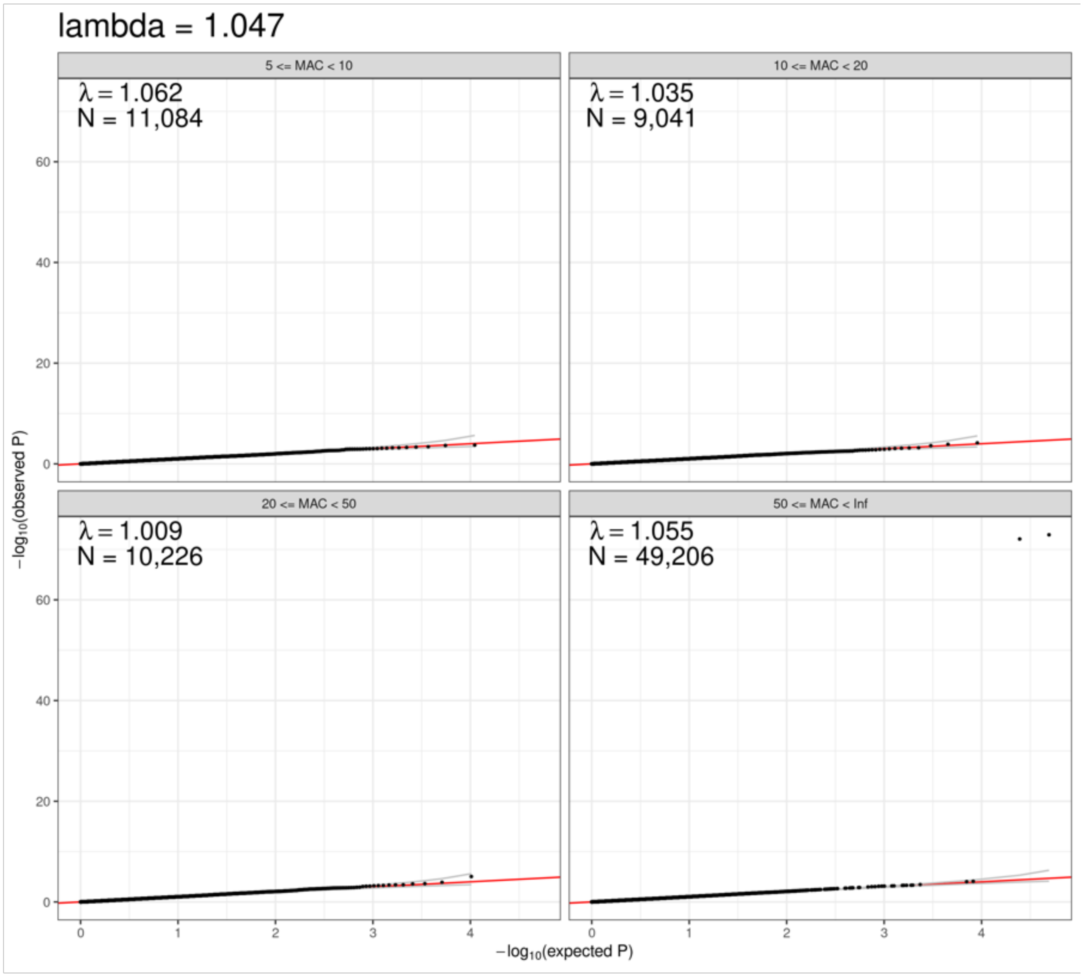
lambda = 1.047
5 <= MAC < 10
λ = 1.062
N = 11,084
10 <= MAC < 20
λ = 1.035
N = 9,041
20 <= MAC < 50
λ = 1.009
N = 10,226
50 <= MAC < Inf
λ = 1.055
N = 49,206
−log10(observed P)
−log10(expected P)

H

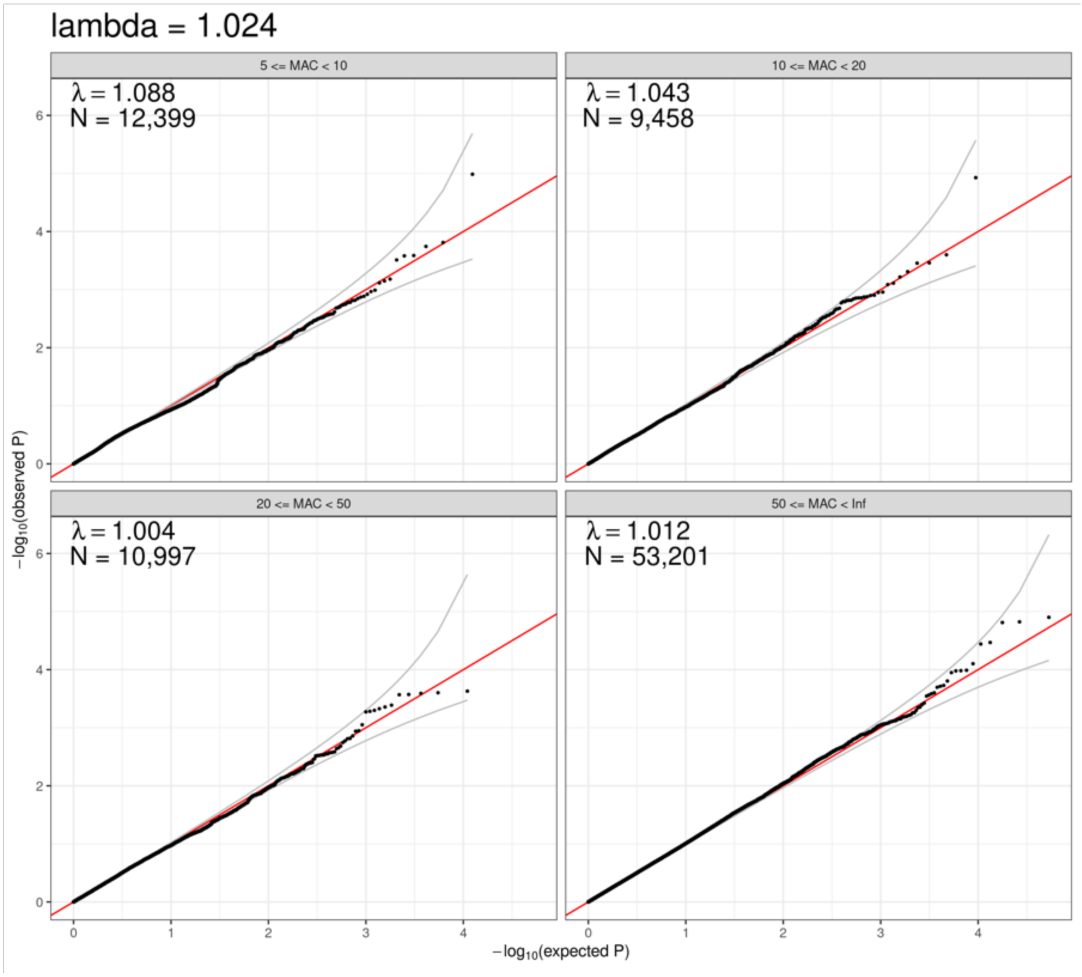
lambda = 1.024
5 <= MAC < 10
λ = 1.088
N = 12,399
10 <= MAC < 20
λ = 1.043
N = 9,458
20 <= MAC < 50
λ = 1.004
N = 10,997
50 <= MAC < Inf
λ = 1.012
N = 53,201
−log10(observed P)
−log10(expected P)

I

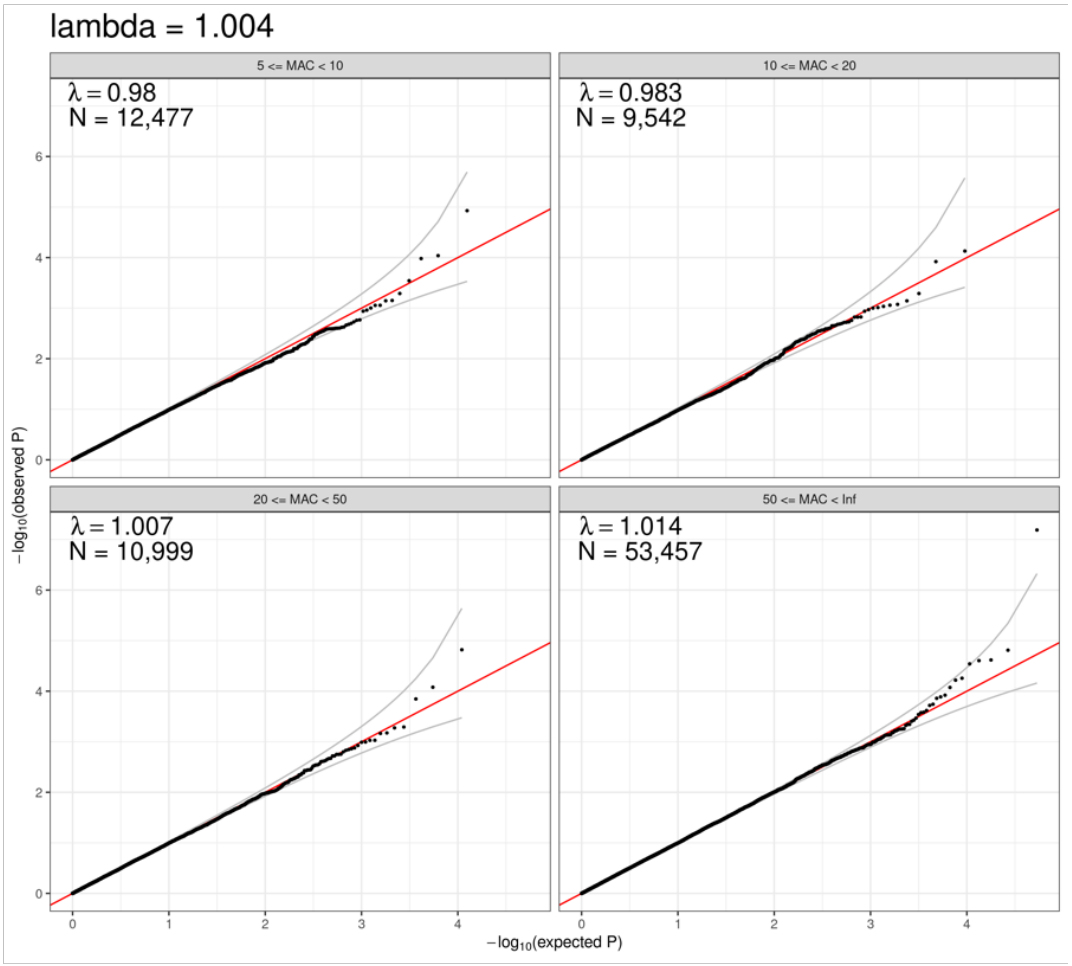
lambda = 1.004
5 <= MAC < 10
λ = 0.98
N = 12,477
10 <= MAC < 20
λ = 0.983
N = 9,542
20 <= MAC < 50
λ = 1.007
N = 10,999
50 <= MAC < Inf
λ = 1.014
N = 53,457
−log10(observed P)
−log10(expected P)

J

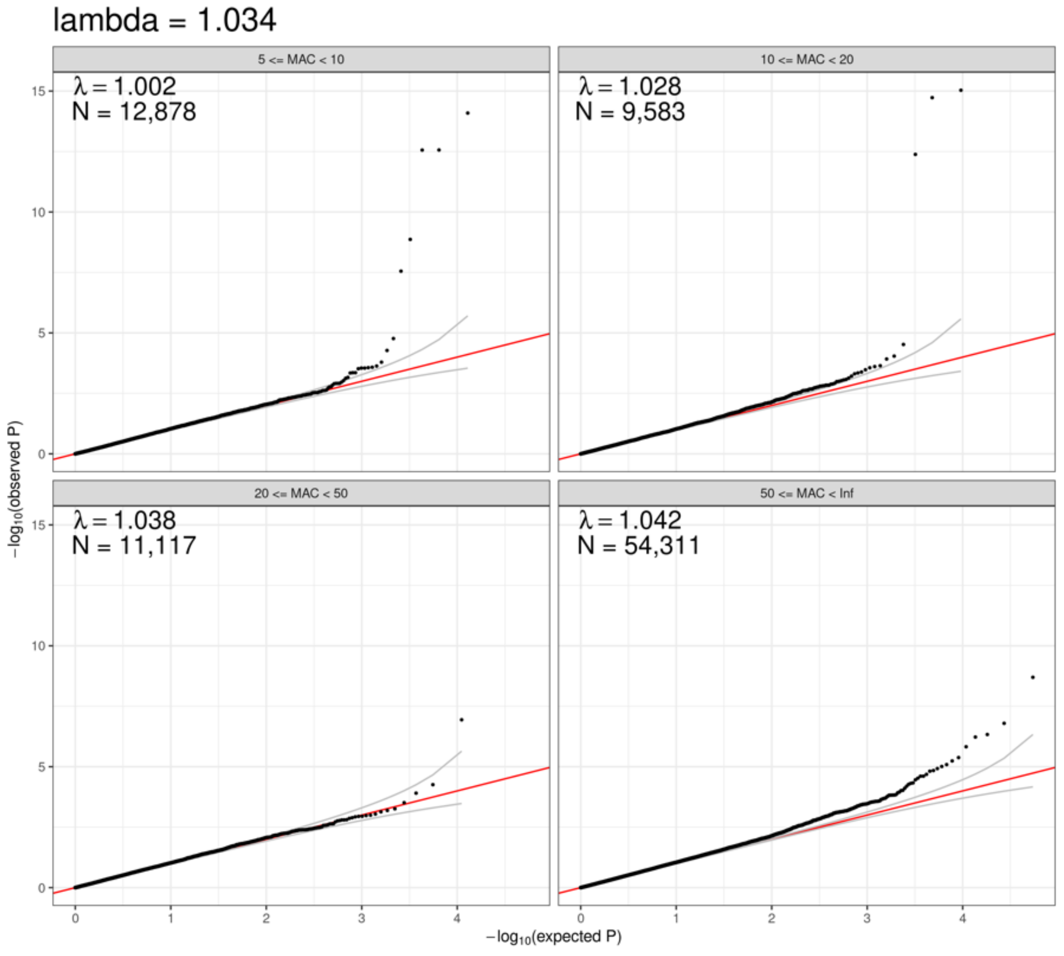
lambda = 1.034
5 <= MAC < 10
λ = 1.002
N = 12,878
10 <= MAC < 20
λ = 1.028
N = 9,583
20 <= MAC < 50
λ = 1.038
N = 11,117
50 <= MAC < Inf
λ = 1.042
N = 54,311
−log10(observed P)
−log10(expected P)

K

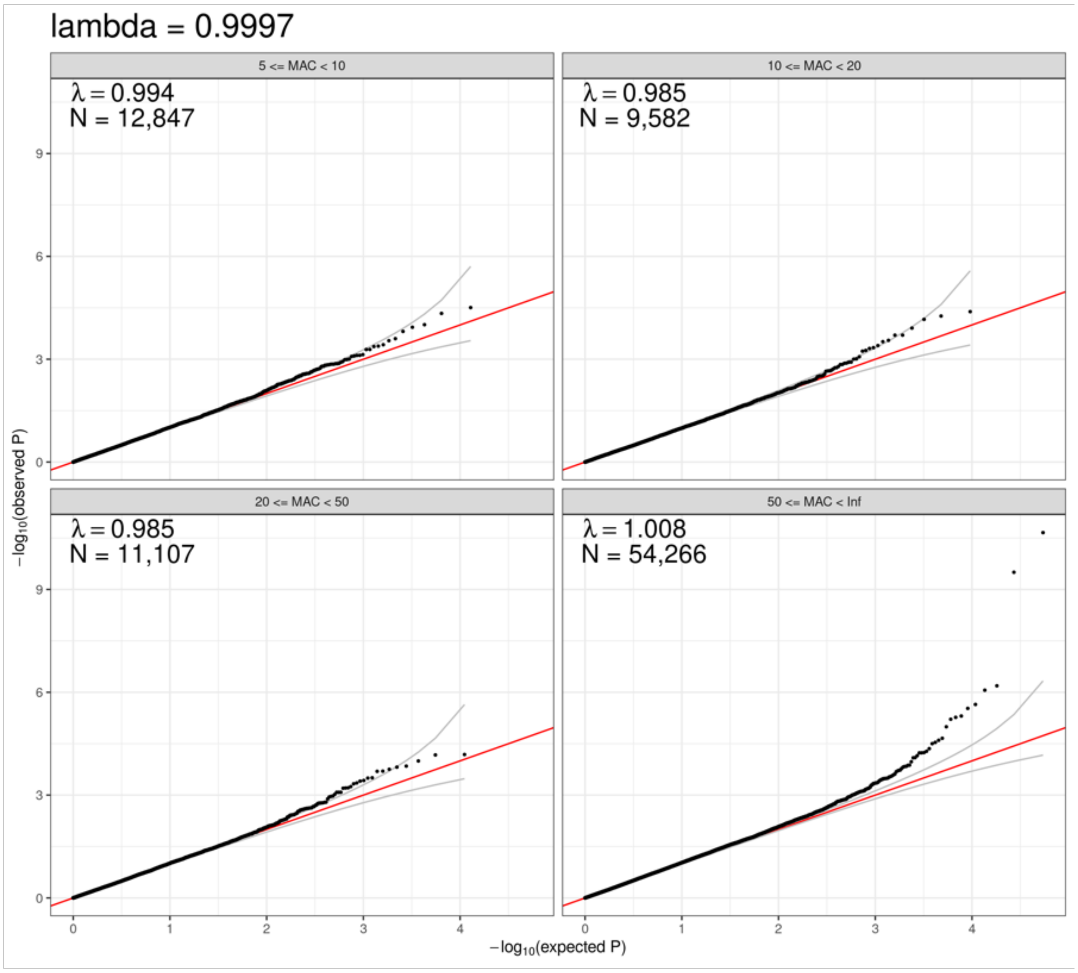

lambda = 0.9997
5 <= MAC < 10
λ = 0.994
N = 12,847
10 <= MAC < 20
λ = 0.985
N = 9,582
20 <= MAC < 50
λ = 0.985
N = 11,107
50 <= MAC < Inf
λ = 1.008
N = 54,266
−log10(observed P)
−log10(expected P)

L

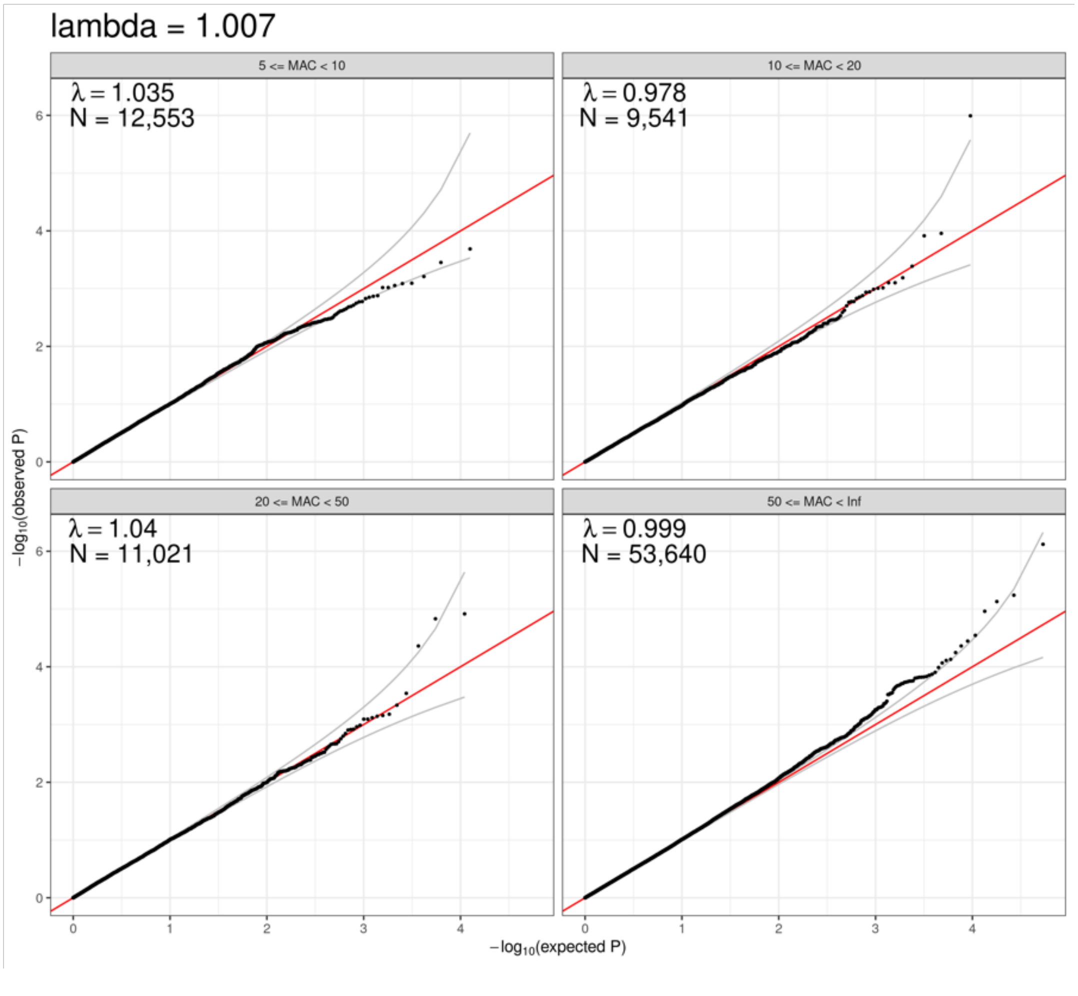

lambda = 1.007
5 <= MAC < 10
λ = 1.035
N = 12,553
10 <= MAC < 20
λ = 0.978
N = 9,541
20 <= MAC < 50
λ = 1.04
N = 11,021
50 <= MAC < Inf
λ = 0.999
N = 53,640
−log10(observed P)
−log10(expected P)

M

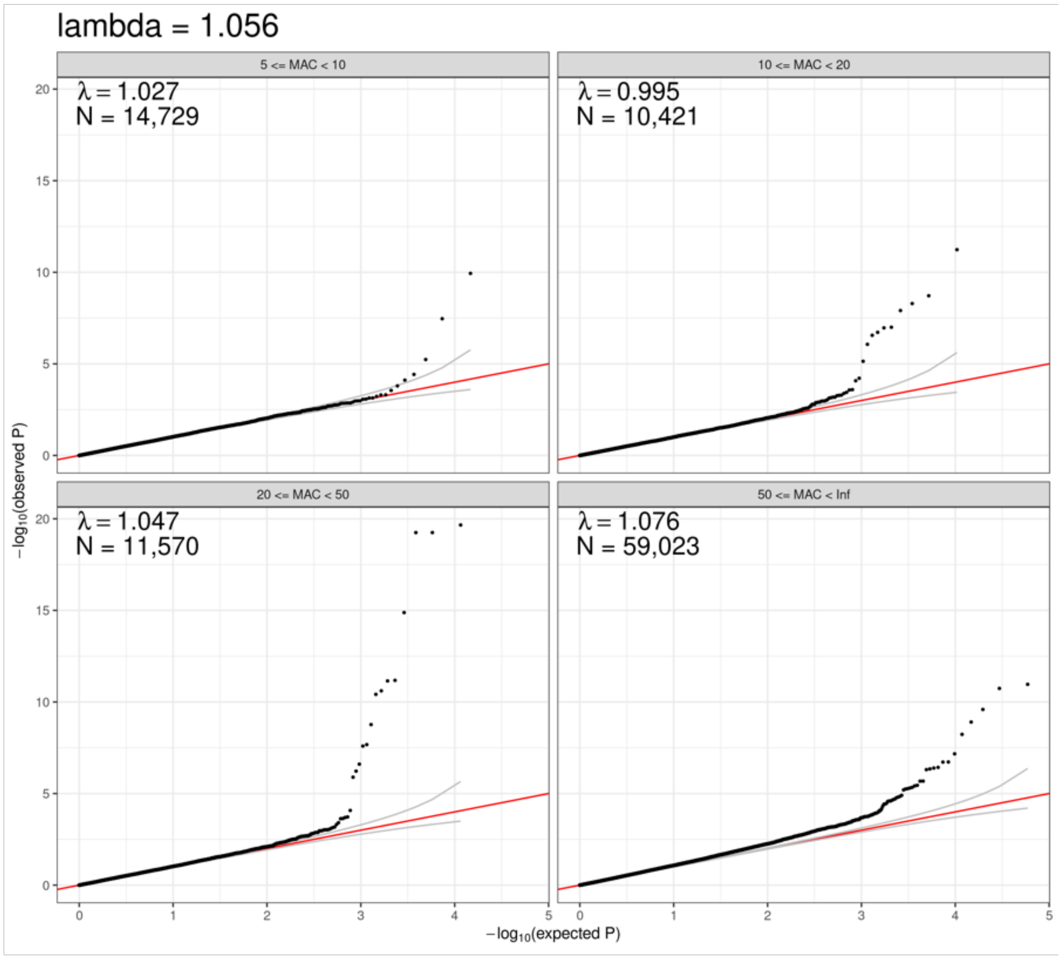
lambda = 1.056
5 <= MAC < 10
λ = 1.027
N = 14,729
10 <= MAC < 20
λ = 0.995
N = 10,421
20 <= MAC < 50
λ = 1.047
N = 11,570
50 <= MAC < Inf
λ = 1.076
N = 59,023
−log10(observed P)
−log10(expected P)

N

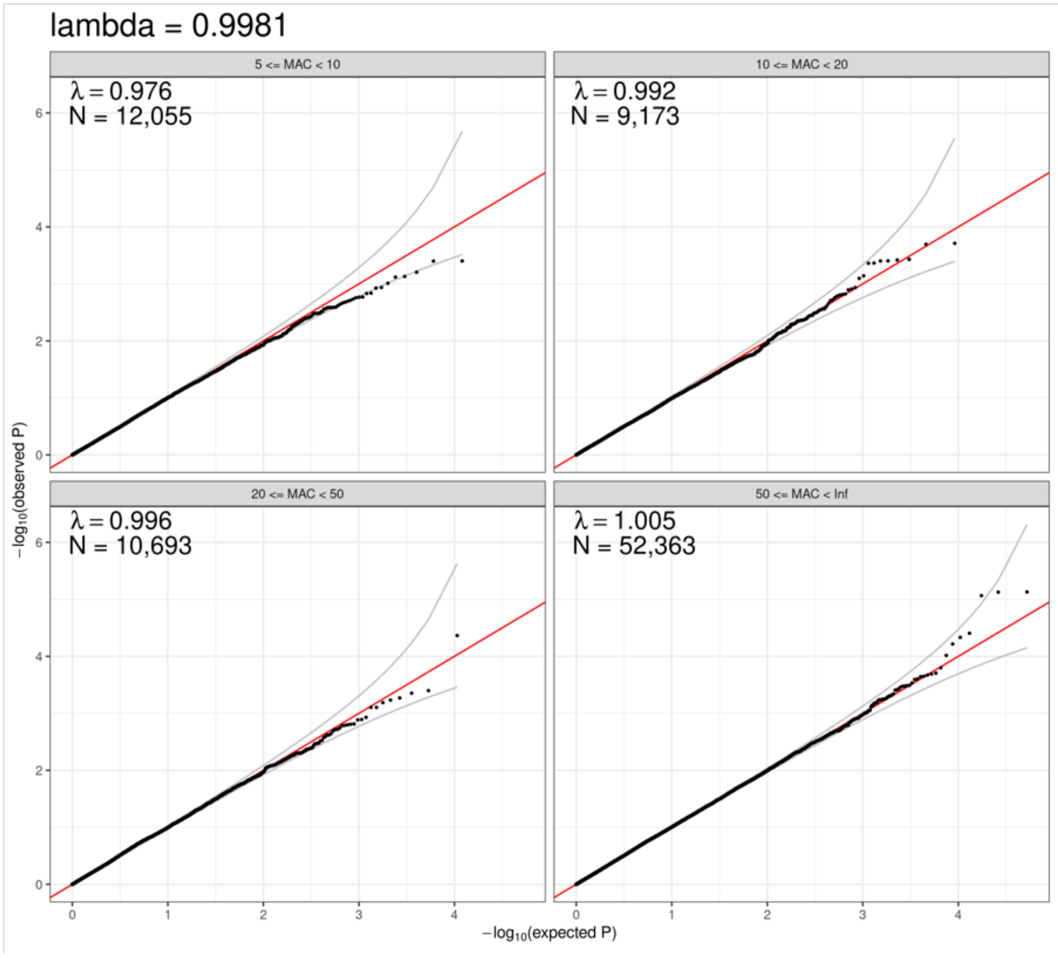
lambda = 0.9981
5 <= MAC < 10
λ = 0.976
N = 12,055
10 <= MAC < 20
λ = 0.992
N = 9,173
20 <= MAC < 50
λ = 0.996
N = 10,693
50 <= MAC < Inf
λ = 1.005
N = 52,363
−log10(observed P)
−log10(expected P)

O

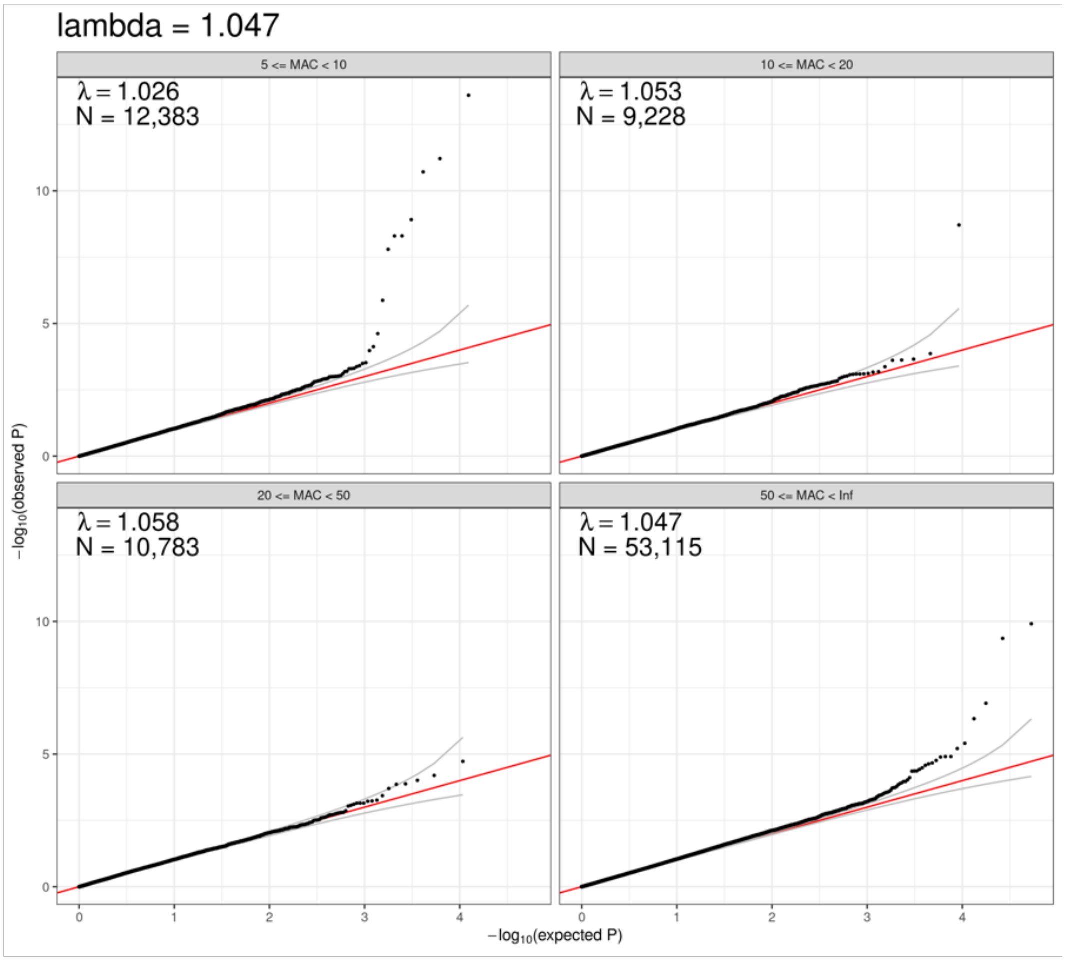

lambda = 1.047
5 <= MAC < 10
λ = 1.026
N = 12,383
10 <= MAC < 20
λ = 1.053
N = 9,228
20 <= MAC < 50
λ = 1.058
N = 10,783
50 <= MAC < Inf
λ = 1.047
N = 53,115
−log10(observed P)
−log10(expected P)

P

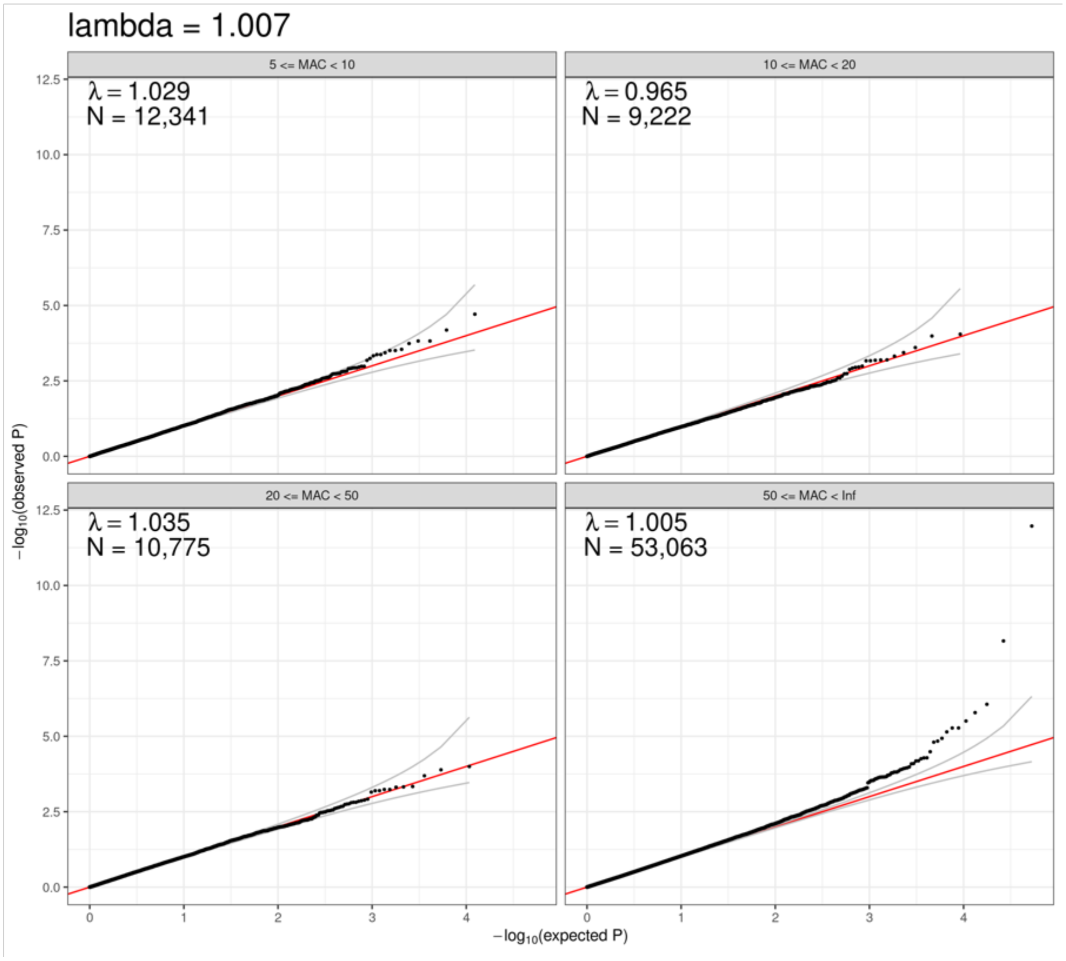

lambda = 1.007
5 <= MAC < 10
λ = 1.029
N = 12,341
10 <= MAC < 20
λ = 0.965
N = 9,222
20 <= MAC < 50
λ = 1.035
N = 10,775
50 <= MAC < Inf
λ = 1.005
N = 53,063
−log10(observed P)
−log10(expected P)

Q

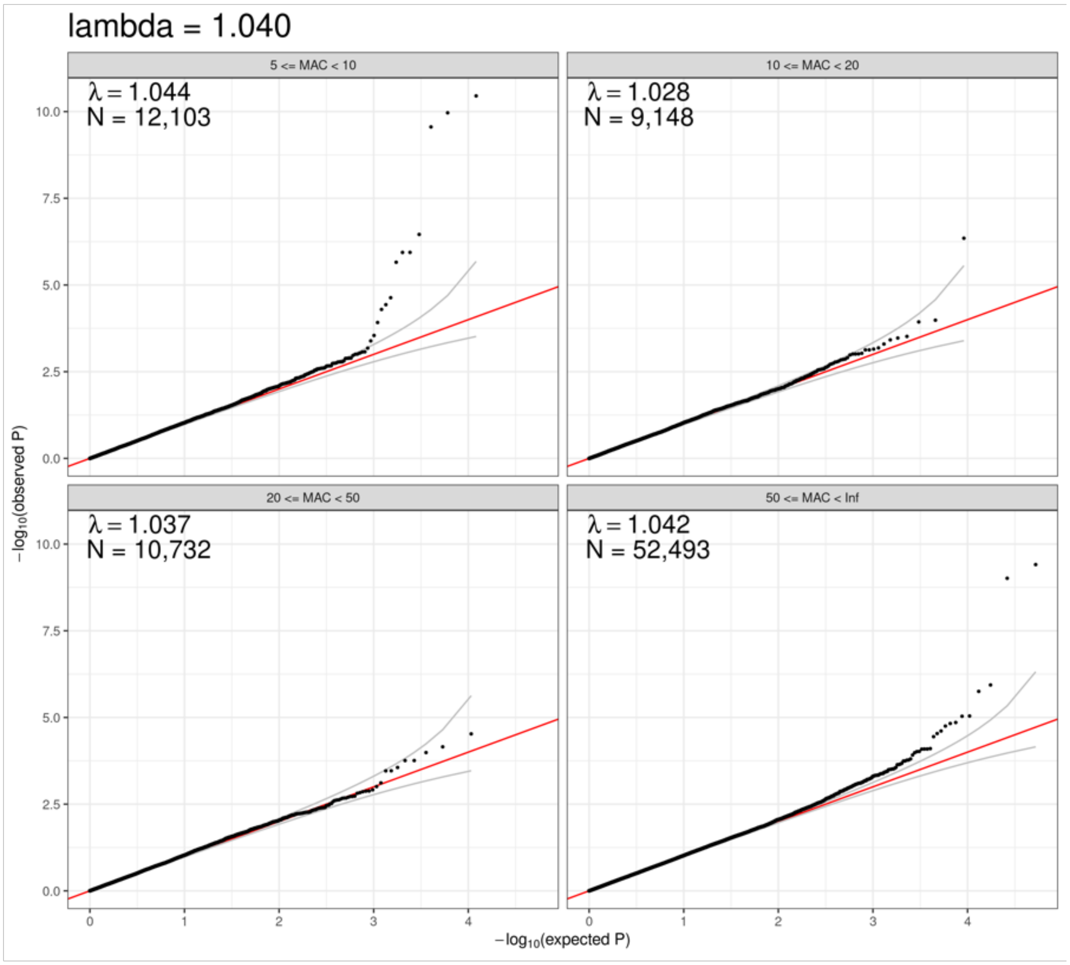
lambda = 1.040
5 <= MAC < 10
λ = 1.044
N = 12,103
10 <= MAC < 20
λ = 1.028
N = 9,148
20 <= MAC < 50
λ = 1.037
N = 10,732
50 <= MAC < Inf
λ = 1.042
N = 52,493
−log10(observed P)
−log10(expected P)

R

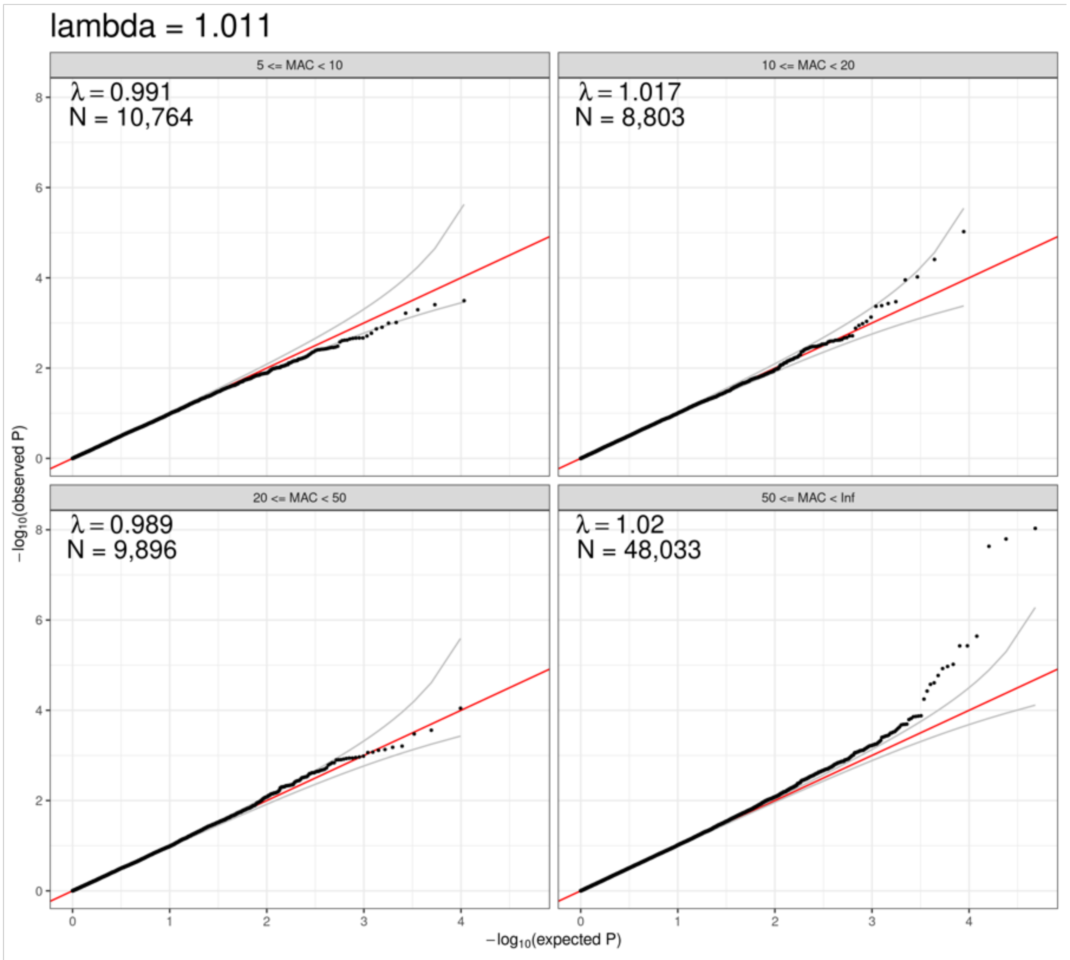
lambda = 1.011
5 <= MAC < 10
λ = 0.991
N = 10,764
10 <= MAC < 20
λ = 1.017
N = 8,803
20 <= MAC < 50
λ = 0.989
N = 9,896
50 <= MAC < Inf
λ = 1.02
N = 48,033
−log10(observed P)
−log10(expected P)

S

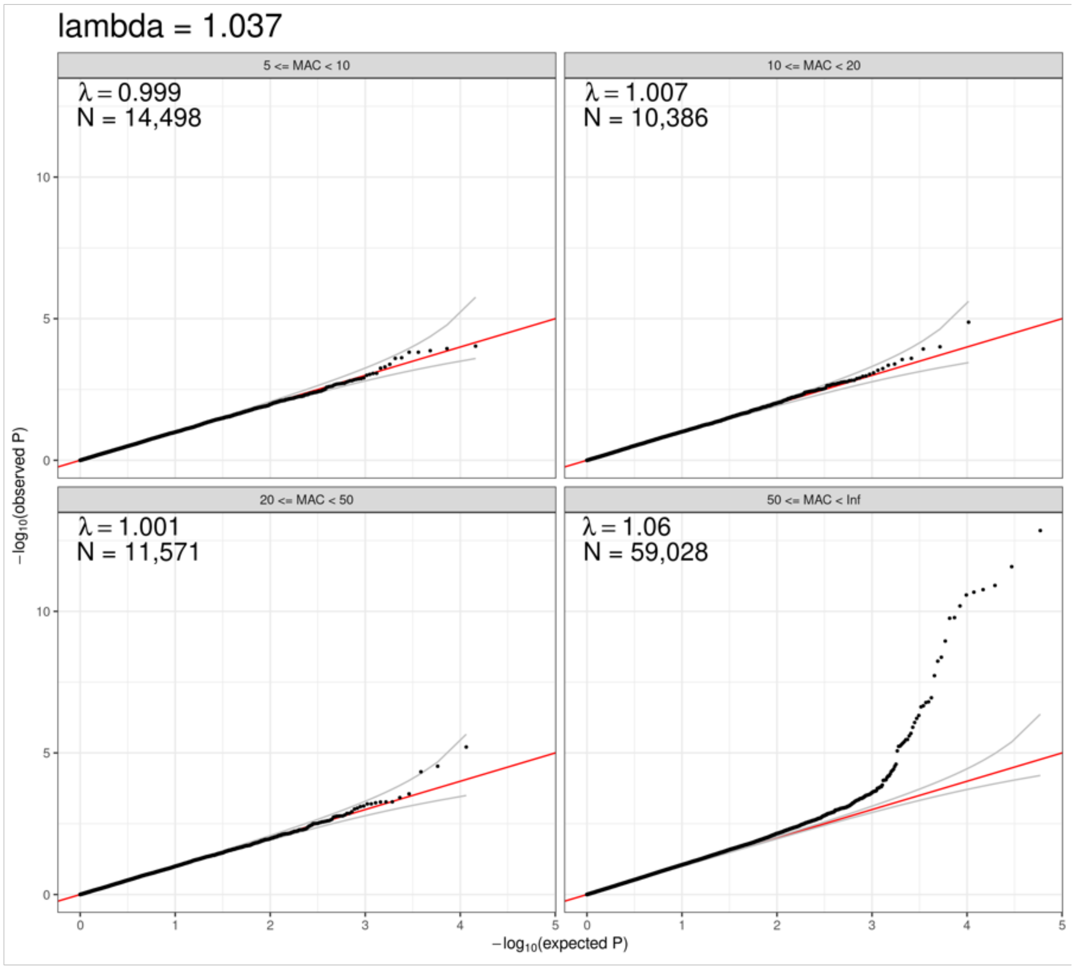

lambda = 1.037
5 <= MAC < 10
λ = 0.999
N = 14,498
10 <= MAC < 20
λ = 1.007
N = 10,386
20 <= MAC < 50
λ = 1.001
N = 11,571
50 <= MAC < Inf
λ = 1.06
N = 59,028
−log10(observed P)
−log10(expected P)

T

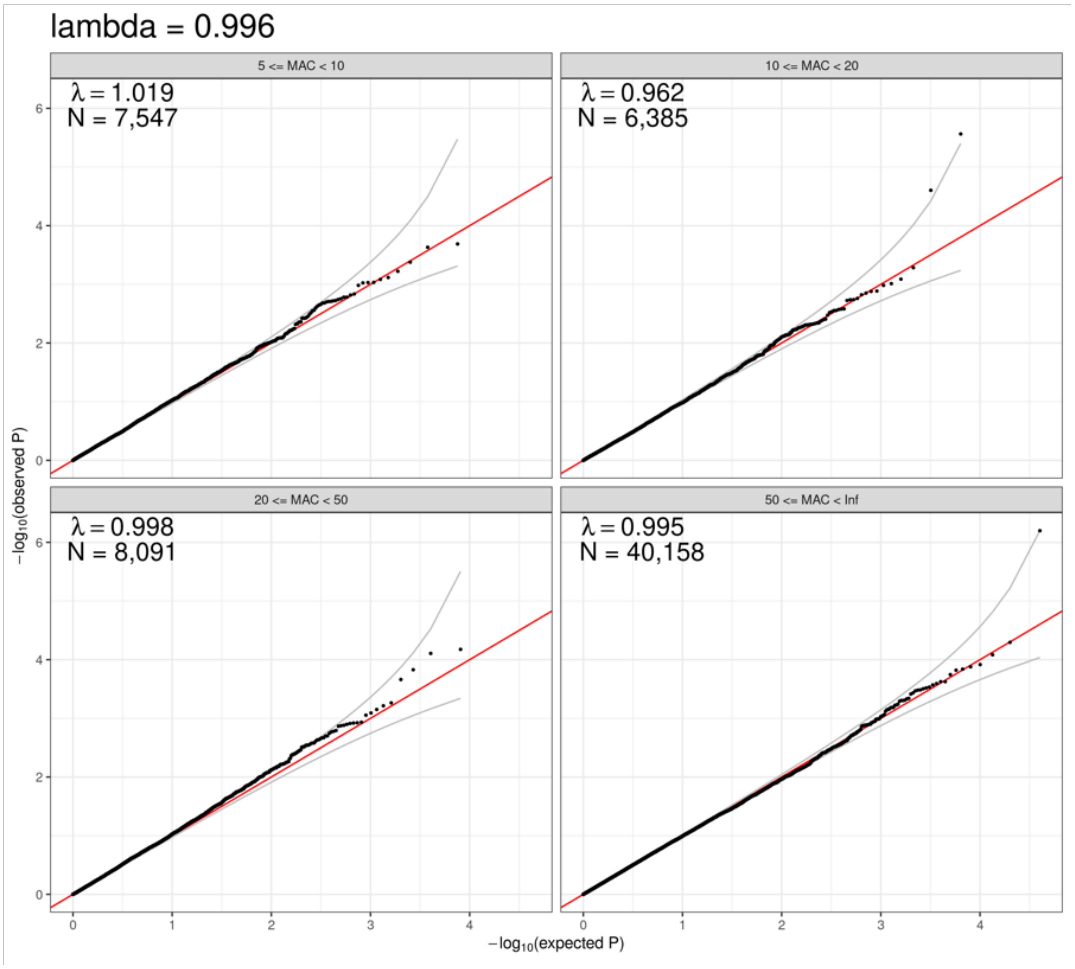

lambda = 0.996
5 <= MAC < 10
λ = 1.019
N = 7,547
10 <= MAC < 20
λ = 0.962
N = 6,385
20 <= MAC < 50
λ = 0.998
N = 8,091
50 <= MAC < Inf
λ = 0.995
N = 40,158
−log10(observed P)
−log10(expected P)

U

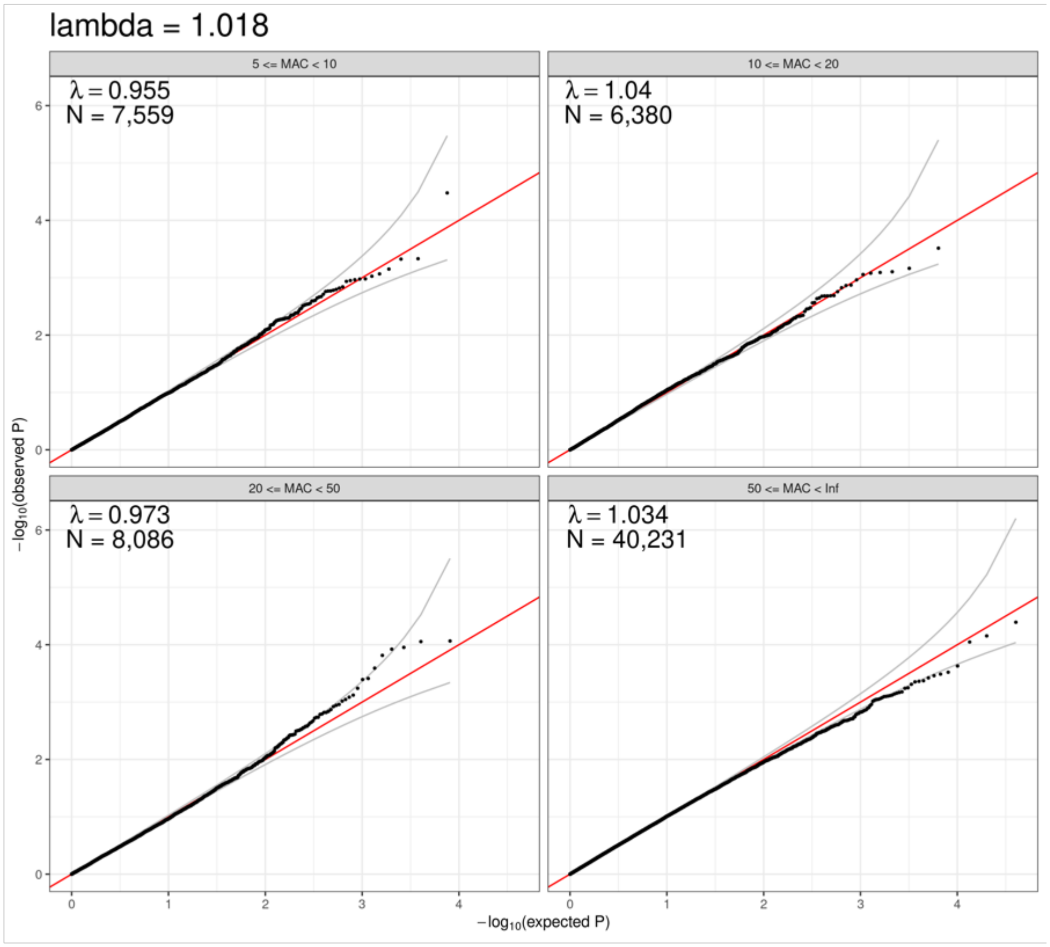
lambda = 1.018
5 <= MAC < 10
λ = 0.955
N = 7,559
10 <= MAC < 20
λ = 1.04
N = 6,380
20 <= MAC < 50
λ = 0.973
N = 8,086
50 <= MAC < Inf
λ = 1.034
N = 40,231
−log10(observed P)
−log10(expected P)

V

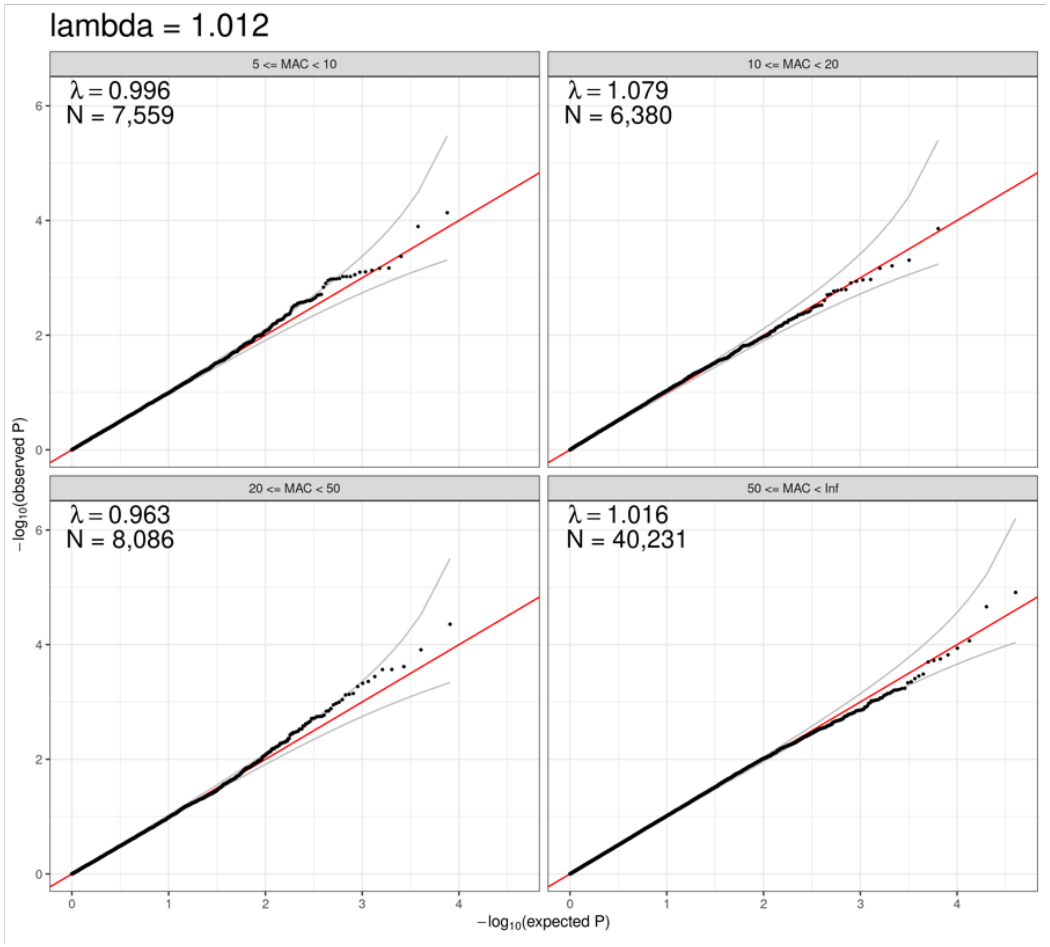
lambda = 1.012
5 <= MAC < 10
λ = 0.996
N = 7,559
10 <= MAC < 20
λ = 1.079
N = 6,380
20 <= MAC < 50
λ = 0.963
N = 8,086
50 <= MAC < Inf
λ = 1.016
N = 40,231
−log10(observed P)
−log10(expected P)

W

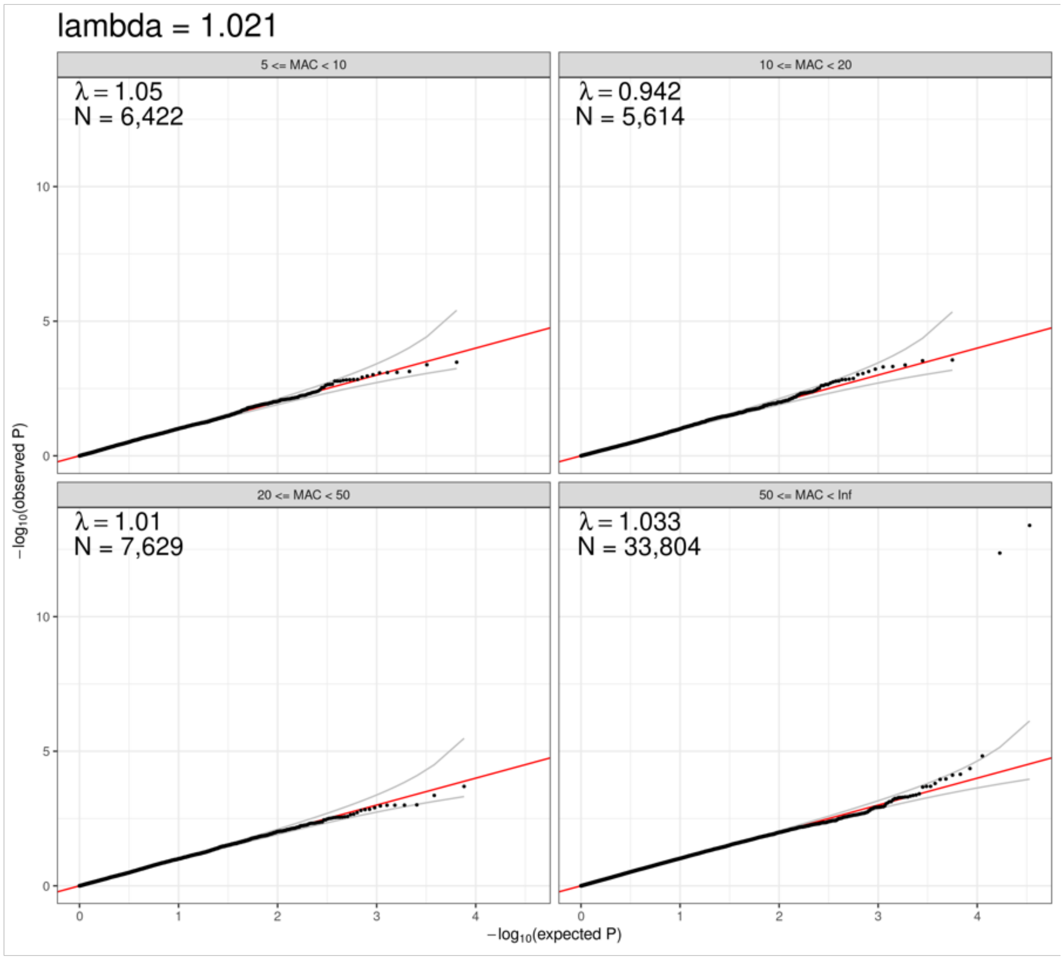

lambda = 1.021
5 <= MAC < 10
λ = 1.05
N = 6,422
10 <= MAC < 20
λ = 0.942
N = 5,614
20 <= MAC < 50
λ = 1.01
N = 7,629
50 <= MAC < Inf
λ = 1.033
N = 33,804
−log10(observed P)
−log10(expected P)

X

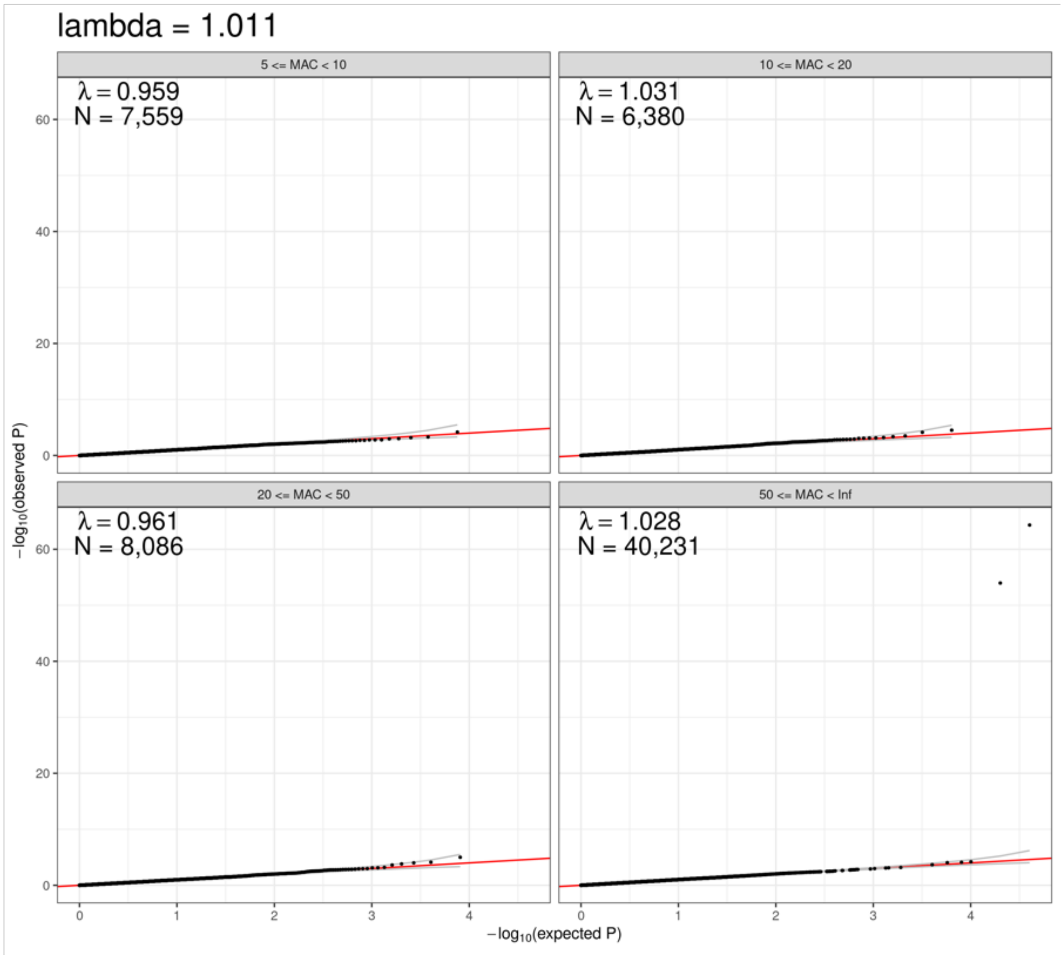

lambda = 1.011
5 <= MAC < 10
λ = 0.959
N = 7,559
10 <= MAC < 20
λ = 1.031
N = 6,380
20 <= MAC < 50
λ = 0.961
N = 8,086
50 <= MAC < Inf
λ = 1.028
N = 40,231
−log10(observed P)
−log10(expected P)

Figure S3. Manhattan plots of the marginal structural variant analyses in TOPMed. All p-values are derived from two-sided t-tests and are not adjusted for multiple comparisons. (A) HCT; (B) HGB; (C) MCH; (D) MCHC; (E) MCV; (F) RBC; (G) RDW; (H) BASO_binary; (I) EOSIN; (J) LYMPHS; (K) MONOS; (L) NEUTRO; (M) WBC; (N) EOSIN%; (O) LYMPHS%; (P) MONOS%; (Q) NEUTRO%; (R) MPV; (S) PLT; (T) FERRITIN; (U) IRON; (V) SAT; (W) UIBC; (X) TIBC.

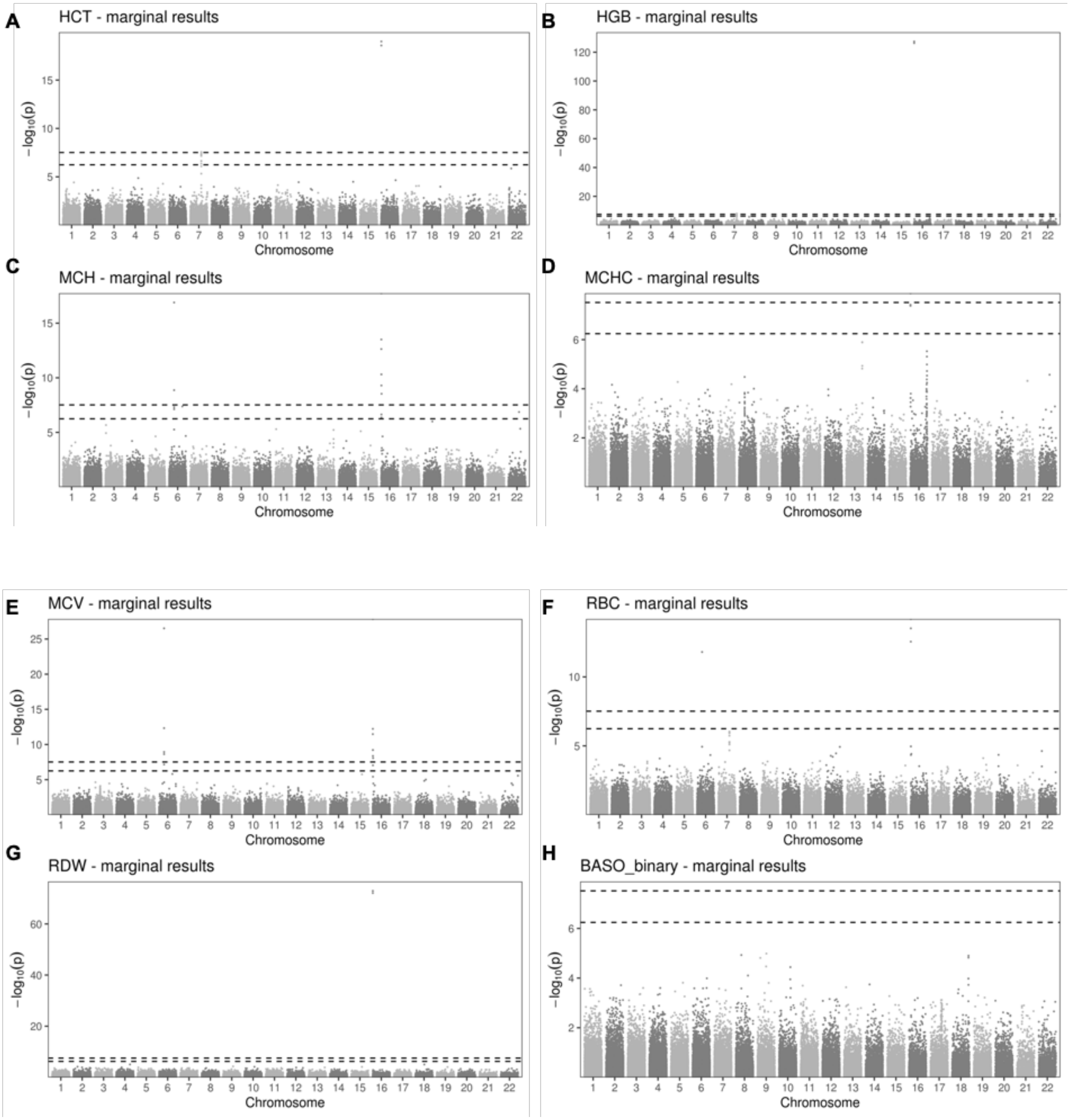

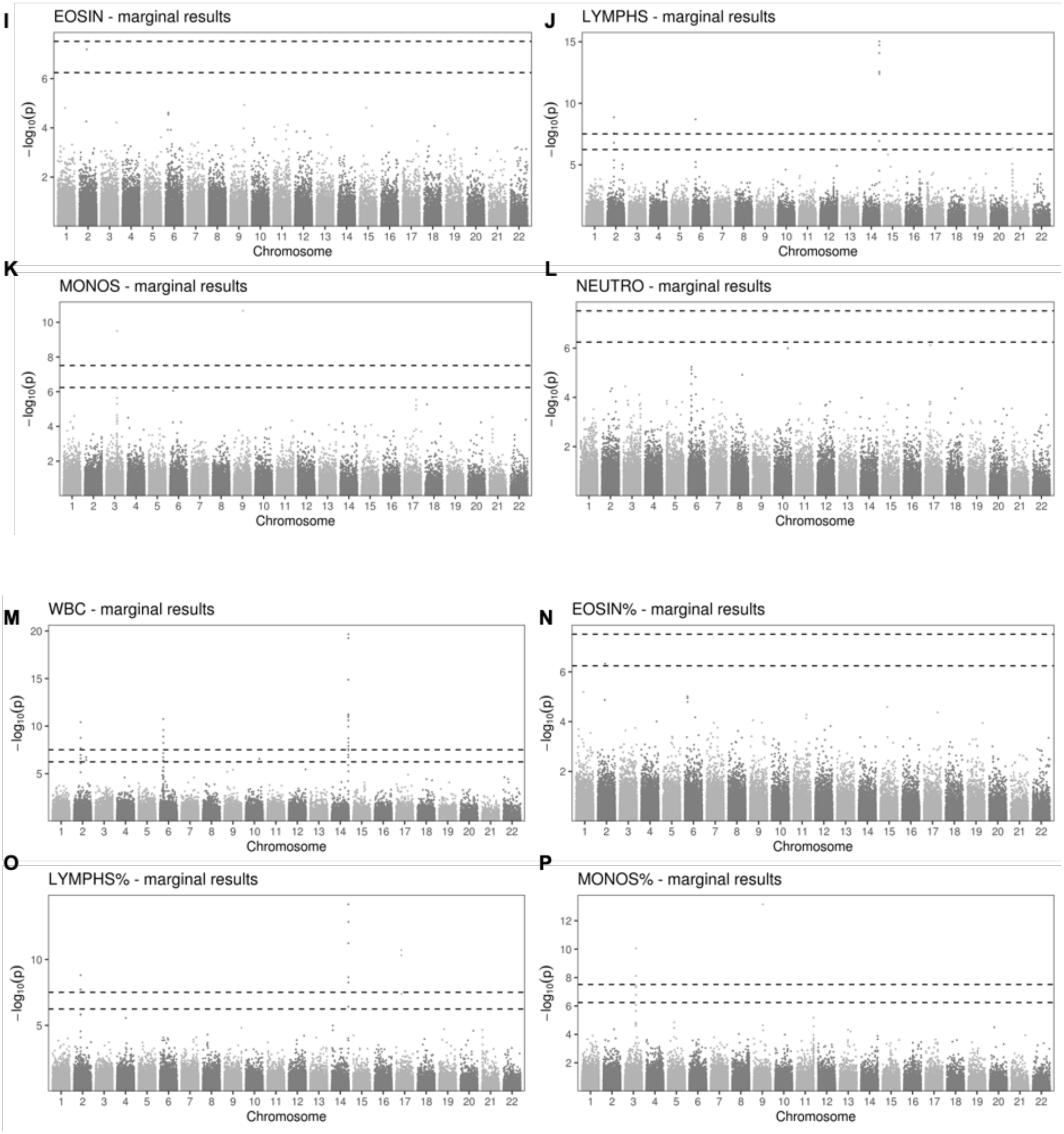
I
EOSIN - marginal results
J
LYMPHS - marginal results
K
MONOS - marginal results
L
NEUTRO - marginal results
M
WBC - marginal results
N
EOSIN% - marginal results
O
LYMPHS% - marginal results
P
MONOS% - marginal results
−log10(p)
Chromosome
1 2 3 4 5 6 7 8 9 10 11 12 13 14 15 16 17 18 19 20 21 22

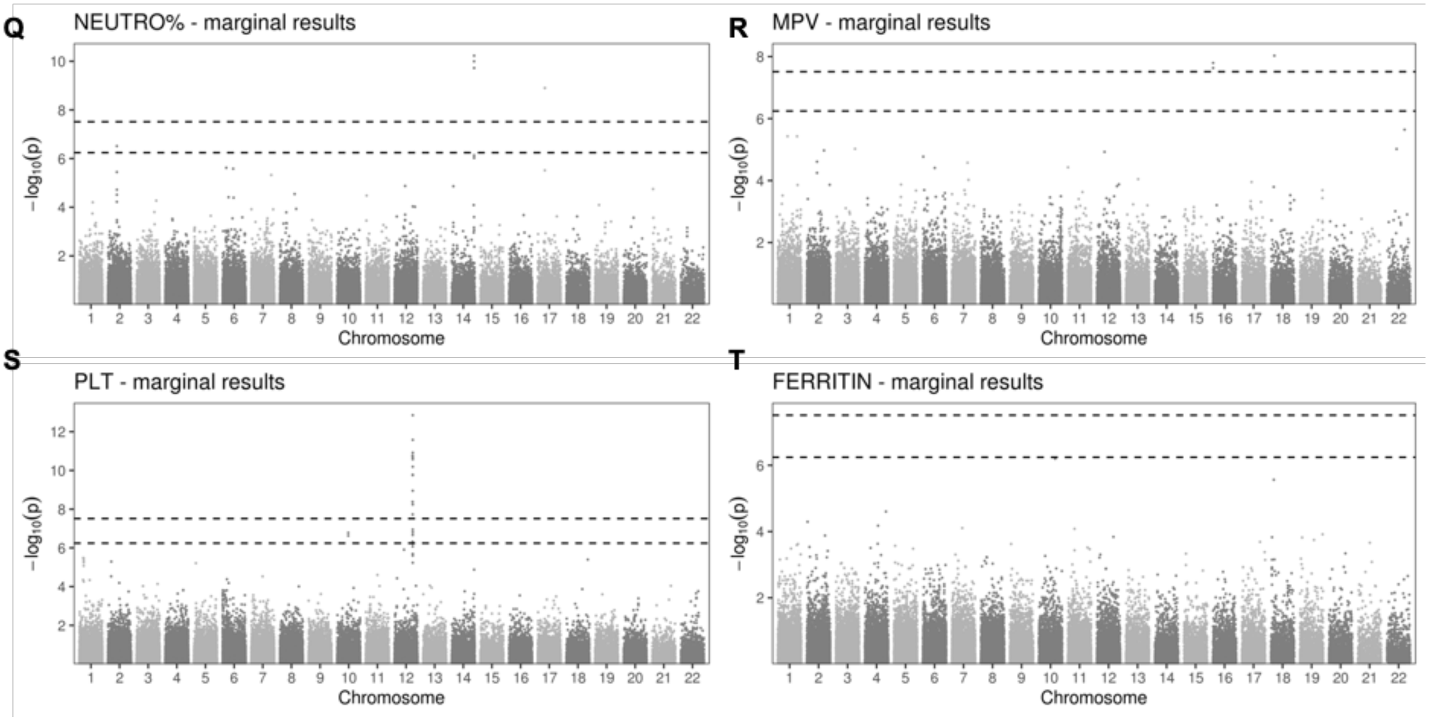
Q
NEUTRO% - marginal results
R
MPV - marginal results
S
PLT - marginal results
T
FERRITIN - marginal results
−log10(p)
Chromosome

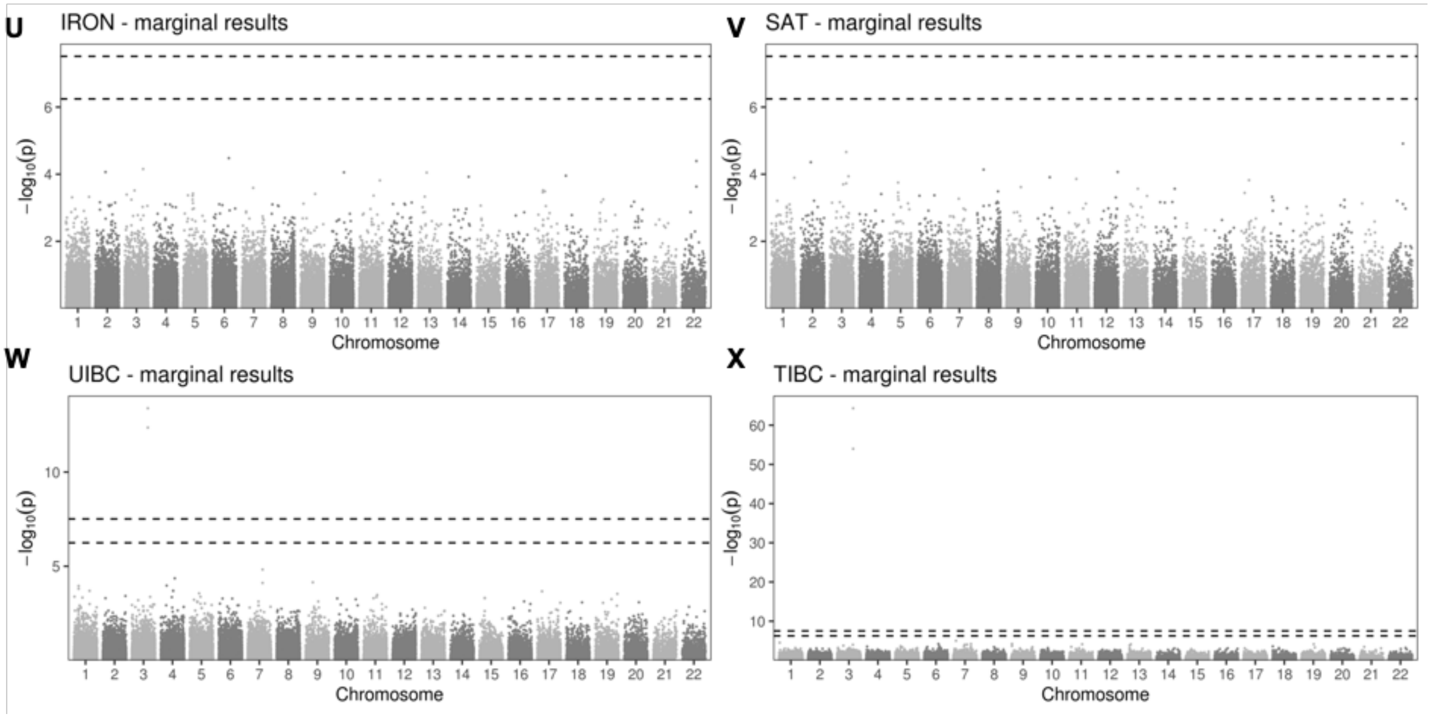
U
IRON - marginal results
V
SAT - marginal results
W
UIBC - marginal results
X
TIBC - marginal results
−log10(p)
Chromosome

Figure S4. Locus zoom plots for structural variants significantly associated with hematological and hemostasis traits in TOPMed. SVs are shown as large circles and SNVs are shown as small circles. All p-values are derived from two-sided t-tests and are not adjusted for multiple comparisons. A) LYMPH, B) LYMPH PROP, C) MONOS, D) MONOS PROP, E) NEUTRO PROP, F) WBC, G) HCT, H) HGB, I) MCH, J) MCHC, K) MCV, L) RBC, M) RDW, N), MPV, O) PLT, P) TIBC, Q) UIBC

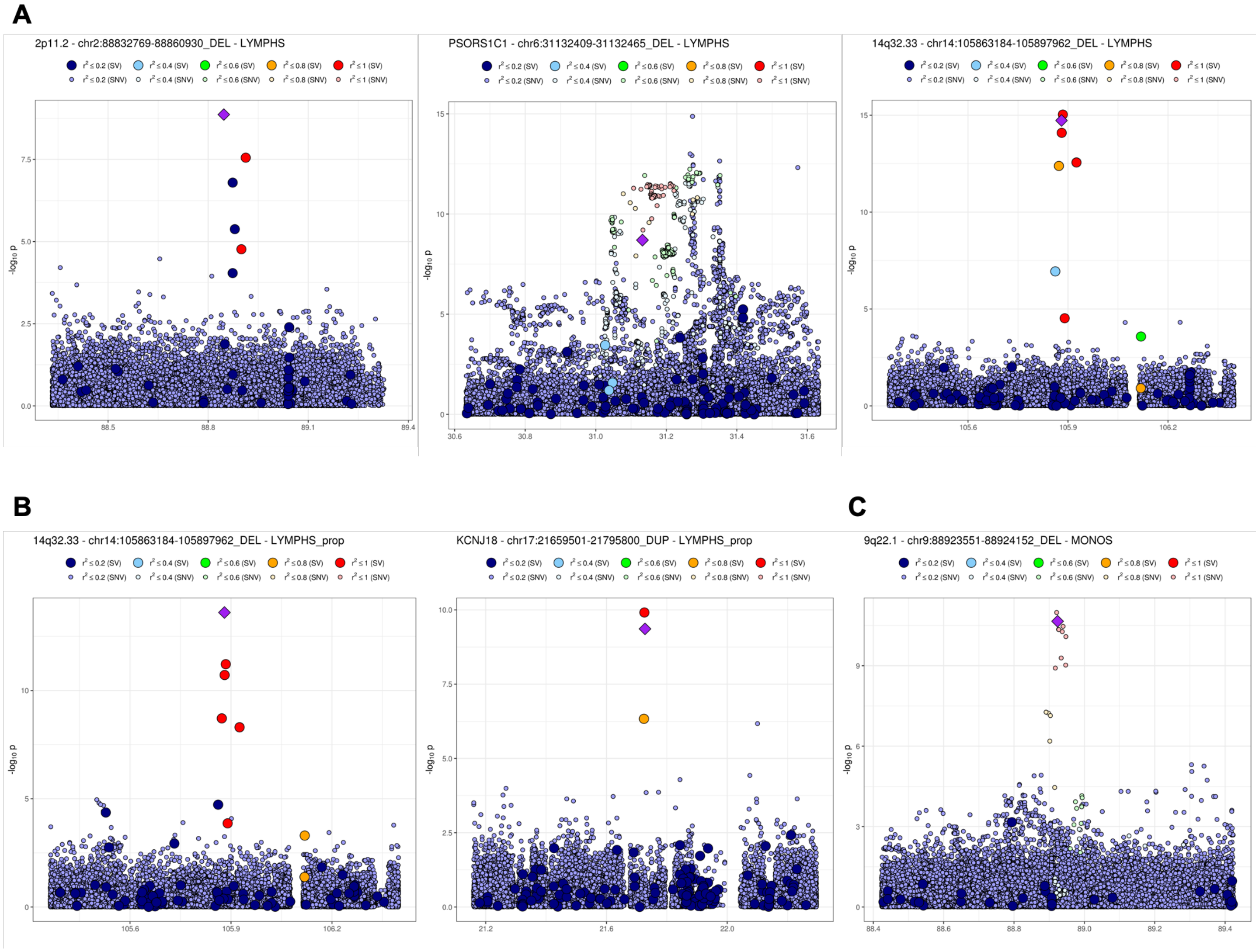

D

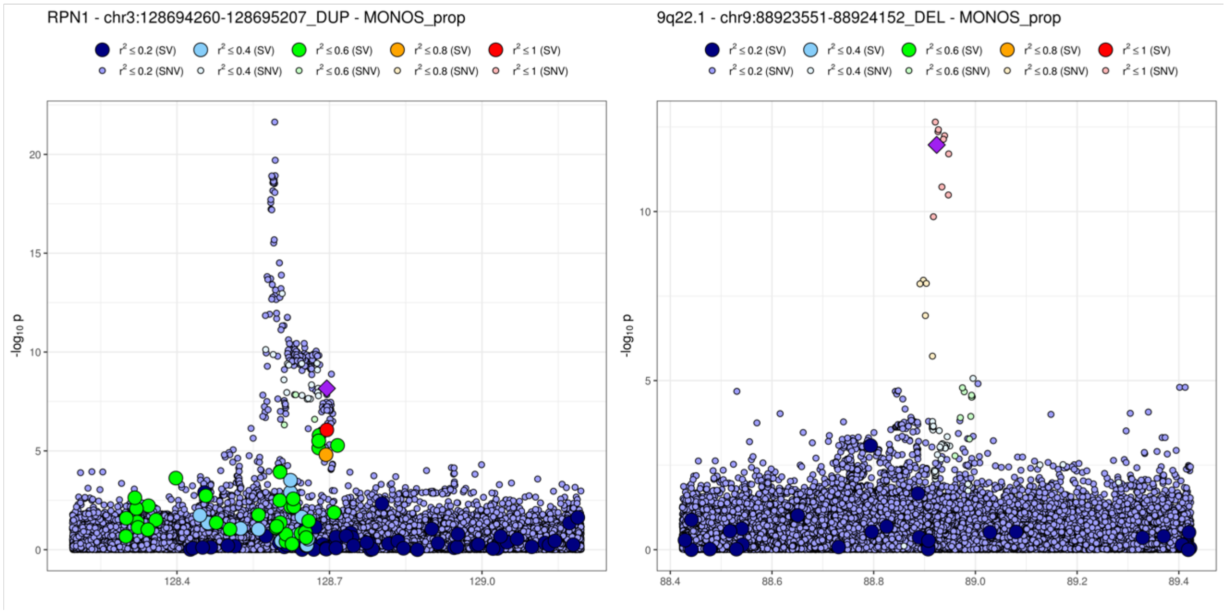

RPN1 - chr3:128694260-128695207_DUP - MONOS_prop
9q22.1 - chr9:88923551-88924152_DEL - MONOS_prop
r² ≤ 0.2 (SV)
r² ≤ 0.4 (SV)
r² ≤ 0.6 (SV)
r² ≤ 0.8 (SV)
r² ≤ 1 (SV)
r² ≤ 0.2 (SNV)
r² ≤ 0.4 (SNV)
r² ≤ 0.6 (SNV)
r² ≤ 0.8 (SNV)
r² ≤ 1 (SNV)
-log10 p
0
5
10
15
20
128.4
128.7
129.0
88.4
88.6
88.8
89.0
89.2
89.4

E

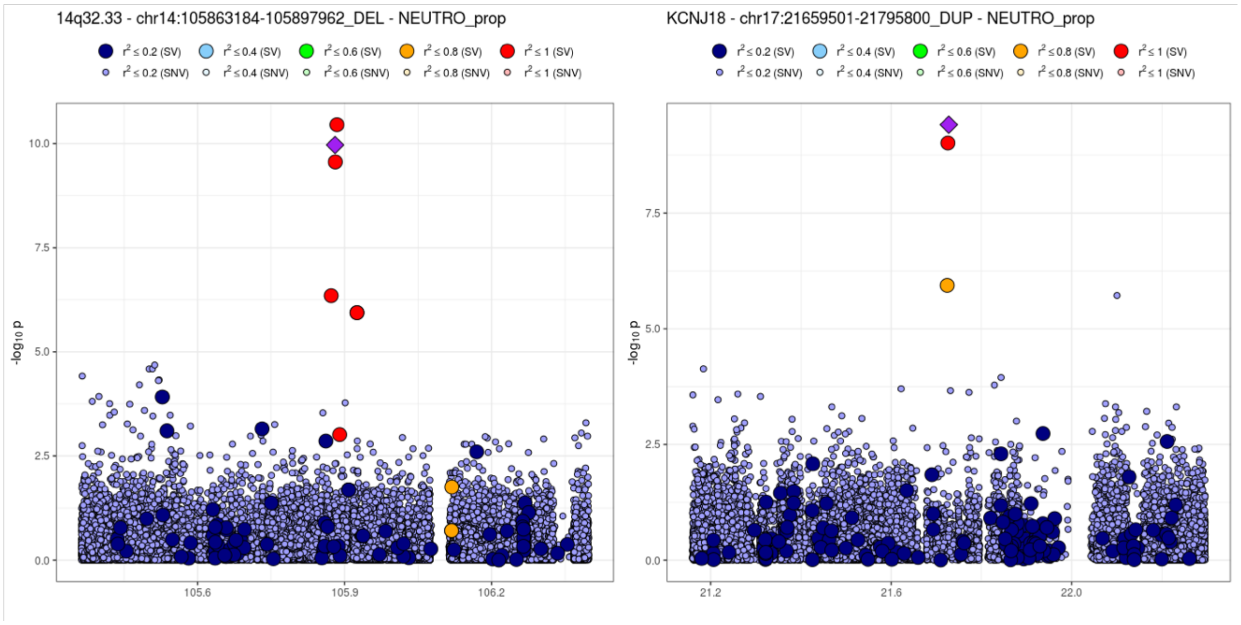

14q32.33 - chr14:105863184-105897962_DEL - NEUTRO_prop
KCNJ18 - chr17:21659501-21795800_DUP - NEUTRO_prop
r² ≤ 0.2 (SV)
r² ≤ 0.4 (SV)
r² ≤ 0.6 (SV)
r² ≤ 0.8 (SV)
r² ≤ 1 (SV)
r² ≤ 0.2 (SNV)
r² ≤ 0.4 (SNV)
r² ≤ 0.6 (SNV)
r² ≤ 0.8 (SNV)
r² ≤ 1 (SNV)
-log10 p
0.0
2.5
5.0
7.5
10.0
105.6
105.9
106.2
21.2
21.6
22.0

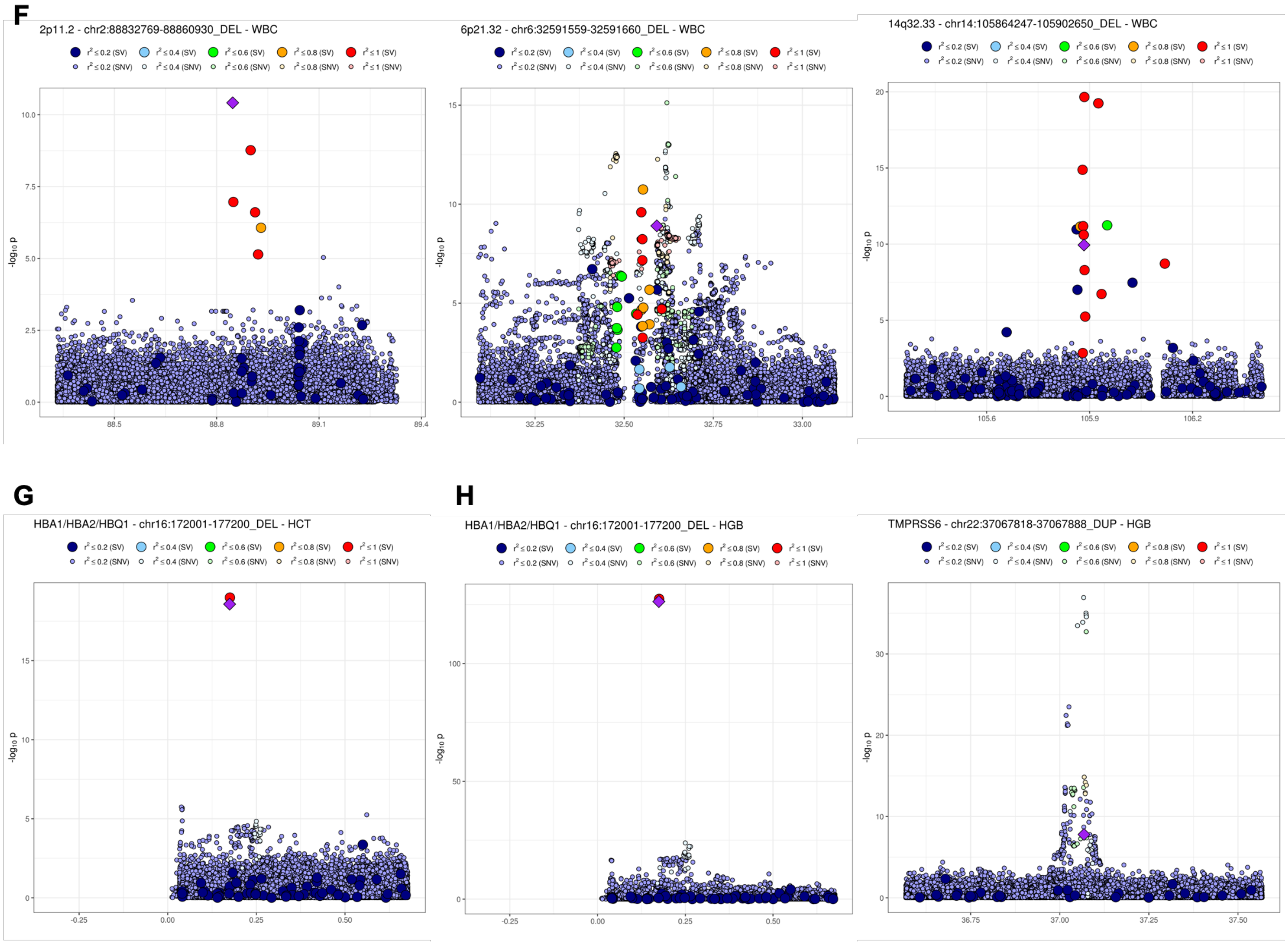
F
2p11.2 - chr2:88832769-88860930_DEL - WBC
6p21.32 - chr6:32591559-32591660_DEL - WBC
14q32.33 - chr14:105864247-105902650_DEL - WBC
G
HBA1/HBA2/HBQ1 - chr16:172001-177200_DEL - HCT
H
HBA1/HBA2/HBQ1 - chr16:172001-177200_DEL - HGB
TMPRSS6 - chr22:37067818-37067888_DUP - HGB
-log10 p

I

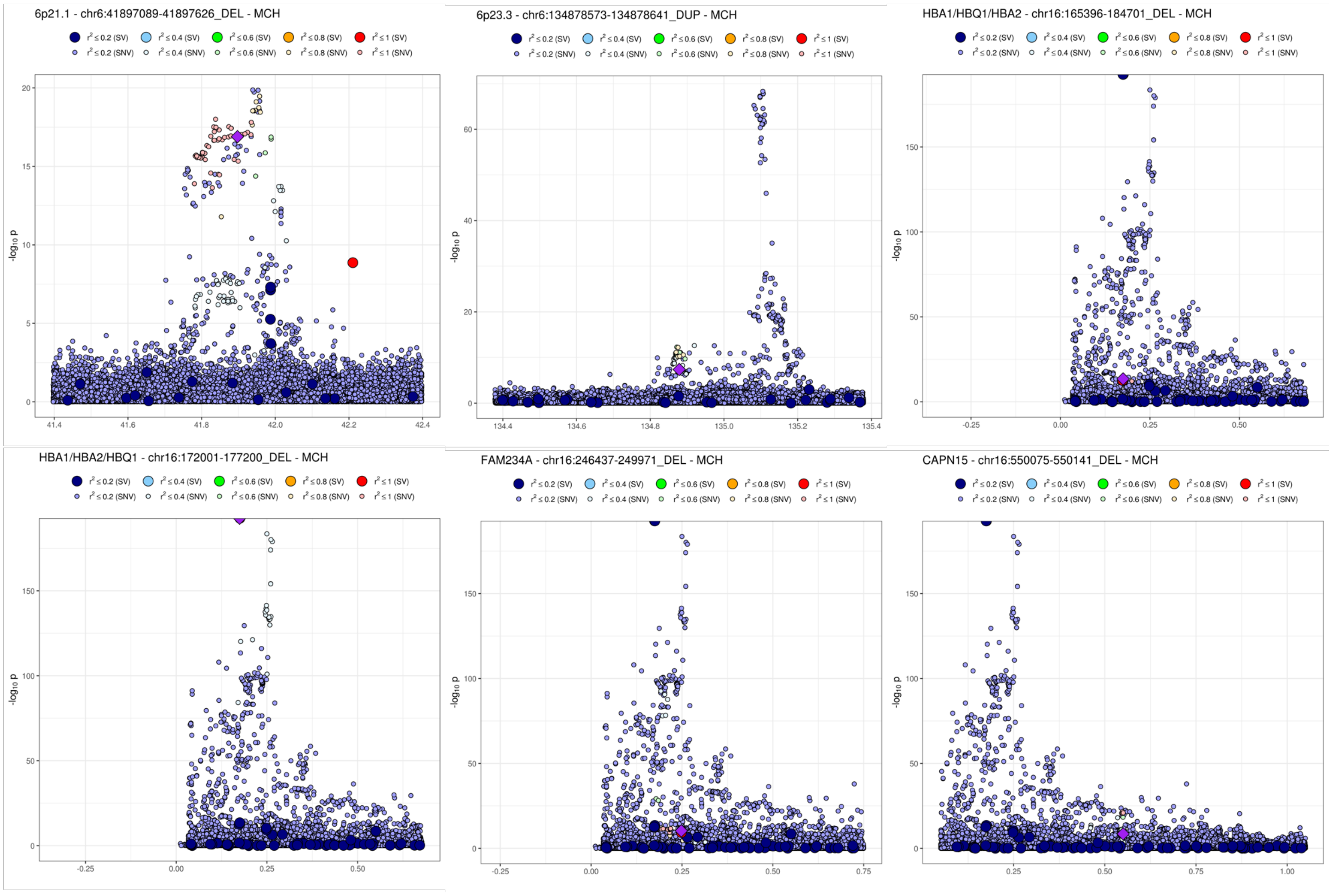

6p21.1 - chr6:41897089-41897626_DEL - MCH
6p23.3 - chr6:134878573-134878641_DUP - MCH
HBA1/HBQ1/HBA2 - chr16:165396-184701_DEL - MCH
HBA1/HBA2/HBQ1 - chr16:172001-177200_DEL - MCH
FAM234A - chr16:246437-249971_DEL - MCH
CAPN15 - chr16:550075-550141_DEL - MCH
r2 ≤ 0.2 (SV)
r2 ≤ 0.4 (SV)
r2 ≤ 0.6 (SV)
r2 ≤ 0.8 (SV)
r2 ≤ 1 (SV)
r2 ≤ 0.2 (SNV)
r2 ≤ 0.4 (SNV)
r2 ≤ 0.6 (SNV)
r2 ≤ 0.8 (SNV)
r2 ≤ 1 (SNV)
-log10 p

J

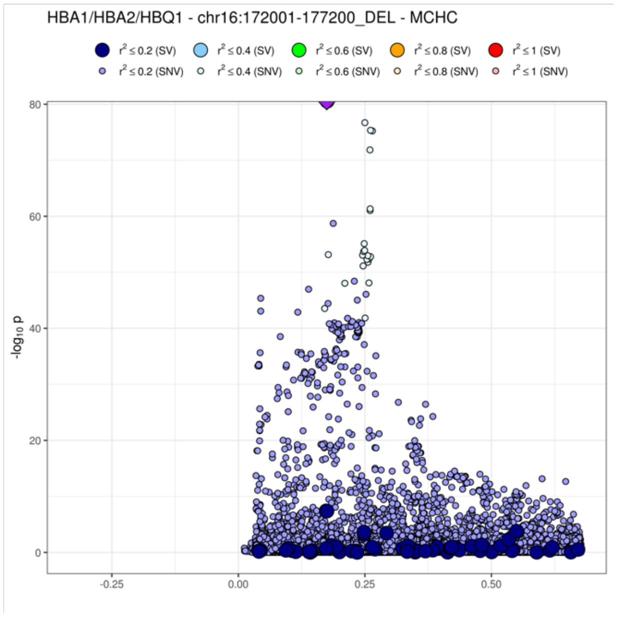

HBA1/HBA2/HBQ1 - chr16:172001-177200_DEL - MCHC
r2 ≤ 0.2 (SV)
r2 ≤ 0.4 (SV)
r2 ≤ 0.6 (SV)
r2 ≤ 0.8 (SV)
r2 ≤ 1 (SV)
r2 ≤ 0.2 (SNV)
r2 ≤ 0.4 (SNV)
r2 ≤ 0.6 (SNV)
r2 ≤ 0.8 (SNV)
r2 ≤ 1 (SNV)
-log10 p

K

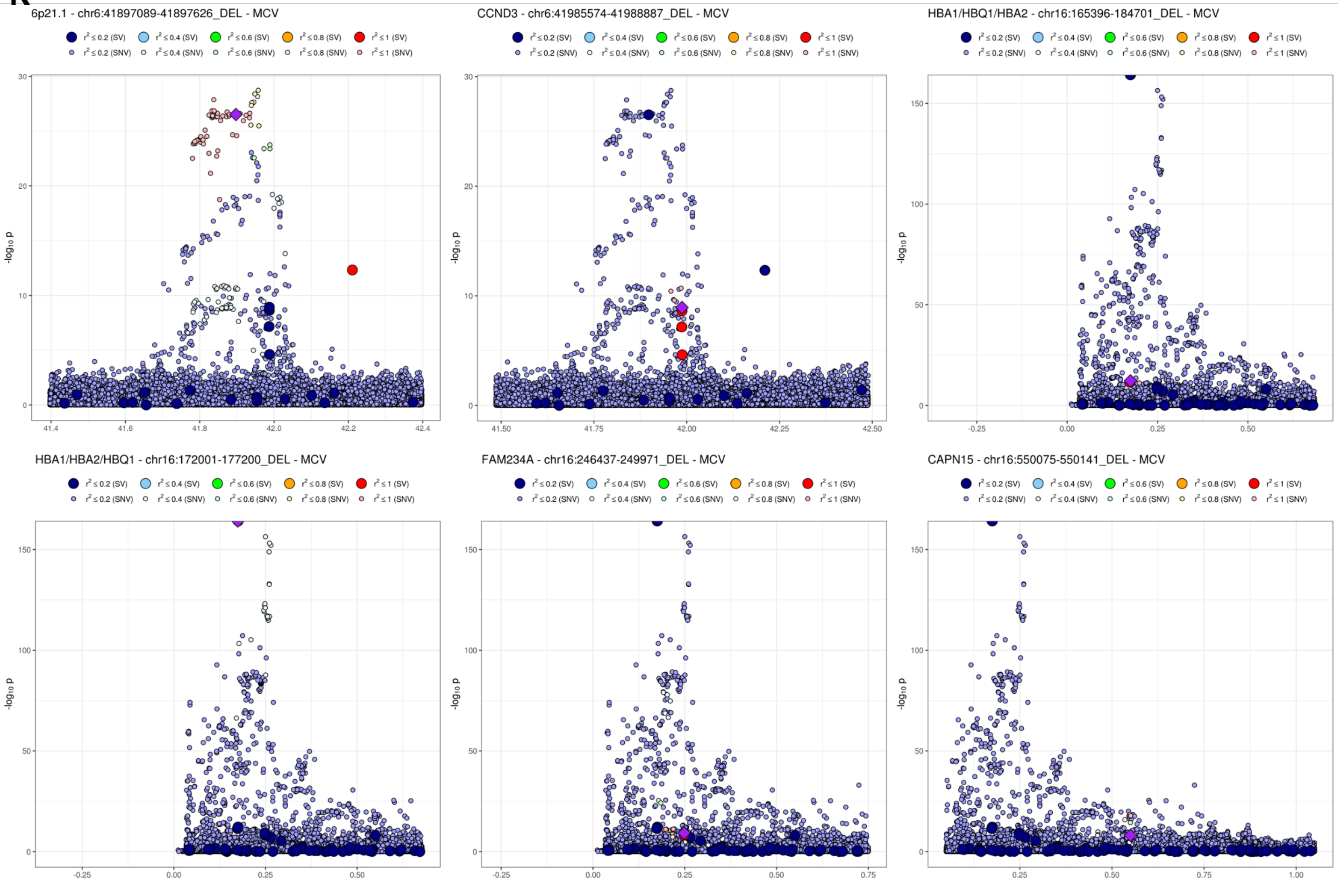
6p21.1 - chr6:41897089-41897626_DEL - MCV
CCND3 - chr6:41985574-41988887_DEL - MCV
HBA1/HBQ1/HBA2 - chr16:165396-184701_DEL - MCV
HBA1/HBA2/HBQ1 - chr16:172001-177200_DEL - MCV
FAM234A - chr16:246437-249971_DEL - MCV
CAPN15 - chr16:550075-550141_DEL - MCV
$r^2 \leq 0.2$ (SV)
$r^2 \leq 0.4$ (SV)
$r^2 \leq 0.6$ (SV)
$r^2 \leq 0.8$ (SV)
$r^2 \leq 1$ (SV)
$r^2 \leq 0.2$ (SNV)
$r^2 \leq 0.4$ (SNV)
$r^2 \leq 0.6$ (SNV)
$r^2 \leq 0.8$ (SNV)
$r^2 \leq 1$ (SNV)
$-\log_{10} p$

L

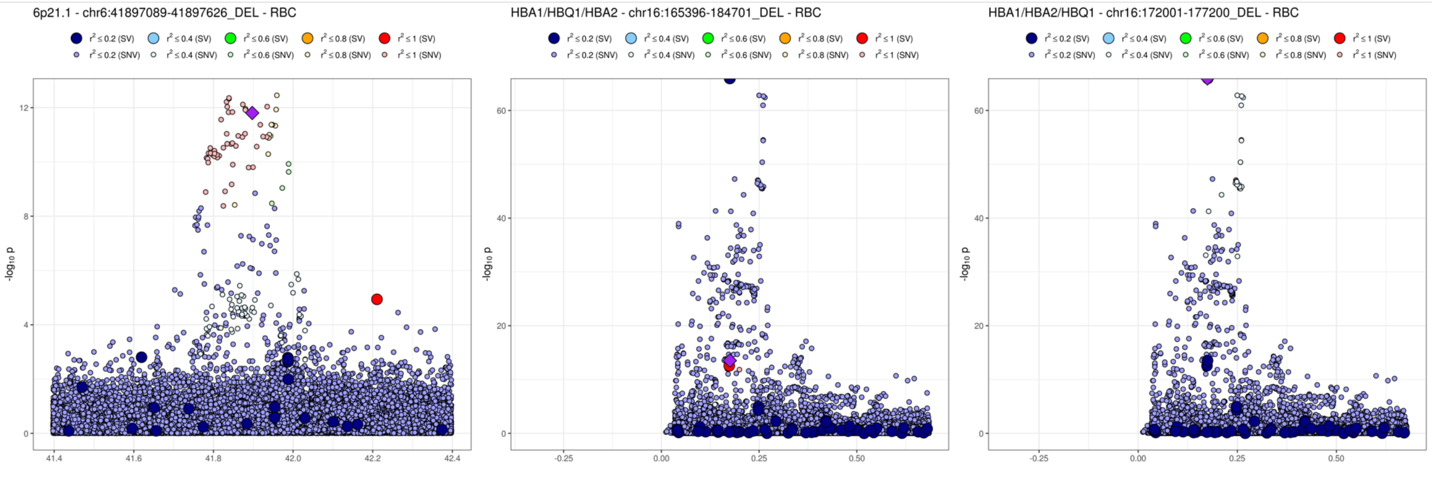
6p21.1 - chr6:41897089-41897626_DEL - RBC
HBA1/HBQ1/HBA2 - chr16:165396-184701_DEL - RBC
HBA1/HBA2/HBQ1 - chr16:172001-177200_DEL - RBC
$r^2 \leq 0.2$ (SV)
$r^2 \leq 0.4$ (SV)
$r^2 \leq 0.6$ (SV)
$r^2 \leq 0.8$ (SV)
$r^2 \leq 1$ (SV)
$r^2 \leq 0.2$ (SNV)
$r^2 \leq 0.4$ (SNV)
$r^2 \leq 0.6$ (SNV)
$r^2 \leq 0.8$ (SNV)
$r^2 \leq 1$ (SNV)
$-\log_{10} p$

M

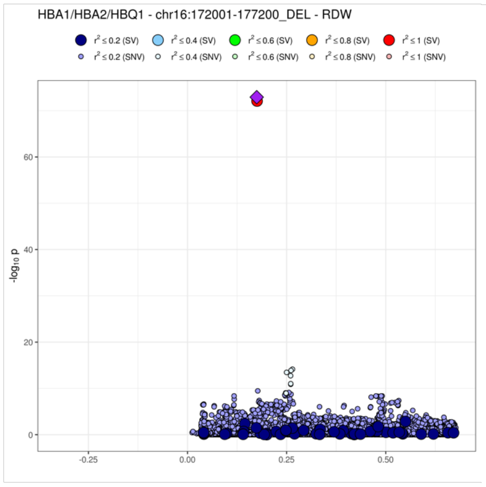

HBA1/HBA2/HBQ1 - chr16:172001-177200_DEL - RDW
r2 ≤ 0.2 (SV) r2 ≤ 0.4 (SV) r2 ≤ 0.6 (SV) r2 ≤ 0.8 (SV) r2 ≤ 1 (SV)
r2 ≤ 0.2 (SNV) r2 ≤ 0.4 (SNV) r2 ≤ 0.6 (SNV) r2 ≤ 0.8 (SNV) r2 ≤ 1 (SNV)
-log10 p

N

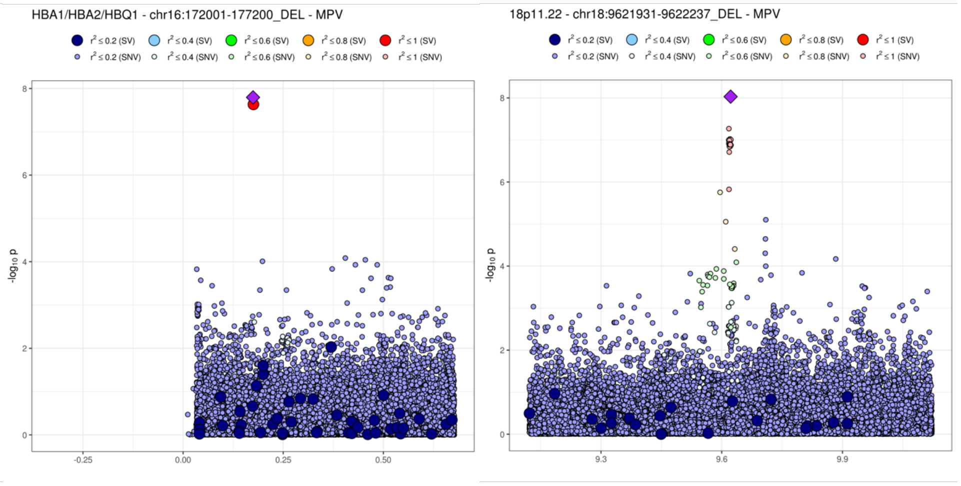

HBA1/HBA2/HBQ1 - chr16:172001-177200_DEL - MPV
18p11.22 - chr18:9621931-9622237_DEL - MPV
r2 ≤ 0.2 (SV) r2 ≤ 0.4 (SV) r2 ≤ 0.6 (SV) r2 ≤ 0.8 (SV) r2 ≤ 1 (SV)
r2 ≤ 0.2 (SNV) r2 ≤ 0.4 (SNV) r2 ≤ 0.6 (SNV) r2 ≤ 0.8 (SNV) r2 ≤ 1 (SNV)
-log10 p

O

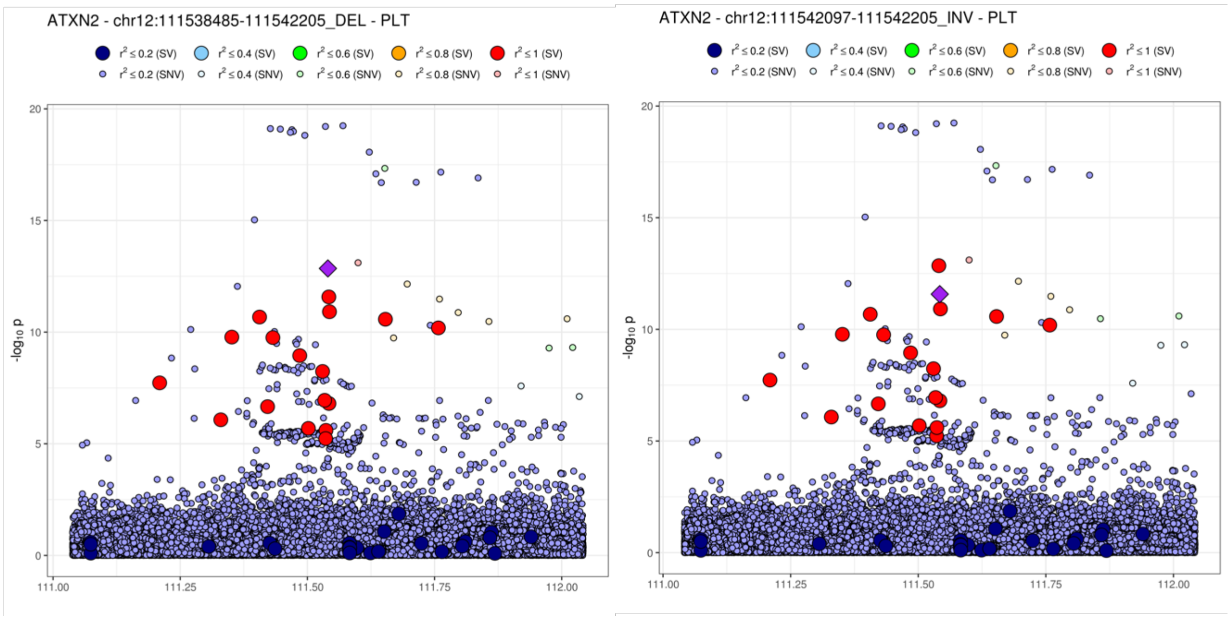

ATXN2 - chr12:111538485-111542205_DEL - PLT
ATXN2 - chr12:111542097-111542205_INV - PLT
r2 ≤ 0.2 (SV) r2 ≤ 0.4 (SV) r2 ≤ 0.6 (SV) r2 ≤ 0.8 (SV) r2 ≤ 1 (SV)
r2 ≤ 0.2 (SNV) r2 ≤ 0.4 (SNV) r2 ≤ 0.6 (SNV) r2 ≤ 0.8 (SNV) r2 ≤ 1 (SNV)
-log10 p

P

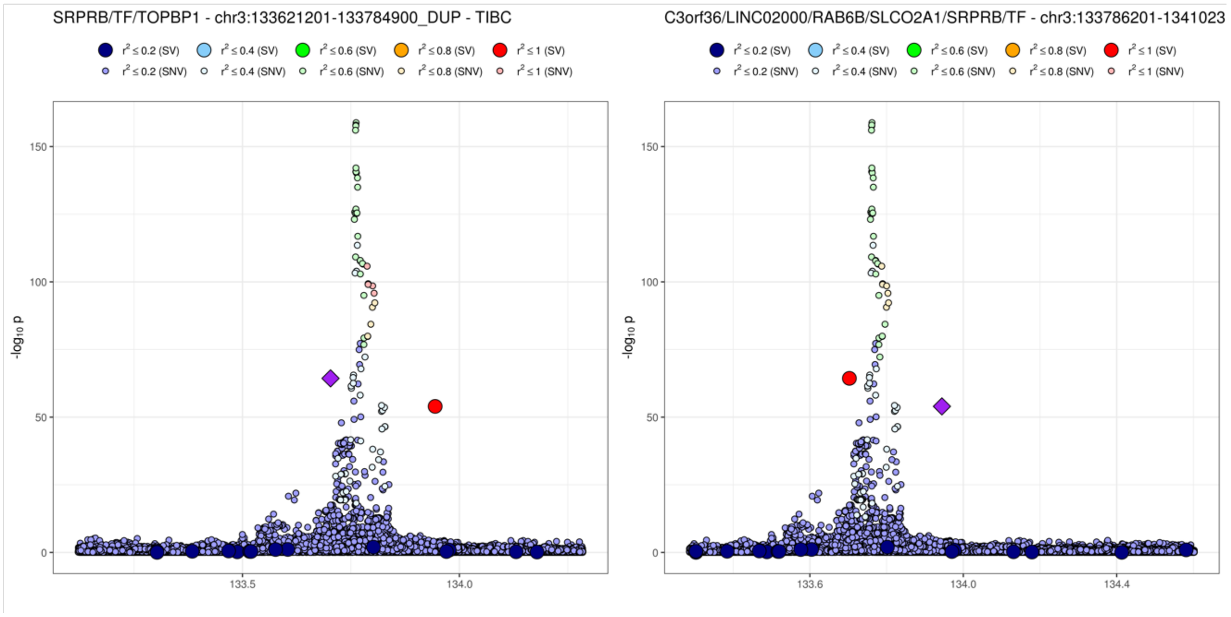
SRPRB/TF/TOPBP1 - chr3:133621201-133784900_DUP - TIBC
C3orf36/LINC02000/RAB6B/SLCO2A1/SRPRB/TF - chr3:133786201-1341023
r² ≤ 0.2 (SV) r² ≤ 0.4 (SV) r² ≤ 0.6 (SV) r² ≤ 0.8 (SV) r² ≤ 1 (SV)
r² ≤ 0.2 (SNV) r² ≤ 0.4 (SNV) r² ≤ 0.6 (SNV) r² ≤ 0.8 (SNV) r² ≤ 1 (SNV)
-log10 p
0
50
100
150
133.5
134.0
133.6
134.0
134.4

Q

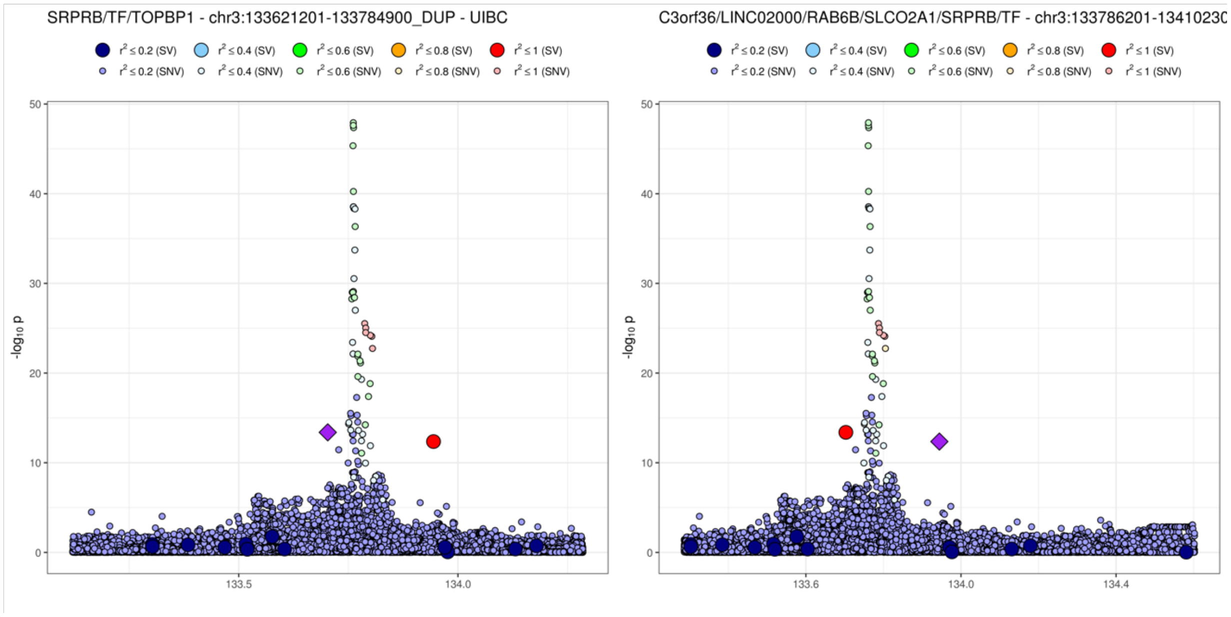
SRPRB/TF/TOPBP1 - chr3:133621201-133784900_DUP - UIBC
C3orf36/LINC02000/RAB6B/SLCO2A1/SRPRB/TF - chr3:133786201-1341023C
r² ≤ 0.2 (SV) r² ≤ 0.4 (SV) r² ≤ 0.6 (SV) r² ≤ 0.8 (SV) r² ≤ 1 (SV)
r² ≤ 0.2 (SNV) r² ≤ 0.4 (SNV) r² ≤ 0.6 (SNV) r² ≤ 0.8 (SNV) r² ≤ 1 (SNV)
-log10 p
0
10
20
30
40
50
133.5
134.0
133.6
134.0
134.4

Figure S5. Visualization of WGS reads in single representative samples for conditionally-independent trait associated SVs. Panels show WGS read visualization for a single representative sample predicted by Parliament 2 to exhibit an SV event in the A) 16p13.3 (alpha-globin) locus, B) 17p11.2 (KCNJ18) locus, C) 2p11.2 locus and D) 14q32.33 locus. Note WGS reads were visualized using SAMPLOT and display coverage and paired-end read evidence supporting SV events.

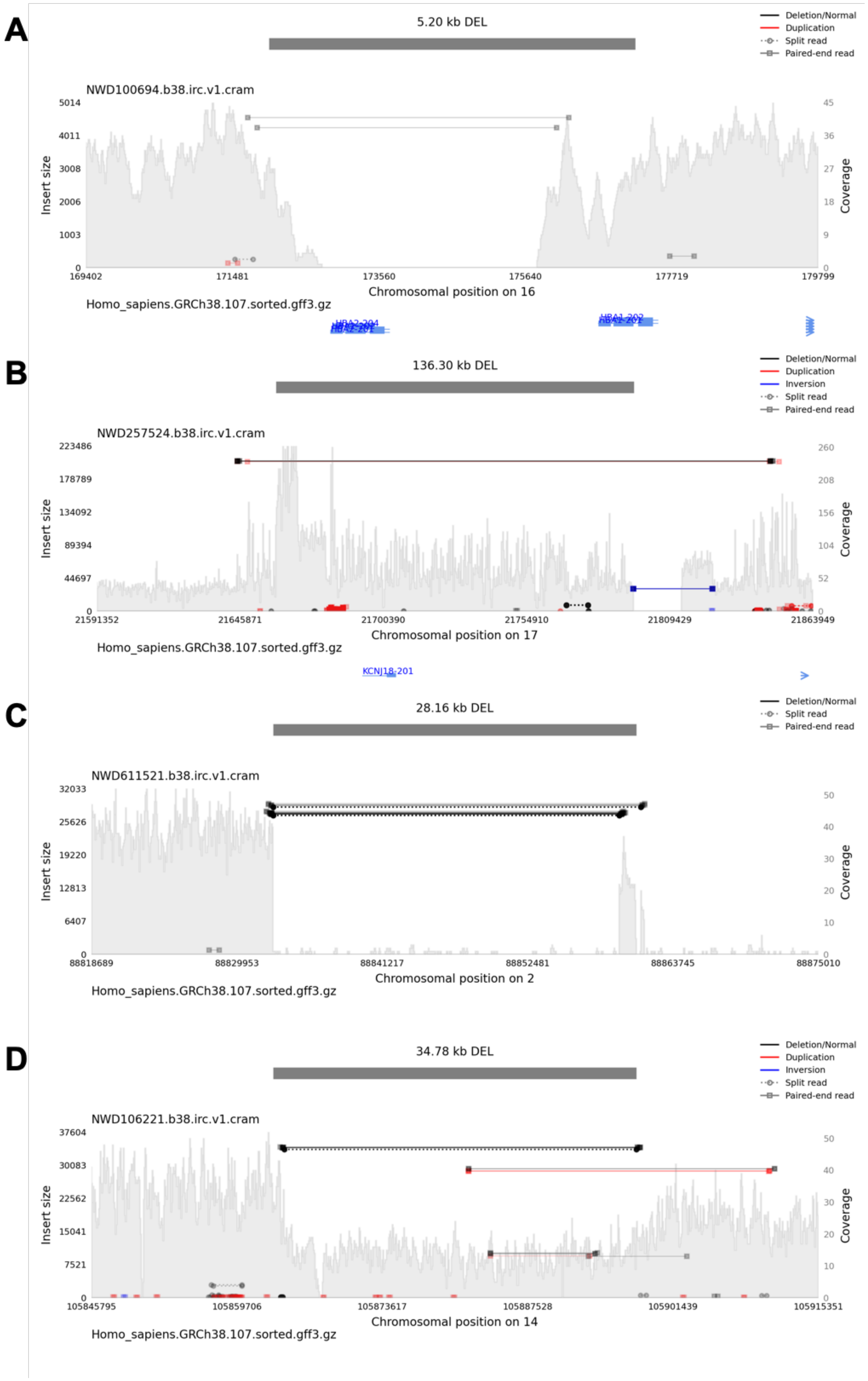
A
5.20 kb DEL
Deletion/Normal
Duplication
Split read
Paired-end read
NWD100694.b38.irc.v1.cram
Insert size
Coverage
5014
4011
3008
2006
1003
0
45
36
27
18
9
0
169402
171481
173560
175640
177719
179799
Chromosomal position on 16
Homo_sapiens.GRCh38.107.sorted.gff3.gz
HBA1-202
B
136.30 kb DEL
Deletion/Normal
Duplication
Inversion
Split read
Paired-end read
NWD257524.b38.irc.v1.cram
Insert size
Coverage
223486
178789
134092
89394
44697
0
260
208
156
104
52
0
21591352
21645871
21700390
21754910
21809429
21863949
Chromosomal position on 17
Homo_sapiens.GRCh38.107.sorted.gff3.gz
KCNJ18-201
C
28.16 kb DEL
Deletion/Normal
Split read
Paired-end read
NWD611521.b38.irc.v1.cram
Insert size
Coverage
32033
25626
19220
12813
6407
0
50
40
30
20
10
0
88818689
88829953
88841217
88852481
88863745
88875010
Chromosomal position on 2
Homo_sapiens.GRCh38.107.sorted.gff3.gz
D
34.78 kb DEL
Deletion/Normal
Duplication
Inversion
Split read
Paired-end read
NWD106221.b38.irc.v1.cram
Insert size
Coverage
37604
30083
22562
15041
7521
0
50
40
30
20
10
0
105845795
105859706
105873617
105887528
105901439
105915351
Chromosomal position on 14
Homo_sapiens.GRCh38.107.sorted.gff3.gz

Figure S6. Comparison of trait-association SV and SNV p-values in TOPMed for previously-reported SNVs from a European ancestry sample set[10] **.** The x-axis shows the p-values for the SNV in the TOPMed sample, and the y-axis shows the p-value for the SV that tags that SNV in the TOPMed sample (y axis). Only SNVs with an SV in LD at r^2 > 0.8 are shown**..** Points with p < 1e-4 in either analysis are labeled with the trait. All p-values are derived from two-sided t-tests and are not adjusted for multiple comparisons.

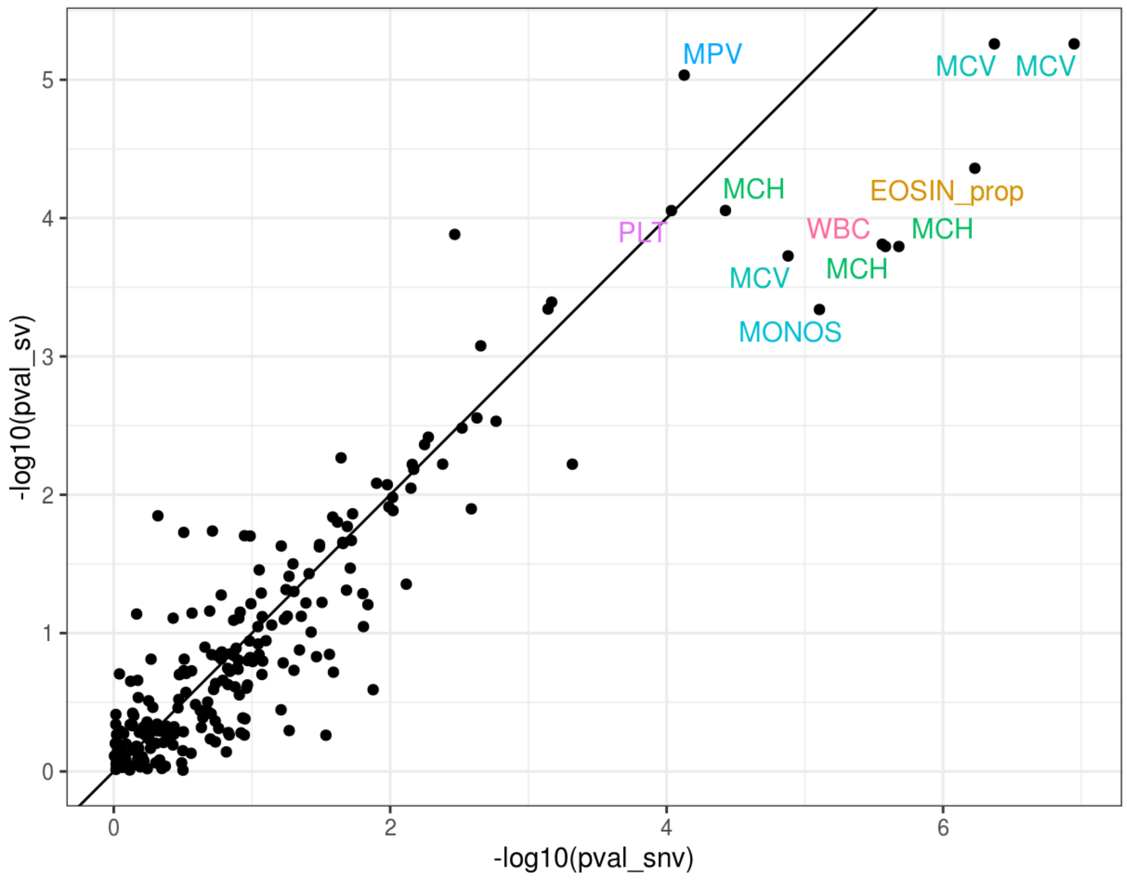

Figure S7. Chromatin status and chromatin conformation across the 9q22.1 locus (Related to Figure 1).

(A) Distribution of accessible chromatin (by DNase I sequencing) and histone modifications (H3K27ac, H3K4me1 and H3K4me3) in CD34+ common myeloid progenitors (CMP) and human mesenchymal stem cells (MSCs) across indicated genomic region. Datasets are obtained from the ENCODE project[27].

(B) Virtual 4C plot of long-range chromatin interactions in monocytes[28] with anchor positions shown with gray bars. Top panel shows deleted segment (chr9:88923551-88924152) as anchor position, bottom panel shows *S1PR3* promoter (chr9:91605763-91606263, bottoml) as anchor position. Reciprocally, yellow line highlights the *S1PR3* promoter (chr9:91605763-91606263, upper panel) and the deletion (chr9:88923551-88924152, lower panel). The observed and expected chromatin contact frequencies (or counts) are represented by the black and red lines, respectively. The left Y axis displays the range of chromatin contact frequency. The statistical significance (–log10(P-value)) of each long-range chromatin interaction is represented by the blue line, with its range listed in the right Y axis. All p-values are derived from two-sided t-tests and are not adjusted for multiple comparisons. The cell line or tissue specific FDR threshold (5%) is shown as a cyan horizontal dashed line, and the more stringent Bonferroni threshold (P = 0.05) is shown as a blue horizontal dashed line.

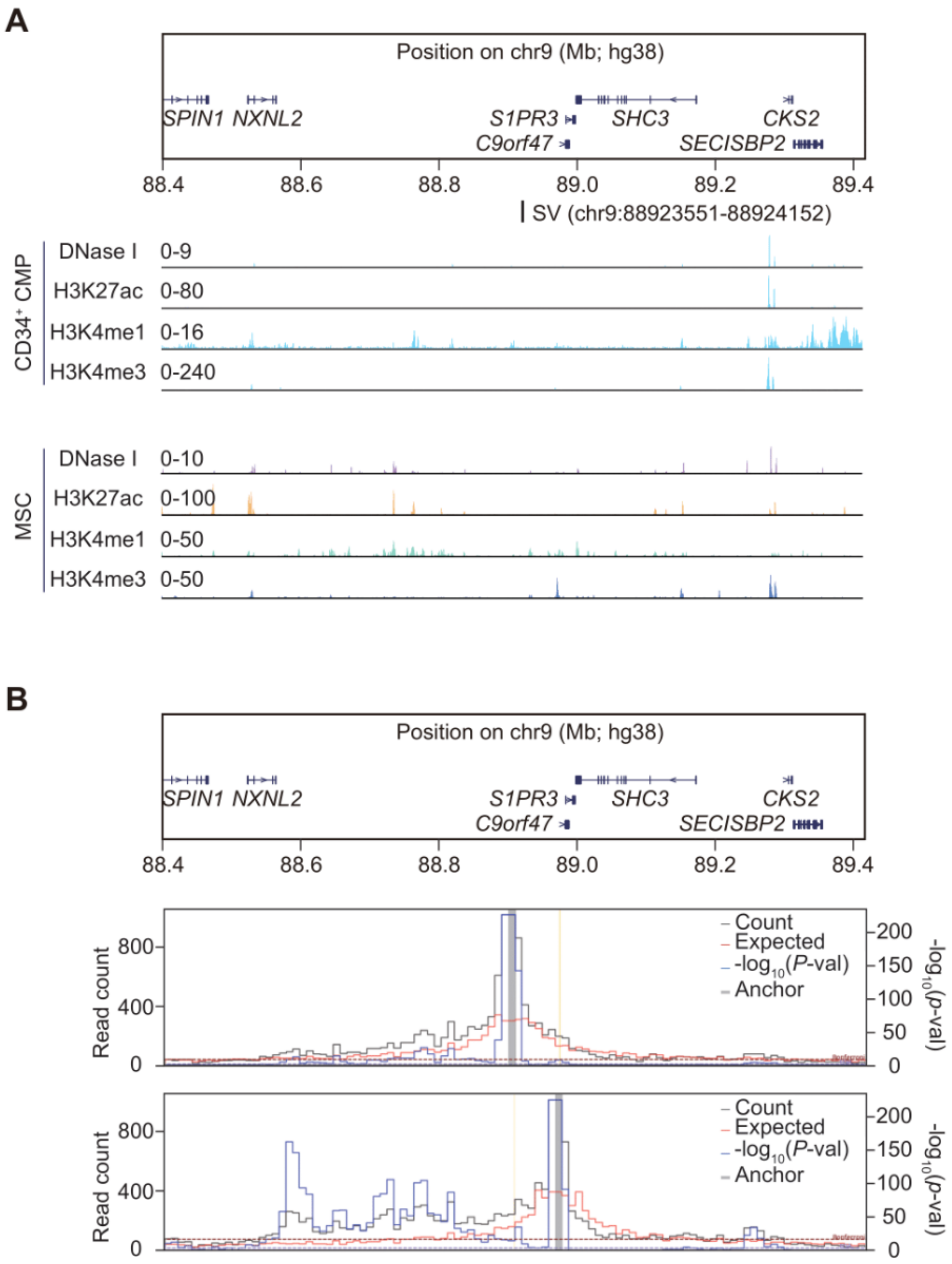

A
Position on chr9 (Mb; hg38)
SPIN1
NXNL2
S1PR3
SHC3
CKS2
C9orf47
SECISBP2
88.4
88.6
88.8
89.0
89.2
89.4
SV (chr9:88923551-88924152)
CD34+ CMP
DNase I 0-9
H3K27ac 0-80
H3K4me1 0-16
H3K4me3 0-240
MSC
DNase I 0-10
H3K27ac 0-100
H3K4me1 0-50
H3K4me3 0-50
B
Position on chr9 (Mb; hg38)
SPIN1
NXNL2
S1PR3
SHC3
CKS2
C9orf47
SECISBP2
88.4
88.6
88.8
89.0
89.2
89.4
Read count
Count
Expected
-log10(P-val)
Anchor
-log10(p-val)

Figure S8. Epigenome editing implicates *S1PR3* in the 9q22.1 monocyte count association (Related to Figure 2).

(A-B) eQTL analysis between the 9q22.1 SV and genes within 1 Mb window in peripheral blood mononuclear cells (PBMCs) (A) and T-cells (B) using data from the ancestry-stratified datasets from the Multi-Ethnic Study of Atherosclerosis (MESA, including n = 297 African American and n = 246 Hispanic/Latino participants for PBMC; n = 78 African American and n = 86 Hispanic/Latino participants for T-cell). AFHI: African American and Hispanic/Latino. All p-values are derived from two-sided t-tests and are not adjusted for multiple comparisons.

(C) Schematic of sgRNA locations targeting the *S1PR3* structural variant for CRISPRi with dCas9-KRAB (chr9:88923551-88924152I). PAM sequences highlighted in green.

(D-E) Expression of genes within a 1 Mb window in THP-1 cells expressing dCas9-KRAB after transduction with sgRNA #2 (D) or sgRNA #3 (E) targeting the *S1PR3* SV (orange) as compared to a neutral locus control sgRNA (blue). Relative mRNA level of each gene represented by mean ± standard deviation (SD). N = 3 replicates, where each replicate is a unique cellular transduction by sgRNA cassette. Two-sided Student's t-test. $P > 0.05$ for all comparisons except where indicated, * $P < 0.05$. Location of sgRNAs for CRISPRi are indicated (C). P-values for (D) and (E) were 0.043 and 0.033, respectively.

(F) Expression of each gene within a 1 Mb window around the 9q22.1 locus in THP-1 cells relative to *GAPDH*. Genes of undetectable expression level (*CDK20*, *SPATA31C2*, *SPIN1*, *SHC3*, *GADD45G* and *UNQ6494*) are not shown. N = 3 technical replicates. Relative mRNA level of each gene was represented by mean standard ± deviation (SD).

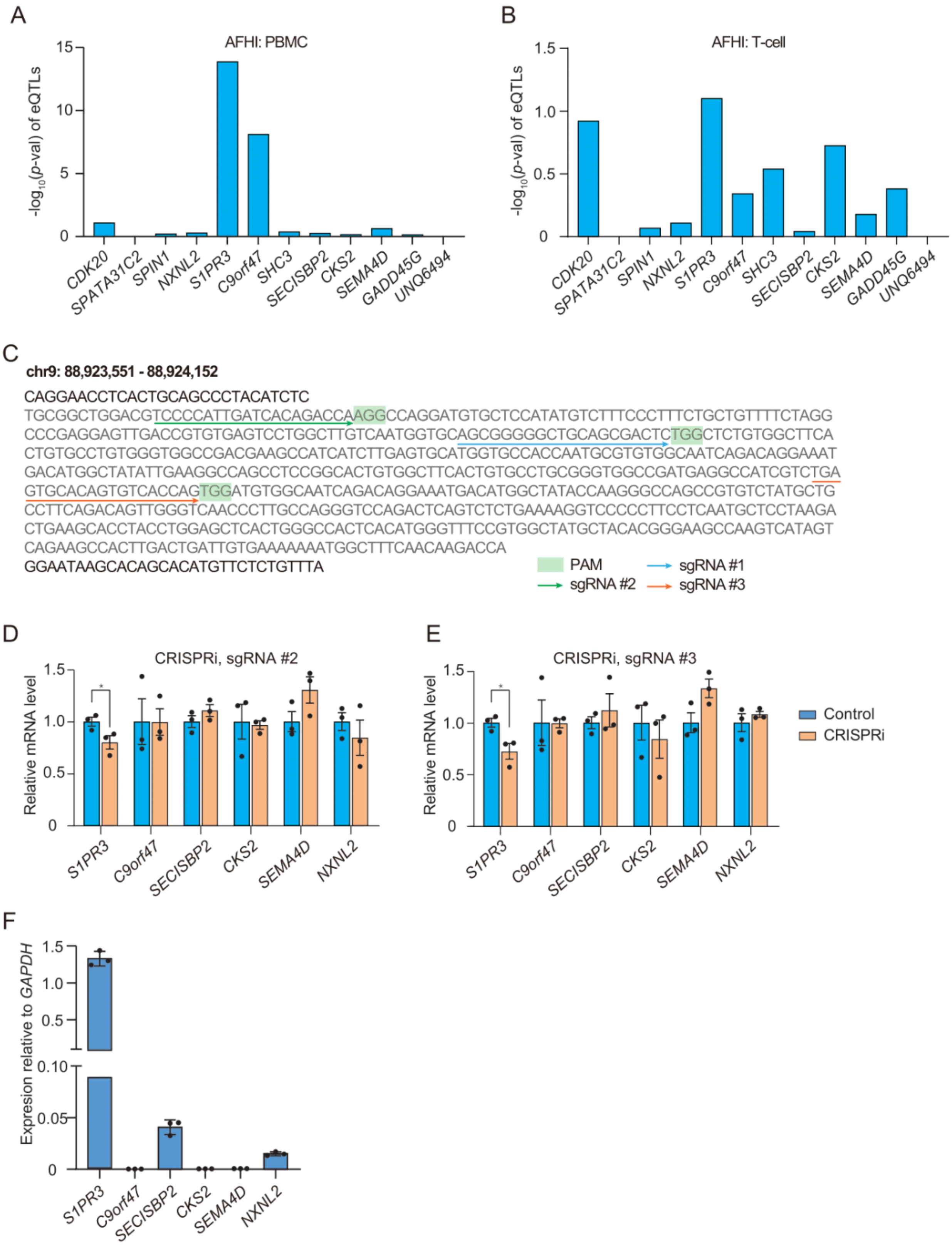
A
AFHI: PBMC
-log10(p-val) of eQTLs
15
10
5
0
CDK20
SPATA31C2
SPIN1
NXNL2
S1PR3
C9orf47
SHC3
SECISBP2
CKS2
SEMA4D
GADD45G
UNQ6494
B
AFHI: T-cell
-log10(p-val) of eQTLs
1.5
1.0
0.5
0
CDK20
SPATA31C2
SPIN1
NXNL2
S1PR3
C9orf47
SHC3
SECISBP2
CKS2
SEMA4D
GADD45G
UNQ6494
C
chr9: 88,923,551 - 88,924,152
CAGGAACCTCACTGCAGCCCTACATCTC
TGCGGCTGGACGTCCCCATTGATCACAGACCAAGGCCAGGATGTGCTCCATATGTCTTTCCCTTTCTGCTGTTTTCTAGG
CCCGAGGAGTTGACCGTGTGAGTCCTGGCTTGTCAATGGTGCAGCGGGGGCTGCAGCGACTCTGGCTCTGTGGCTTCA
CTGTGCCTGTGGGTGGCCGACGAAGCCATCATCTTGAGTGCATGGTGCCACCAATGCGTGTGGCAATCAGACAGGAAAT
GACATGGCTATATTGAAGGCCAGCCTCCGGCACTGTGGCTTCACTGTGCCTGCGGGTGGCCGATGAGGCCATCGTCTGA
GTGCACAGTGTCACCAGTGGATGTGGCAATCAGACAGGAAATGACATGGCTATACCAAGGGCCAGCCGTGTCTATGCTG
CCTTCAGACAGTTGGGTCAACCCTTGCCAGGGTCCAGACTCAGTCTCTGAAAAGGTCCCCCTTCCTCAATGCTCCTAAGA
CTGAAGCACCTACCTGGAGCTCACTGGGCCACTCACATGGGTTTCCGTGGCTATGCTACACGGGAAGCCAAGTCATAGT
CAGAAGCCACTTGACTGATTGTGAAAAAAATGGCTTTCAACAAGACCA
GGAATAAGCACAGCACATGTTCTCTGTTTA
PAM
sgRNA #1
sgRNA #2
sgRNA #3
D
CRISPRi, sgRNA #2
Relative mRNA level
1.5
1.0
0.5
0
*
S1PR3
C9orf47
SECISBP2
CKS2
SEMA4D
NXNL2
E
CRISPRi, sgRNA #3
Relative mRNA level
1.5
1.0
0.5
0
*
S1PR3
C9orf47
SECISBP2
CKS2
SEMA4D
NXNL2
Control
CRISPRi
F
Expresion relative to GAPDH
1.5
1.0
0.5
0.10
0.05
0
S1PR3
C9orf47
SECISBP2
CKS2
SEMA4D
NXNL2

Figure S9. Human hematopoietic reconstitution of immunodeficient mice after *S1PR3* editing (Related to Figure 2).

(A-D) Healthy donor CD34+ HSPCs were edited by indicated RNP and infused to NBSGW mice. After 12 weeks, bone marrow cells were analyzed by flow cytometry. Each symbol represents one mouse. HSPCs, hCD45+CD19-CD33-CD34+; B cells, hCD45+CD19+; Erythroid, hCD45-CD45-CD235a+; Myeloid, hCD45+CD33+. N=3 replicates, where each replicate indicates one mouse. Mean ± SD, 2-sided Mann-Whitney test, unadjusted for multiple comparisons.

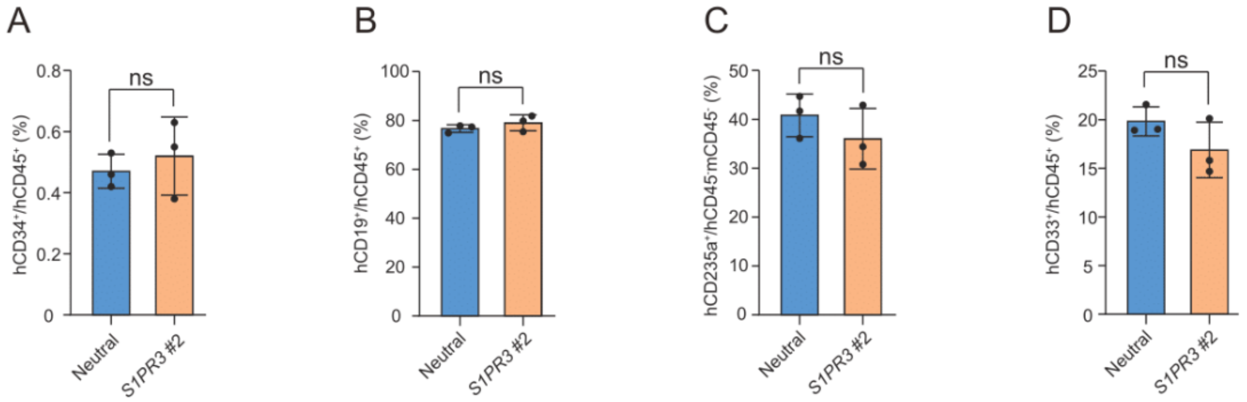

NHLBI TOPMed Consortium Author list

Name	Institution(s)	Primary Department	Institution City	Institution State	Zip Code	Country
Abe, Namiko	New York Genome Center		New York	New York	10013	US
Abecasis, Gonçalo	University of Michigan		Ann Arbor	Michigan	48109	US
Aguet, Francois	Broad Institute		Cambridge	Massachusetts	02142	US
Albert, Christine	Cedars Sinai		Boston	Massachusetts	02114	US
Almasy, Laura	Children's Hospital of Philadelphia, University of Pennsylvania		Philadelphia	Pennsylvania	19104	US
Alonso, Alvaro	Emory University		Atlanta	Georgia	30322	US
Ament, Seth	University of Maryland		Baltimore	Maryland	21201	US
Anderson, Peter	University of Washington		Seattle	Washington	98195	US
Anugu, Pramod	University of Mississippi		Jackson	Mississippi	38677	US
Applebaum-Bowden, Deborah	National Institutes of Health		Bethesda	Maryland	20892	US
Ardlie, Kristin	Broad Institute		Cambridge	Massachusetts	02142	US
Arking, Dan	Johns Hopkins University		Baltimore	Maryland	21218	US
Arnett, Donna K	University of South Carolina					US
Ashley-Koch, Allison	Duke University		Durham	North Carolina	27708	US
Aslibekyan, Stella	University of Alabama		Birmingham	Alabama	35487	US
Assimes, Tim	Stanford University		Stanford	California	94305	US
Auer, Paul	Medical College of Wisconsin		Milwaukee	Wisconsin	53211	US
Avramopoulos, Dimitrios	Johns Hopkins University		Baltimore	Maryland	21218	US

Name	Institution(s)	Primary Department	Institution City	Institution State	Zip Code	Country
Ayas, Najib	Providence Health Care	Medicine	Vancouver			CA
Balasubramanian, Adithya	Baylor College of Medicine Human Genome Sequencing Center		Houston	Texas	77030	US
Barnard, John	Cleveland Clinic		Cleveland	Ohio	44195	US
Barnes, Kathleen	Tempus, University of Colorado Anschutz Medical Campus		Aurora	Colorado	80045	US
Barr, R. Graham	Columbia University		New York	New York	10032	US
Barron-Casella, Emily	Johns Hopkins University		Baltimore	Maryland	21218	US
Barwick, Lucas	The Emmes Corporation	LTRC	Rockville	Maryland	20850	US
Beaty, Terri	Johns Hopkins University		Baltimore	Maryland	21218	US
Beck, Gerald	Cleveland Clinic	Quantitative Health Sciences	Cleveland	Ohio	44195	US
Becker, Diane	Johns Hopkins University	Medicine	Baltimore	Maryland	21218	US
Becker, Lewis	Johns Hopkins University		Baltimore	Maryland	21218	US
Beer, Rebecca	National Heart, Lung, and Blood Institute, National Institutes of Health		Bethesda	Maryland	20892	US
Beitelshees, Amber	University of Maryland		Baltimore	Maryland	21201	US
Benjamin, Emelia	Boston University, Massachusetts General Hospital	Boston University School of Medicine	Boston	Massachusetts	02114	US

Name	Institution(s)	Primary Department	Institution City	Institution State	Zip Code	Country
Benos, Takis	University of Pittsburgh		Pittsburgh	Pennsylvania	15260	US
Bezerra, Marcos	Fundação de Hematologia e Hemoterapia de Pernambuco - Hemope		Recife		52011-000	BR
Bielak, Larry	University of Michigan		Ann Arbor	Michigan	48109	US
Bis, Joshua	University of Washington	Cardiovascular Health Research Unit, Department of Medicine	Seattle	Washington	98195	US
Blackwell, Thomas	University of Michigan		Ann Arbor	Michigan	48109	US
Blangero, John	University of Texas Rio Grande Valley School of Medicine	Human Genetics	Brownsville	Texas	78520	US
Blue, Nathan	University of Utah	Obstetrics and Gynecology	Salt Lake City	Utah	84132	US
Boerwinkle, Eric	University of Texas Health at Houston		Houston	Texas	77225	US
Bowden, Donald W.	Wake Forest Baptist Health	Department of Biochemistry	Winston-Salem	North Carolina	27157	US
Bowler, Russell	National Jewish Health	National Jewish Health	Denver	Colorado	80206	US
Brody, Jennifer	University of Washington		Seattle	Washington	98195	US
Broeckel, Ulrich	Medical College of Wisconsin	Pediatrics	Milwaukee	Wisconsin	53226	US
Broome, Jai	University of Washington		Seattle	Washington	98195	US
Brown, Deborah	University of Texas Health at Houston	Pediatrics	Houston	Texas	77030	US
Bunting, Karen	New York Genome Center		New York	New York	10013	US

Name	Institution(s)	Primary Department	Institution City	Institution State	Zip Code	Country
Burchard, Esteban	University of California, San Francisco		San Francisco	California	94143	US
Bustamante, Carlos	Stanford University	Biomedical Data Science	Stanford	California	94305	US
Buth, Erin	University of Washington	Biostatistics	Seattle	Washington	98195	US
Cade, Brian	Brigham & Women's Hospital	Brigham and Women's Hospital	Boston	Massachusetts	02115	US
Cardwell, Jonathan	University of Colorado at Denver		Denver	Colorado	80204	US
Carey, Vincent	Brigham & Women's Hospital		Boston	Massachusetts	02115	US
Carrier, Julie	University of Montreal					US
Carson, April P.	University of Mississippi	Medicine	Jackson	Mississippi	39213	US
Carty, Cara	Washington State University		Pullman	Washington	99164	US
Casaburi, Richard	University of California, Los Angeles		Los Angeles	California	90095	US
Casas Romero, Juan P	Brigham & Women's Hospital					US
Casella, James	Johns Hopkins University		Baltimore	Maryland	21218	US
Castaldi, Peter	Brigham & Women's Hospital	Medicine	Boston	Massachusetts	02115	US
Chaffin, Mark	Broad Institute		Cambridge	Massachusetts	02142	US
Chang, Christy	University of Maryland		Baltimore	Maryland	21201	US
Chang, Yi-Cheng	National Taiwan University		Taipei		10617	TW
Chasman, Daniel	Brigham & Women's Hospital	Division of Preventive Medicine	Boston	Massachusetts	02215	US

Name	Institution(s)	Primary Department	Institution City	Institution State	Zip Code	Country
Chavan, Sameer	University of Colorado at Denver		Denver	Colorado	80204	US
Chen, Bo-Juen	New York Genome Center		New York	New York	10013	US
Chen, Wei-Min	University of Virginia		Charlottesville	Virginia	22903	US
Chen, Yii-Der Ida	Lundquist Institute		Torrance	California	90502	US
Cho, Michael	Brigham & Women's Hospital		Boston	Massachusetts	02115	US
Choi, Seung Hoan	Broad Institute		Cambridge	Massachusetts	02142	US
Chuang, Lee-Ming	National Taiwan University	National Taiwan University Hospital	Taipei		10617	TW
Chung, Mina	Cleveland Clinic	Cleveland Clinic	Cleveland	Ohio	44195	US
Chung, Ren-Hua	National Health Research Institute Taiwan		Miaoli County		350	TW
Clish, Clary	Broad Institute	Metabolomics Platform	Cambridge	Massachusetts	02142	US
Comhair, Suzy	Cleveland Clinic	Immunity and Immunology	Cleveland	Ohio	44195	US
Conomos, Matthew	University of Washington	Biostatistics	Seattle	Washington	98195	US
Cornell, Elaine	University of Vermont		Burlington	Vermont	05405	US
Correa, Adolfo	University of Mississippi	Population Health Science	Jackson	Mississippi	39216	US
Crandall, Carolyn	University of California, Los Angeles		Los Angeles	California	90095	US
Crapo, James	National Jewish Health		Denver	Colorado	80206	US
Cupples, L. Adrienne	Boston University	Biostatistics	Boston	Massachusetts	02115	US
Curran, Joanne	University of Texas Rio		Brownsville	Texas	78520	US

Name	Institution(s)	Primary Department	Institution City	Institution State	Zip Code	Country
	Grande Valley School of Medicine					
Curtis, Jeffrey	University of Michigan	Internal Medicine	Ann Arbor	Michigan	48109	US
Custer, Brian	Vitalant Research Institute		San Francisco	California	94118	US
Damcott, Coleen	University of Maryland		Baltimore	Maryland	21201	US
Darbar, Dawood	University of Illinois at Chicago		Chicago	Illinois	60607	US
David, Sean	University of Chicago		Chicago	Illinois	60637	US
Davis, Colleen	University of Washington		Seattle	Washington	98195	US
Daya, Michelle	University of Colorado at Denver		Denver	Colorado	80204	US
de Andrade, Mariza	Mayo Clinic	Health Quantitative Sciences Research	Rochester	Minnesota	55905	US
de las Fuentes, Lisa	Washington University in St Louis	Department of Medicine, Cardiovascular Division	St. Louis	Missouri	63110	US
de Vries, Paul	University of Texas Health at Houston	Human Genetics Center, Department of Epidemiology, Human Genetics, and Environmental Sciences	Houston	Texas	77030	US
DeBaun, Michael	Vanderbilt University		Nashville	Tennessee	37235	US
Deka, Ranjan	University of Cincinnati		Cincinnati	Ohio	45220	US
DeMeo, Dawn	Brigham & Women's Hospital		Boston	Massachusetts	02115	US
Devine, Scott	University of Maryland		Baltimore	Maryland	21201	US
Dinh, Huyen	Baylor College of Medicine		Houston	Texas	77030	US

Name	Institution(s)	Primary Department	Institution City	Institution State	Zip Code	Country
	Human Genome Sequencing Center					
Doddapaneni, Harsha	Baylor College of Medicine Human Genome Sequencing Center		Houston	Texas	77030	
Duan, Qing	University of North Carolina		Chapel Hill	North Carolina	27599	US
Dugan-Perez, Shannon	Baylor College of Medicine Human Genome Sequencing Center	BCM	Houston	Texas	77030	US
Duggirala, Ravi	University of Texas Rio Grande Valley School of Medicine		Edinburg	Texas	78539	US
Durda, Jon Peter	University of Vermont		Burlington	Vermont	05405	US
Dutcher, Susan K.	Washington University in St Louis	Genetics	St Louis	Missouri	63110	US
Eaton, Charles	Brown University		Providence	Rhode Island	02912	US
Ekunwe, Lynette	University of Mississippi		Jackson	Mississippi	38677	US
El Boueiz, Adel	Harvard University	Channing Division of Network Medicine	Cambridge	Massachusetts	02138	US
Ellinor, Patrick	Massachusetts General Hospital		Boston	Massachusetts	02114	US
Emery, Leslie	University of Washington		Seattle	Washington	98195	US
Erzurum, Serpil	Cleveland Clinic	Lerner Research Institute	Cleveland	Ohio	44195	US
Farber, Charles	University of Virginia		Charlottesville	Virginia	22903	US

Name	Institution(s)	Primary Department	Institution City	Institution State	Zip Code	Country
Farek, Jesse	Baylor College of Medicine Human Genome Sequencing Center		Houston	Texas	77030	US
Fingerlin, Tasha	National Jewish Health	Center for Genes, Environment and Health	Denver	Colorado	80206	US
Flickinger, Matthew	University of Michigan		Ann Arbor	Michigan	48109	US
Fornage, Myriam	University of Texas Health at Houston		Houston	Texas	77225	US
Franceschini, Nora	University of North Carolina	Epidemiology	Chapel Hill	North Carolina	27599	US
Frazar, Chris	University of Washington		Seattle	Washington	98195	US
Fu, Mao	University of Maryland		Baltimore	Maryland	21201	US
Fullerton, Stephanie M.	University of Washington		Seattle	Washington	98195	US
Fulton, Lucinda	Washington University in St Louis		St Louis	Missouri	63130	US
Gabriel, Stacey	Broad Institute		Cambridge	Massachusetts	02142	US
Gan, Weiniu	National Heart, Lung, and Blood Institute, National Institutes of Health		Bethesda	Maryland	20892	US
Gao, Shanshan	University of Colorado at Denver		Denver	Colorado	80204	US
Gao, Yan	University of Mississippi		Jackson	Mississippi	38677	US
Gass, Margery	Fred Hutchinson Cancer Research Center		Seattle	Washington	98109	US

Name	Institution(s)	Primary Department	Institution City	Institution State	Zip Code	Country
Geiger, Heather	New York Genome Center		New York City	New York	10013	US
Gelb, Bruce	Icahn School of Medicine at Mount Sinai		New York	New York	10029	US
Geraci, Mark	University of Pittsburgh		Pittsburgh	Pennsylvania		US
Germer, Soren	New York Genome Center		New York	New York	10013	US
Gerszten, Robert	Beth Israel Deaconess Medical Center		Boston	Massachusetts	02215	US
Ghosh, Auyon	Brigham & Women's Hospital		Boston	Massachusetts	02115	US
Gibbs, Richard	Baylor College of Medicine Human Genome Sequencing Center		Houston	Texas	77030	US
Gignoux, Chris	Stanford University		Stanford	California	94305	US
Gladwin, Mark	University of Pittsburgh		Pittsburgh	Pennsylvania	15260	US
Glahn, David	Boston Children's Hospital, Harvard Medical School	Department of Psychiatry	Boston	Massachusetts	02115	US
Gogarten, Stephanie	University of Washington		Seattle	Washington	98195	US
Gong, Da-Wei	University of Maryland		Baltimore	Maryland	21201	US
Goring, Harald	University of Texas Rio Grande Valley School of Medicine		San Antonio	Texas	78229	US
Graw, Sharon	University of Colorado Anschutz		Aurora	Colorado	80045	US

Name	Institution(s)	Primary Department	Institution City	Institution State	Zip Code	Country
	Medical Campus					
Gray, Kathryn J.	Mass General Brigham	Obstetrics and Gynecology	Boston	Massachusetts	02115	US
Grine, Daniel	University of Colorado at Denver		Denver	Colorado	80204	US
Gross, Colin	University of Michigan		Ann Arbor	Michigan	48109	US
Gu, C. Charles	Washington University in St Louis		St Louis	Missouri	63130	US
Guan, Yue	University of Maryland		Baltimore	Maryland	21201	US
Guo, Xiuqing	Lundquist Institute		Torrance	California	90502	US
Gupta, Namrata	Broad Institute		Cambridge	Massachusetts	02142	US
Haessler, Jeff	Fred Hutchinson Cancer Research Center		Seattle	Washington	98109	US
Hall, Michael	University of Mississippi	Cardiology	Jackson	Mississippi	39216	US
Han, Yi	Baylor College of Medicine Human Genome Sequencing Center		Houston	Texas	77030	US
Hanly, Patrick	University of Calgary	Medicine	Calgary			CA
Harris, Daniel	University of Maryland	Genetics	Philadelphia	Pennsylvania	19104	US
Hawley, Nicola L.	Yale University	Department of Chronic Disease Epidemiology	New Haven	Connecticut	06520	US
He, Jiang	Tulane University		New Orleans	Louisiana	70118	US
Heavner, Ben	University of Washington	Biostatistics	Seattle	Washington	98195	US
Heckbert, Susan	University of Washington	Epidemiology	Seattle	Washington	98195	US

Name	Institution(s)	Primary Department	Institution City	Institution State	Zip Code	Country
Hernandez, Ryan	University of California, San Francisco		San Francisco	California	94143	US
Herrington, David	Wake Forest Baptist Health		Winston-Salem	North Carolina	27157	US
Hersh, Craig	Brigham & Women's Hospital	Channing Division of Network Medicine	Boston	Massachusetts	02115	US
Hidalgo, Bertha	University of Alabama		Birmingham	Alabama	35487	US
Hixson, James	University of Texas Health at Houston		Houston	Texas	77225	US
Hobbs, Brian	Brigham & Women's Hospital		Boston	Massachusetts	02115	US
Hokanson, John	University of Colorado at Denver		Denver	Colorado	80204	US
Hong, Elliott	University of Maryland		Baltimore	Maryland	21201	US
Hoth, Karin	University of Iowa		Iowa City	Iowa	52242	US
Hsiung, Chao (Agnes)	National Health Research Institute Taiwan	Institute of Population Health Sciences, NHRI	Miaoli County		350	TW
Hu, Jianhong	Baylor College of Medicine Human Genome Sequencing Center		Houston	Texas	77030	US
Hung, Yi-Jen	Tri-Service General Hospital National Defense Medical Center					TW
Huston, Haley	Blood Works Northwest		Seattle	Washington	98104	US
Hwu, Chii Min	Taichung Veterans General		Taichung City		407	TW

Name	Institution(s)	Primary Department	Institution City	Institution State	Zip Code	Country
	Hospital Taiwan					
Irvin, Marguerite Ryan	University of Alabama		Birmingham	Alabama	35487	US
Jackson, Rebecca	Oklahoma State University Medical Center	Internal Medicine, DIvision of Endocrinology, Diabetes and Metabolism	Columbus	Ohio	43210	US
Jain, Deepti	University of Washington		Seattle	Washington	98195	US
Jaquish, Cashell	National Heart, Lung, and Blood Institute, National Institutes of Health	NHLBI	Bethesda	Maryland	20892	US
Johnsen, Jill	Blood Works Northwest	Medicine	Seattle	Washington	98109	US
Johnson, Andrew	National Heart, Lung, and Blood Institute, National Institutes of Health		Bethesda	Maryland	20892	US
Johnson, Craig	University of Washington		Seattle	Washington	98195	US
Johnston, Rich	Emory University		Atlanta	Georgia	30322	US
Jones, Kimberly	Johns Hopkins University		Baltimore	Maryland	21218	US
Kang, Hyun Min	University of Michigan	Biostatistics	Ann Arbor	Michigan	48109	US
Kaplan, Robert	Albert Einstein College of Medicine		New York	New York	10461	US
Kardia, Sharon	University of Michigan		Ann Arbor	Michigan	48109	US
Kelly, Shannon	University of California, San Francisco		San Francisco	California	94118	US

Name	Institution(s)	Primary Department	Institution City	Institution State	Zip Code	Country
Kenny, Eimear	Icahn School of Medicine at Mount Sinai		New York	New York	10029	US
Kessler, Michael	University of Maryland		Baltimore	Maryland	21201	US
Khan, Alyna	University of Washington		Seattle	Washington	98195	US
Khan, Ziad	Baylor College of Medicine Human Genome Sequencing Center		Houston	Texas	77030	US
Kim, Wonji	Harvard University		Cambridge	Massachusetts	02138	US
Kimoff, John	McGill University		Montréal		QC H3A 0G4	CA
Kinney, Greg	University of Colorado at Denver	Epidemiology	Aurora	Colorado	80045	US
Konkle, Barbara	Blood Works Northwest	Medicine	Seattle	Washington	98104	US
Kooperberg, Charles	Fred Hutchinson Cancer Research Center		Seattle	Washington	98109	US
Kramer, Holly	Loyola University	Public Health Sciences	Maywood	Illinois	60153	US
Lange, Christoph	Harvard School of Public Health	Biostats	Boston	Massachusetts	02115	US
Lange, Ethan	University of Colorado at Denver		Denver	Colorado	80204	US
Lange, Leslie	University of Colorado at Denver	Medicine	Aurora	Colorado	80048	US
Laurie, Cathy	University of Washington		Seattle	Washington	98195	US
Laurie, Cecelia	University of Washington		Seattle	Washington	98195	US

Name	Institution(s)	Primary Department	Institution City	Institution State	Zip Code	Country
LeBoff, Meryl	Brigham & Women's Hospital		Boston	Massachusetts	02115	US
Lee, Jiwon	Brigham & Women's Hospital		Boston	Massachusetts	02115	US
Lee, Sandra	Baylor College of Medicine Human Genome Sequencing Center		Houston	Texas	77030	US
Lee, Wen-Jane	Taichung Veterans General Hospital Taiwan		Taichung City		407	TW
LeFaive, Jonathon	University of Michigan		Ann Arbor	Michigan	48109	US
Levine, David	University of Washington		Seattle	Washington	98195	US
Levy, Dan	National Heart, Lung, and Blood Institute, National Institutes of Health		Bethesda	Maryland	20892	US
Lewis, Joshua	University of Maryland		Baltimore	Maryland	21201	US
Li, Xiaohui	Lundquist Institute		Torrance	California	90502	US
Li, Yun	University of North Carolina		Chapel Hill	North Carolina	27599	US
Lin, Henry	Lundquist Institute		Torrance	California	90502	US
Lin, Honghuang	Boston University	University of Massachusetts Chan Medical School	Worcester	Massachusetts	01655	US
Lin, Xihong	Harvard School of Public Health		Boston	Massachusetts	02115	US
Liu, Simin	Brown University	Epidemiology and Medicine	Providence	Rhode Island	02912	US

Name	Institution(s)	Primary Department	Institution City	Institution State	Zip Code	Country
Liu, Yongmei	Duke University	Cardiology	Durham	North Carolina	27708	US
Liu, Yu	Stanford University	Cardiovascular Institute	Stanford	California	94305	US
Loos, Ruth J.F.	Icahn School of Medicine at Mount Sinai	The Charles Bronfman Institute for Personalized Medicine	New York	New York	10029	US
Lubitz, Steven	Massachusetts General Hospital		Boston	Massachusetts	02114	US
Lunetta, Kathryn	Boston University		Boston	Massachusetts	02215	US
Luo, James	National Heart, Lung, and Blood Institute, National Institutes of Health		Bethesda	Maryland	20892	US
Magalang, Ulysses	The Ohio State University	Division of Pulmonary, Critical Care and Sleep Medicine	Columbus	Ohio	43210	US
Mahaney, Michael	University of Texas Rio Grande Valley School of Medicine		Brownsville	Texas	78520	US
Make, Barry	Johns Hopkins University		Baltimore	Maryland	21218	US
Manichaikul, Ani	University of Virginia		Charlottesville	Virginia	22903	US
Manning, Alisa	Broad Institute, Harvard University, Massachusetts General Hospital					
Manson, JoAnn	Brigham & Women's Hospital		Boston	Massachusetts	02115	US
Martin, Lisa	George Washington University	cardiology	Washington	District of Columbia	20037	US

Name	Institution(s)	Primary Department	Institution City	Institution State	Zip Code	Country
Marton, Melissa	New York Genome Center		New York City	New York	10013	US
Mathai, Susan	University of Colorado at Denver		Denver	Colorado	80204	US
Mathias, Rasika	Johns Hopkins University		Baltimore	Maryland	21218	US
May, Susanne	University of Washington	Biostatistics	Seattle	Washington	98195	US
McArdle, Patrick	University of Maryland		Baltimore	Maryland	21201	US
McDonald, Merry-Lynn	University of Alabama	University of Alabama at Birmingham	Birmingham	Alabama	35487	US
McFarland, Sean	Harvard University		Cambridge	Massachusetts	02138	US
McGarvey, Stephen	Brown University	Epidemiology	Providence	Rhode Island	02912	US
McGoldrick, Daniel	University of Washington	Genome Sciences	Seattle	Washington	98195	US
McHugh, Caitlin	University of Washington	Biostatistics	Seattle	Washington	98195	US
McNeil, Becky	RTI International					US
Mei, Hao	University of Mississippi		Jackson	Mississippi	38677	US
Meigs, James	Massachusetts General Hospital	Medicine	Boston	Massachusetts	02114	US
Menon, Vipin	Baylor College of Medicine Human Genome Sequencing Center		Houston	Texas	77030	US
Mestroni, Luisa	University of Colorado Anschutz Medical Campus		Aurora	Colorado	80045	US
Metcalf, Ginger	Baylor College of Medicine Human		Houston	Texas	77030	US

Name	Institution(s)	Primary Department	Institution City	Institution State	Zip Code	Country
	Genome Sequencing Center					
Meyers, Deborah A	University of Arizona		Tucson	Arizona	85721	US
Mignot, Emmanuel	Stanford University	Center For Sleep Sciences and Medicine	Palo Alto	California	94304	US
Mikulla, Julie	National Heart, Lung, and Blood Institute, National Institutes of Health		Bethesda	Maryland	20892	US
Min, Nancy	University of Mississippi		Jackson	Mississippi	38677	US
Minear, Mollie	National Institute of Child Health and Human Development, National Institutes of Health		Bethesda	Maryland	20892	US
Minster, Ryan L	University of Pittsburgh		Pittsburgh	Pennsylvania	15260	US
Mitchell, Braxton D.	University of Maryland		Baltimore	Maryland	21201	US
Moll, Matt	Brigham & Women's Hospital	Medicine	Boston	Massachusetts	02115	US
Momin, Zeineen	Baylor College of Medicine Human Genome Sequencing Center		Houston	Texas	77030	US
Montasser, May E.	University of Maryland		Baltimore	Maryland	21201	US
Montgomery, Courtney	Oklahoma Medical Research Foundation	Genes and Human Disease	Oklahoma City	Oklahoma	73104	US

Name	Institution(s)	Primary Department	Institution City	Institution State	Zip Code	Country
Muzny, Donna	Baylor College of Medicine Human Genome Sequencing Center		Houston	Texas	77030	US
Mychaleckyj, Josyf C	University of Virginia		Charlottesville	Virginia	22903	US
Nadkarni, Girish	Icahn School of Medicine at Mount Sinai		New York	New York	10029	US
Naik, Rakhi	Johns Hopkins University		Baltimore	Maryland	21218	US
Naseri, Take	Ministry of Health, Government of Samoa		Apia			WS
Natarajan, Pradeep	Broad Institute		Cambridge	Massachusetts	02142	US
Nekhai, Sergei	Howard University		Washington	District of Columbia	20059	US
Nelson, Sarah C.	University of Washington	Biostatistics	Seattle	Washington	98195	US
Neltner, Bonnie	University of Colorado at Denver		Denver	Colorado	80204	US
Nessner, Caitlin	Baylor College of Medicine Human Genome Sequencing Center		Houston	Texas	77030	US
Nickerson, Deborah	University of Washington	Department of Genome Sciences	Seattle	Washington	98195	US
Nkechinyere, Osuji	Baylor College of Medicine Human Genome Sequencing Center		Houston	Texas	77030	US
North, Kari	University of North Carolina		Chapel Hill	North Carolina	27599	US
O'Connell, Jeff	University of Maryland		Balitmore	Maryland	21201	US

Name	Institution(s)	Primary Department	Institution City	Institution State	Zip Code	Country
O'Connor, Tim	University of Maryland		Baltimore	Maryland	21201	US
Ochs-Balcom, Heather	University at Buffalo		Buffalo	New York	14260	US
Okwuonu, Geoffrey	Baylor College of Medicine Human Genome Sequencing Center		Houston	Texas	77030	US
Pack, Allan	University of Pennsylvania	Division of Sleep Medicine/Department of Medicine	Philadelphia	Pennsylvania	19104-3403	US
Paik, David T.	Stanford University	Stanford Cardiovascular Institute	Stanford	California	94305	US
Palmer, Nicholette	Wake Forest Baptist Health	Biochemistry	Winston-Salem	North Carolina	27157	US
Pankow, James	University of Minnesota		Minneapolis	Minnesota	55455	US
Papanicolaou, George	National Heart, Lung, and Blood Institute, National Institutes of Health		Bethesda	Maryland	20892	US
Parker, Cora	RTI International	Biostatistics and Epidemiology Division	Research Triangle Park	North Carolina	27709-2194	US
Peloso, Gina	Boston University	Department of Biostatistics	Boston	Massachusetts	02118	US
Peralta, Juan Manuel	University of Texas Rio Grande Valley School of Medicine		Edinburg	Texas	78539	US
Perez, Marco	Stanford University		Stanford	California	94305	US
Perry, James	University of Maryland		Baltimore	Maryland	21201	US
Peters, Ulrike	Fred Hutchinson Cancer Research Center	Fred Hutch and UW	Seattle	Washington	98109	US

Name	Institution(s)	Primary Department	Institution City	Institution State	Zip Code	Country
Peyser, Patricia	University of Michigan		Ann Arbor	Michigan	48109	US
Phillips, Lawrence S	Emory University		Atlanta	Georgia	30322	US
Pleiness, Jacob	University of Michigan		Ann Arbor	Michigan	48109	US
Pollin, Toni	University of Maryland		Baltimore	Maryland	21201	US
Post, Wendy	Johns Hopkins University	Cardiology/Medicine	Baltimore	Maryland	21218	US
Powers Becker, Julia	University of Colorado at Denver	Medicine	Denver	Colorado	80204	US
Preethi Boorgula, Meher	University of Colorado at Denver		Denver	Colorado	80204	US
Preuss, Michael	Icahn School of Medicine at Mount Sinai		New York	New York	10029	US
Psaty, Bruce	University of Washington		Seattle	Washington	98195	US
Qasba, Pankaj	National Heart, Lung, and Blood Institute, National Institutes of Health		Bethesda	Maryland	20892	US
Qiao, Dandi	Brigham & Women's Hospital		Boston	Massachusetts	02115	US
Qin, Zhaohui	Emory University		Atlanta	Georgia	30322	US
Rafaels, Nicholas	University of Colorado at Denver	CCPM	Denver	Colorado	80045	US
Raffield, Laura	University of North Carolina	Genetics	Chapel Hill	North Carolina	27599	US
Rajendran, Mahitha	Baylor College of Medicine Human Genome Sequencing Center		Houston	Texas	77030	US

Name	Institution(s)	Primary Department	Institution City	Institution State	Zip Code	Country
Ramachandran, Vasan S.	Boston University		Boston	Massachusetts	02215	US
Rao, D.C.	Washington University in St Louis		St Louis	Missouri	63130	US
Rasmussen-Torvik, Laura	Northwestern University		Chicago	Illinois	60208	US
Ratan, Aakrosh	University of Virginia		Charlottesville	Virginia	22903	US
Redline, Susan	Brigham & Women's Hospital	Medicine	Boston	Massachusetts	02115	US
Reed, Robert	University of Maryland		Baltimore	Maryland	21201	US
Reeves, Catherine	New York Genome Center	New York Genome Center	New York City	New York	10013	US
Regan, Elizabeth	National Jewish Health		Denver	Colorado	80206	US
Reiner, Alex	Fred Hutchinson Cancer Research Center, University of Washington		Seattle	Washington	98109	US
Reupena, Muagututi‘a Sefuiva	Lutia I Puava Ae Mapu I Fagalele		Apia			WS
Rice, Ken	University of Washington		Seattle	Washington	98195	US
Rich, Stephen	University of Virginia		Charlottesville	Virginia	22903	US
Robillard, Rebecca	University of Ottawa	Sleep Research Unit, University of Ottawa Institute for Mental Health Research	Ottawa		ON K1Z 7K4	CA
Robine, Nicolas	New York Genome Center		New York City	New York	10013	US
Roden, Dan	Vanderbilt University	Medicine, Pharmacology, Biomedicla Informatics	Nashville	Tennessee	37235	US

Name	Institution(s)	Primary Department	Institution City	Institution State	Zip Code	Country
Roselli, Carolina	Broad Institute		Cambridge	Massachusetts	02142	US
Rotter, Jerome	Lundquist Institute	Pediatrics	Torrance	California	90502	US
Ruczinski, Ingo	Johns Hopkins University		Baltimore	Maryland	21218	US
Runnels, Alexi	New York Genome Center		New York City	New York	10013	US
Russell, Pamela	University of Colorado at Denver		Denver	Colorado	80204	US
Ruuska, Sarah	Blood Works Northwest		Seattle	Washington	98104	US
Ryan, Kathleen	University of Maryland		Baltimore	Maryland	21201	US
Sabino, Ester Cerdeira	Universidade de Sao Paulo	Faculdade de Medicina	Sao Paulo		01310000	BR
Saleheen, Danish	Columbia University		New York	New York	10027	US
Salimi, Shabnam	University of Maryland	Pathology	Seattle	Washington	98195	US
Salvi, Sejal	Baylor College of Medicine Human Genome Sequencing Center		Houston	Texas	77030	US
Salzberg, Steven	Johns Hopkins University		Baltimore	Maryland	21218	US
Sandow, Kevin	Lundquist Institute	TGPS	Torrance	California	90502	US
Sankaran, Vijay G.	Harvard University	Division of Hematology/Oncology	Boston	Massachusetts	02115	US
Santibanez, Jireh	Baylor College of Medicine Human Genome Sequencing Center		Houston	Texas	77030	US
Schwander, Karen	Washington University in St Louis		St Louis	Missouri	63130	US

Name	Institution(s)	Primary Department	Institution City	Institution State	Zip Code	Country
Schwartz, David	University of Colorado at Denver		Denver	Colorado	80204	US
Sciurba, Frank	University of Pittsburgh		Pittsburgh	Pennsylvania	15260	US
Seidman, Christine	Harvard Medical School	Genetics	Boston	Massachusetts	02115	US
Seidman, Jonathan	Harvard Medical School		Boston	Massachusetts	02115	US
Sériès, Frédéric	Université Laval		Quebec City		G1V 0A6	CA
Sheehan, Vivien	Emory University	Pediatrics	Atlanta	Georgia	30307	US
Sherman, Stephanie L.	Emory University	Human Genetics	Atlanta	Georgia	30322	US
Shetty, Amol	University of Maryland		Baltimore	Maryland	21201	US
Shetty, Aniket	University of Colorado at Denver		Denver	Colorado	80204	US
Sheu, Wayne Hui-Heng	Taichung Veterans General Hospital Taiwan		Taichung City		407	TW
Shoemaker, M. Benjamin	Vanderbilt University	Medicine/Cardiology	Nashville	Tennessee	37235	US
Silver, Brian	UMass Memorial Medical Center		Worcester	Massachusetts	01655	US
Silverman, Edwin	Brigham & Women's Hospital		Boston	Massachusetts	02115	US
Skomro, Robert	University of Saskatchewan		Saskatoon		SK S7N 5C9	CA
Smith, Albert Vernon	University of Michigan					
Smith, Jennifer	University of Michigan		Ann Arbor	Michigan	48109	US
Smith, Josh	University of Washington		Seattle	Washington	98195	US
Smith, Nicholas	University of Washington	Epidemiology	Seattle	Washington	98195	US

Name	Institution(s)	Primary Department	Institution City	Institution State	Zip Code	Country
Smith, Tanja	New York Genome Center		New York	New York	10013	US
Smoller, Sylvia	Albert Einstein College of Medicine		New York	New York	10461	US
Snively, Beverly	Wake Forest Baptist Health	Biostatistical Sciences	Winston-Salem	North Carolina	27157	US
Snyder, Michael	Stanford University		Stanford	California	94305	US
Sofer, Tamar	Brigham & Women's Hospital		Boston	Massachusetts	02115	US
Sotoodehnia, Nona	University of Washington		Seattle	Washington	98195	US
Stilp, Adrienne M.	University of Washington		Seattle	Washington	98195	US
Storm, Garrett	University of Colorado at Denver	Genomic Cardiology	Aurora	Colorado	80045	US
Streeten, Elizabeth	University of Maryland		Baltimore	Maryland	21201	US
Su, Jessica Lasky	Brigham & Women's Hospital	Channing Department of Medicine	Boston	Massachusetts	02115	US
Sung, Yun Ju	Washington University in St Louis		St Louis	Missouri	63130	US
Sylvia, Jody	Brigham & Women's Hospital		Boston	Massachusetts	02115	US
Szpiro, Adam	University of Washington		Seattle	Washington	98195	US
Taliun, Daniel	University of Michigan		Ann Arbor	Michigan	48109	US
Tang, Hua	Stanford University	Genetics	Stanford	California	94305	US
Taub, Margaret	Johns Hopkins University		Baltimore	Maryland	21218	US
Taylor, Kent D.	Lundquist Institute	Institute for Translational Genomics and Populations Sciences	Torrance	California	90502	US

Name	Institution(s)	Primary Department	Institution City	Institution State	Zip Code	Country
Taylor, Matthew	University of Colorado Anschutz Medical Campus		Aurora	Colorado	80045	US
Taylor, Simeon	University of Maryland		Baltimore	Maryland	21201	US
Telen, Marilyn	Duke University		Durham	North Carolina	27708	US
Thornton, Timothy A.	University of Washington		Seattle	Washington	98195	US
Threlkeld, Machiko	University of Washington	University of Washington, Department of Genome Sciences	Seattle	Washington	98195	US
Tinker, Lesley	Fred Hutchinson Cancer Research Center	Cancer Prevention Division of Public Health Sciences	Seattle	Washington	98109	US
Tirschwell, David	University of Washington		Seattle	Washington	98195	US
Tishkoff, Sarah	University of Pennsylvania	Genetics	Philadelphia	Pennsylvania	19104	US
Tiwari, Hemant	University of Alabama	Biostatistics	Birmingham	Alabama	35487	US
Tong, Catherine	University of Washington	Department of Biostatistics	Seattle	Washington	98195	US
Tracy, Russell	University of Vermont	Pathology & Laboratory Medicine	Burlington	Vermont	05405	US
Tsai, Michael	University of Minnesota		Minneapolis	Minnesota	55455	US
Vaidya, Dhananjay	Johns Hopkins University		Baltimore	Maryland	21218	US
Van Den Berg, David	University of Southern California	USC Methylation Characterization Center	University of Southern California	California	90033	US
VandeHaar, Peter	University of Michigan		Ann Arbor	Michigan	48109	US
Vrieze, Scott	University of Minnesota		Minneapolis	Minnesota	55455	US

Name	Institution(s)	Primary Department	Institution City	Institution State	Zip Code	Country
Walker, Tarik	University of Colorado at Denver		Denver	Colorado	80204	US
Wallace, Robert	University of Iowa		Iowa City	Iowa	52242	US
Walts, Avram	University of Colorado at Denver		Denver	Colorado	80204	US
Wang, Fei Fei	University of Washington		Seattle	Washington	98195	US
Wang, Heming	Brigham & Women's Hospital, Mass General Brigham		Boston	Massachusetts	02115	US
Wang, Jiongming	University of Michigan					US
Watson, Karol	University of California, Los Angeles		Los Angeles	California	90095	US
Watt, Jennifer	Baylor College of Medicine Human Genome Sequencing Center		Houston	Texas	77030	US
Weeks, Daniel E.	University of Pittsburgh	Department of Human Genetics	Pittsburgh	Pennsylvania	15260	US
Weinstock, Joshua	University of Michigan	Biostatistics	Ann Arbor	Michigan	48109	US
Weir, Bruce	University of Washington		Seattle	Washington	98195	US
Weiss, Scott T	Brigham & Women's Hospital	Channing Division of Network Medicine, Department of Medicine	Boston	Massachusetts	02115	US
Weng, Lu-Chen	Massachusetts General Hospital		Boston	Massachusetts	02114	US
Wessel, Jennifer	Indiana University	Epidemiology	Indianapolis	Indiana	46202	US
Willer, Cristen	University of Michigan	Internal Medicine	Ann Arbor	Michigan	48109	US

Name	Institution(s)	Primary Department	Institution City	Institution State	Zip Code	Country
Williams, Kayleen	University of Washington	Biostatistics	Seattle	Washington	98195	US
Williams, L. Keoki	Henry Ford Health System		Detroit	Michigan	48202	US
Williams, Scott	Case Western Reserve University					
Wilson, Carla	Brigham & Women's Hospital		Boston	Massachusetts	02115	US
Wilson, James	Beth Israel Deaconess Medical Center	Cardiology	Cambridge	Massachusetts	02139	US
Winterkorn, Lara	New York Genome Center		New York City	New York	10013	US
Wong, Quenna	University of Washington		Seattle	Washington	98195	US
Wu, Joseph	Stanford University	Stanford Cardiovascular Institute	Stanford	California	94305	US
Xu, Huichun	University of Maryland		Baltimore	Maryland	21201	US
Yanek, Lisa	Johns Hopkins University		Baltimore	Maryland	21218	US
Yang, Ivana	University of Colorado at Denver		Denver	Colorado	80204	US
Yu, Ketian	University of Michigan		Ann Arbor	Michigan	48109	US
Zekavat, Seyedeh Maryam	Broad Institute		Cambridge	Massachusetts	02142	US
Zhang, Yingze	University of Pittsburgh	Medicine	Pittsburgh	Pennsylvania	15260	US
Zhao, Snow Xueyan	National Jewish Health		Denver	Colorado	80206	US
Zhao, Wei	University of Michigan	Department of Epidemiology	Ann Arbor	Michigan	48109	US
Zhu, Xiaofeng	Case Western Reserve University	Department of Population and Quantitative Health Sciences	Cleveland	Ohio	44106	US

Name	Institution(s)	Primary Department	Institution City	Institution State	Zip Code	Country
Ziv, Elad	University of California, San Francisco	Medicine	San Francisco	California	94143	US
Zody, Michael	New York Genome Center		New York	New York	10013	US
Zoellner, Sebastian	University of Michigan		Ann Arbor	Michigan	48109	US
