# Supplementary material for: Whole genome sequencing identifies structural variants contributing to hematologic traits in the NHLBI TOPMed program
Source: Nat Commun. 2022 Dec 8;13:7592. doi: 10.1038/s41467-022-35354-7 (PMC9732337; doi:10.1038/s41467-022-35354-7)
Supplement: Supplementary file 2 — Description of Additional Supplementary Files [file 41467_2022_35354_MOESM2_ESM.pdf]

Description of Additional Supplementary Files

File Name: Supplementary Data 1
Description: The number of participants grouped by predicted ancestry in each TOPMed Study. Discrete ancestry subgroups are based on genetically inferred ancestry and a machine learning algorithm that refines self-identified ancestry.

File Name: Supplementary Data 2
Description: Replication results for TOPMed structural variants significantly associated with hematological traits. All p-values are derived from two-sided t-tests and are not adjusted for multiple comparisons.

File Name: Supplementary Data 3
Description: Functional annotation for structural variants significantly associated with hematological traits.

File Name: Supplementary Data 4
Description: Summary of sgRNA sequences used in the present study.

File Name: Supplementary Data 5
Description: Summary of PCR primers used in the present study.

File Name: Supplementary Data 6
Description: Information on TOPMed cohorts used in this study.
