# Supplementary material for: Whole genome sequencing identifies structural variants contributing to hematologic traits in the NHLBI TOPMed program
Source: Nat Commun. 2022 Dec 8;13:7592. doi: 10.1038/s41467-022-35354-7 (PMC9732337; doi:10.1038/s41467-022-35354-7)
Supplement: Supplementary file 9 — Reporting Summary [file 41467_2022_35354_MOESM9_ESM.pdf]

nature portfolio

Corresponding author(s): Auer and Reiner

Last updated by author(s): Nov 21, 2022

Reporting Summary

Nature Portfolio wishes to improve the reproducibility of the work that we publish. This form provides structure for consistency and transparency in reporting. For further information on Nature Portfolio policies, see our Editorial Policies and the Editorial Policy Checklist.

Statistics

For all statistical analyses, confirm that the following items are present in the figure legend, table legend, main text, or Methods section.

n/a	Confirmed	
☐	☒	The exact sample size (*n*) for each experimental group/condition, given as a discrete number and unit of measurement
☐	☒	A statement on whether measurements were taken from distinct samples or whether the same sample was measured repeatedly
☐	☒	The statistical test(s) used AND whether they are one- or two-sided<br>*Only common tests should be described solely by name; describe more complex techniques in the Methods section.*
☐	☒	A description of all covariates tested
☐	☒	A description of any assumptions or corrections, such as tests of normality and adjustment for multiple comparisons
☐	☒	A full description of the statistical parameters including central tendency (e.g. means) or other basic estimates (e.g. regression coefficient) AND variation (e.g. standard deviation) or associated estimates of uncertainty (e.g. confidence intervals)
☐	☒	For null hypothesis testing, the test statistic (e.g. F, t, r) with confidence intervals, effect sizes, degrees of freedom and P value noted<br>*Give P values as exact values whenever suitable.*
☒	☐	For Bayesian analysis, information on the choice of priors and Markov chain Monte Carlo settings
☒	☐	For hierarchical and complex designs, identification of the appropriate level for tests and full reporting of outcomes
☒	☐	Estimates of effect sizes (e.g. Cohen's *d*, Pearson's *r*), indicating how they were calculated

Our web collection on statistics for biologists contains articles on many of the points above.

Software and code

Policy information about availability of computer code

Data collection	No software was used for data collection.
Data analysis	Data analyses were performed with the R (version 4.1.1) statistical computing platform and the GENESIS Bioconductor package. Code to implement the associations analyses is available on GitHub at https://github.com/UW-GAC/analysis_pipeline.

For manuscripts utilizing custom algorithms or software that are central to the research but not yet described in published literature, software must be made available to editors and reviewers. We strongly encourage code deposition in a community repository (e.g. GitHub). See the Nature Portfolio guidelines for submitting code & software for further information.

Data

Policy information about availability of data

All manuscripts must include a data availability statement. This statement should provide the following information, where applicable:

- Accession codes, unique identifiers, or web links for publicly available datasets
- A description of any restrictions on data availability
- For clinical datasets or third party data, please ensure that the statement adheres to our policy

TOPMed data are available via controlled access through the NIH database of genotypes and phenotypes (dbGaP). Data for each participating study can be accessed through dbGaP with the corresponding accession numbers (Amish, phs000956; ARIC, phs001211; BioMe, phs001644; CARDIA, phs001612; CHS, phs001368; COPDGene, phs000951; FHS, phs000974; GeneSTAR, phs001218; HCHS/SOL, phs001395; JHS, phs000964; MESA, phs001416; WHI, phs001237).

Field-specific reporting

Please select the one below that is the best fit for your research. If you are not sure, read the appropriate sections before making your selection.

☒ Life sciences ☐ Behavioural & social sciences ☐ Ecological, evolutionary & environmental sciences

For a reference copy of the document with all sections, see nature.com/documents/nr-reporting-summary-flat.pdf

Life sciences study design

All studies must disclose on these points even when the disclosure is negative.

Sample size	Sample size was determined as the largest set of samples with whole-genome sequence data and available blood-cell count phenotypes in the TOPMed study.
Data exclusions	Samples with extreme values for blood-cell phenotypes were excluded.
Replication	We sought replication of our findings in with data from deCODE genetics and the UKBiobank. Most of our primary genetic associations were replicated, for the associations that did not replicate, the variants were either absent or very rare in deCODE and UKBiobank.
Randomization	There was no randomization in our study because there was no intervention with subjects.
Blinding	Blinding was not relevant because there was no intervention with subjects.

Reporting for specific materials, systems and methods

We require information from authors about some types of materials, experimental systems and methods used in many studies. Here, indicate whether each material, system or method listed is relevant to your study. If you are not sure if a list item applies to your research, read the appropriate section before selecting a response.

Materials & experimental systems

n/a	Involved in the study	
☐	☒	Antibodies
☐	☒	Eukaryotic cell lines
☒	☐	Palaeontology and archaeology
☐	☒	Animals and other organisms
☐	☒	Human research participants
☒	☐	Clinical data
☒	☐	Dual use research of concern

Methods

n/a	Involved in the study	
☒	☐	ChIP-seq
☒	☐	Flow cytometry
☒	☐	MRI-based neuroimaging

Antibodies

Antibodies used	Anti-human CD45 (clone HI30, Cat# 560367, 1:200), anti-human CD34 (clone 581, Cat# 343504, 1:200), anti-human CD33 (clone P67.6, Cat# 366608, 1:200), anti-human CD117 (clone 104D2, Cat# 313205, 1:200), anti-human CD16 (clone 3G8, Cat# 302046, 1:100), anti-human CD14 (clone 63D3, Cat# 367117, 1:200), anti-human CD235a (clone HI264, Cat# 349104, 1:100), anti-human CD3 (clone UCHT1, Cat# 300412, 1:200), anti-human CD19 (clone HIB19, Cat# 302212, 1:200), and anti-mouse CD45 (clone 30-F11, Cat# 561487, 1:100).
Validation	All antibodies have been validated by BioLegend or BD (see website). Part of the antibodies were also validated in our previous work (Wu et al., 2019; Rao et al., 2021).

Eukaryotic cell lines

Policy information about cell lines

Cell line source(s)	THP-1 cells (Cat# TIB-202) were obtained from the American Type Culture Collection.
Authentication	None of the cell lines have been authenticated.
Mycoplasma contamination	Cell lines were not tested for mycoplasma contamination but no indication of contamination was observed.
Commonly misidentified lines (See ICLAC register)	No commonly misidentified cell lines were used.

Animals and other organisms

Policy information about studies involving animals; ARRIVE guidelines recommended for reporting animal research

Laboratory animals	NOD.Cg-Kit W-41J Tyr + Prkdc scid Il2rg tm1Wjl (NBSGW) female mice4-5weeksofage (JacksonLaboratory, BarHarbor, Maine) were kept in 12 hours of light at room temperature.
Wild animals	No wild animals were used in this study.
Field-collected samples	No field-collected samples were used in this study.
Ethics oversight	The mouse work was performed, following Boston Children's Hospital institutional review board (IRB) approval.

Note that full information on the approval of the study protocol must also be provided in the manuscript.

Human research participants

Policy information about studies involving human research participants

Population characteristics	The primary study population included participants in 12 TOPMed studies, the deCODE genetics project, and the UKBiobank. Population characteristics and full descriptions of the design and sampling for each study are included in the Supplementary Methods.
Recruitment	Full descriptions of the recruitment and design of each contributing study can be found in the Supplementary Methods.
Ethics oversight	For the Amish study, all study protocols were approved by the institutional review board at the University of Maryland Baltimore. All ARIC participants provided written informed consent, and the study was approved by the Institutional Review Boards of the participating centers. The BioMe cohort was approved by the Institutional Review Board at the Icahn School of Medicine at Mount Sinai. All BioMe participants provided written, informed consent for genomic data sharing. All CARDIA participants provided informed consent, and the study was approved by the Institutional Review Boards of the University of Alabama at Birmingham and the University of Texas Health Science Center at Houston. All CHS participants provided informed consent, and the study was approved by the Institutional Review Board [or ethics review committee] of University Washington. All COPDGene participants provided written informed consent, and the study was approved by the Institutional Review Boards of the participating clinical centers. The Framingham Heart Study was approved by the Institutional Review Board of the Boston University Medical Center. All GeneSTAR participants provided written informed consent and the study was approved by the Johns Hopkins Medicine Institutional Review Board. The HCHS/SOL study was approved by the institutional review boards (IRBs) at each field center, where all participants gave written informed consent, and by the Non-Biomedical IRB at the University of North Carolina at Chapel Hill, to the HCHS/SOL Data Coordinating Center. All IRBs approving the study are: Non-Biomedical IRB at the University of North Carolina at Chapel Hill. Chapel Hill, NC; Einstein IRB at the Albert Einstein College of Medicine of Yeshiva University. Bronx, NY; IRB at Office for the Protection of Research Subjects (OPRS), University of Illinois at Chicago. Chicago, IL; Human Subject Research Office, University of Miami. Miami, FL; Institutional Review Board of San Diego State University. San Diego, CA. The JHS study was approved by Jackson State University, Tougaloo College, and the University of Mississippi Medical Center IRBs, and all participants provided written informed consent. All MESA participants provided written informed consent, and the study was approved by the Institutional Review Boards at The Lundquist Institute (formerly Los Angeles BioMedical Research Institute) at Harbor-UCLA Medical Center, University of Washington, Wake Forest School of Medicine, Northwestern University, University of Minnesota, Columbia University, and Johns Hopkins University. All WHI participants provided informed consent and the study was approved by the Institutional Review Board (IRB) of the Fred Hutchinson Cancer Research Center. All participants in deCODE who donated samples gave informed consent and the National Bioethics Committee of Iceland approved the study which was in agreement with conditions issued by the Data Protection Authority of Iceland (VSN_140-015). Personal identities of the participant's data and biological samples were encrypted by a third-party system (Identify Protection System), approved and monitored by the Data Protection Authority

Note that full information on the approval of the study protocol must also be provided in the manuscript.
